# Reducing stillbirths: interventions during labour

**DOI:** 10.1186/1471-2393-9-S1-S6

**Published:** 2009-05-07

**Authors:** Gary L Darmstadt, Mohammad Yawar Yakoob, Rachel A Haws, Esme V Menezes, Tanya Soomro, Zulfiqar A Bhutta

**Affiliations:** 1 Department of International Health, The Johns Hopkins Bloomberg School of Public Health, Baltimore, Maryland, USA; 2 Division of Maternal and Child Health, The Aga Khan University, Karachi, Pakistan

## Abstract

**Background:**

Approximately one million stillbirths occur annually during labour; most of these stillbirths occur in low and middle-income countries and are associated with absent, inadequate, or delayed obstetric care. The low proportion of intrapartum stillbirths in high-income countries suggests that intrapartum stillbirths are largely preventable with quality intrapartum care, including prompt recognition and management of intrapartum complications. The evidence for impact of intrapartum interventions on stillbirth and perinatal mortality outcomes has not yet been systematically examined.

**Methods:**

We undertook a systematic review of the published literature, searching PubMed and the Cochrane Library, of trials and reviews (N = 230) that reported stillbirth or perinatal mortality outcomes for eight interventions delivered during labour. Where eligible randomised controlled trials had been published after the most recent Cochrane review on any given intervention, we incorporated these new trial findings into a new meta-analysis with the Cochrane included studies.

**Results:**

We found a paucity of studies reporting statistically significant evidence of impact on perinatal mortality, especially on stillbirths. Available evidence suggests that operative delivery, especially Caesarean section, contributes to decreased stillbirth rates. Induction of labour rather than expectant management in post-term pregnancies showed strong evidence of impact, though there was not enough evidence to suggest superior safety for the fetus of any given drug or drugs for induction of labour. Planned Caesarean section for term breech presentation has been shown in a large randomised trial to reduce stillbirths, but the feasibility and consequences of implementing this intervention routinely in low-/middle-income countries add caveats to recommending its use. Magnesium sulphate for pre-eclampsia and eclampsia is effective in preventing eclamptic seizures, but studies have not demonstrated impact on perinatal mortality. There was limited evidence of impact for maternal hyperoxygenation, and concerns remain about maternal safety. Transcervical amnioinfusion for meconium staining appears promising for low/middle income-country application according to the findings of many small studies, but a large randomised trial of the intervention had no significant impact on perinatal mortality, suggesting that further studies are needed.

**Conclusion:**

Although the global appeal to prioritise access to emergency obstetric care, especially vacuum extraction and Caesarean section, rests largely on observational and population-based data, these interventions are clearly life-saving in many cases of fetal compromise. Safe, comprehensive essential and emergency obstetric care is particularly needed, and can make the greatest impact on stillbirth rates, in low-resource settings. Other advanced interventions such as amnioinfusion and hyperoxygenation may reduce perinatal mortality, but concerns about safety and effectiveness require further study before they can be routinely included in programs.

## Introduction

Stillbirths, or late fetal deaths, account for more than half of the world's 6 million perinatal deaths that occur in low-/middle-income countries each year. While stillbirth rates are commonly as low as 3 to 5 per 1000 births in some high-income countries, their incidence is estimated to be five to ten times greater in many low-/middle-income countries. These higher stillbirth rates are believed to be attributable to poor baseline maternal health (especially nutritional status), poor prevention and treatment of maternal conditions and infections during pregnancy, and inappropriate management of complications during pregnancy and childbirth.

There are two kinds of intrauterine fetal deaths: those that occur prior to the onset of labour (antepartum stillbirths), and those that occur during labour (intrapartum stillbirths). The major causes of antepartum stillbirths are pregnancy complications that lead to fetal asphyxia and/or infection, including maternal infections, hypertensive disorders, placental dysfunction and haemorrhage, and fetal or placental abnormalities. The specific causes of many antepartum stillbirths, however, are difficult to ascertain. In low-/middle-income countries, approximately one-third of stillbirths are estimated to occur intrapartum, and these are caused primarily by complications arising during labour and childbirth, such as prolonged or obstructed labour or umbilical cord accidents [1,2].

Intervention strategies to prevent antepartum and intrapartum stillbirths differ because they have largely different causes. Where women receive quality intrapartum care, as in many high-income countries, the proportion of intrapartum stillbirths is less than 10% of all stillbirths [2], indicating that a substantial proportion of intrapartum stillbirths are preventable with quality intrapartum care. Yet half of the world's women give birth at home, in the absence of a skilled birth attendant. Globally, the intrapartum stillbirth rate is estimated to be between 7 and 9 per 1000 births [2-4], but this figure obscures wide disparities both within and among countries, including substantial urban-rural and rich-poor divides. The risk of an intrapartum stillbirth in low and middle-income countries is more than 14 times that in high-income countries; the risk is 17 times higher in low-income than in middle-income countries. When women in low-/middle-income countries do give birth in health facilities, their care and their pregnancy outcomes are frequently compromised by absent or overburdened health care providers; deficiencies in training and supervision; insufficient supplies, drugs, and equipment; and substandard hygienic practices.

This paper focuses on interventions delivered during childbirth, primarily care provided at secondary- and tertiary-level large teaching and research hospitals with surgical capacity, that are of potential benefit for perinatal health and prevention of stillbirths.

## Methods

Detailed methods undertaken to assemble and assess the evidence for the interventions in this paper are given in the first paper in this series [5]. Each study was given a level of evidence (LOE) according to the SIGN grading system. Grade of evidence, (i.e. A, B, C, D) was determined and then the interventions were classified under clear evidence, some evidence, uncertain evidence or evidence of no or negative impact, as detailed in paper 1 [5]. We included 8 obstetric care interventions in our analysis (Table 1).

**Table 1:** Interventions to prevent intrapartum stillbirth reviewed in this paper

• Instrumental delivery (vacuum and forceps-assisted)
• Emergency obstetric care, including Caesarean section
• Induction of labour versus expectant management
• Drugs for cervical ripening and induction of labour
• Planned Caesarean section for breech presentation
• Magnesium sulphate for treatment of pre-eclampsia/eclampsia or pre-term labour
• Maternal hyperoxygenation for suspected impaired fetal growth
• Amnioinfusion

## Results

### Instrumental delivery (vacuum and forceps-assisted)

#### Background

Instrumental vaginal deliveries, which make up a subset of operative deliveries, are procedures involving traction applied to the fetal head, for indications including maternal exhaustion or other compromise (e.g., maternal heart disease), fetal distress or heart rate abnormalities (often associated with prolonged second stage of labour), and fetal malposition [6]. Rates of instrumental deliveries range from 5–20% in high-income countries [6]. Before an instrumental delivery should be attempted, the fetal head must be engaged and its position known, the cervix fully dilated, and the membranes ruptured. Traction is generally applied to the fetal head with either forceps or a vacuum extraction device (also called a *ventouse*). The use of these devices poses the potential for injury to the mother or the baby. Forceps have long been associated with birth trauma, particularly when used for rotational procedures, but low overall morbidity rates are reported for well-trained forceps practitioners [7]. While the association with birth injury is fairly well established, whether forceps use or vacuum extraction is preferable for preventing stillbirth or perinatal mortality is unclear.

#### Literature-based evidence

Our systematic review included 2 Cochrane reviews and 5 other observational and intervention studies (Table 2). No studies were found that compared stillbirth outcomes with versus without instrumental vaginal delivery. One Cochrane review by Johanson et al. [7] compared the impact of vacuum extraction to forceps delivery, and included 7 trials reporting perinatal mortality outcomes (Additional file 1). Vacuum extraction compared to forceps delivery was associated with significantly less maternal trauma (OR = 0.41, 95% CI: 0.33–0.50) and less general and regional anaesthesia. Additionally, the risk of Caesarean section showed a trend towards being lower among the vacuum extractor group than the forceps group (OR = 0.56, 95% CI: 0.31–1.02 [NS]). Vacuum extraction was more likely to fail when used for assisted vaginal delivery than forceps (OR = 1.69, 95% CI: 1.31–2.19). Perinatal mortality rates were not statistically significantly different between the two instrumental methods (7 trials, N = 1800, OR = 0.80, 95% CI: 0.18–3.52 vacuum extraction vs. forceps, respectively). The vacuum extractor was associated with an increase in neonatal cephalhaemotomas and retinal haemorrhages, but serious neonatal injury was uncommon with either instrument and Apgar scores at 1 and 5 min were comparable ***[LOE: 1+]***. The other Cochrane review, also by Johanson et al. [8], compared the impact of use of soft versus rigid vacuum extractor cups; only one trial (N = 72) in Malaysia reported perinatal death as an outcome but found no significant difference between soft and hard cups (OR = 1.26, 95% CI: 0.08–20.85 [NS]) ***[LOE: 1+] ***(Additional file 2).

**Table 2:** Comparison of impact of vacuum extractor versus forceps on stillbirths and perinatal mortality

**Source**	**Location and Type of Study**	**Intervention**	**Stillbirths/Perinatal Outcomes**
***Reviews and meta-analyses***			

Johanson and Menon 1999 [7]	USA, Denmark, Sweden, England, South Africa. Meta-analysis (Cochrane). 7 RCTs included (N = 1800 women).	Assessed the effects of vacuum extraction vs. forceps on maternal and neonatal morbidity.	PMR: OR = 0.80 (95% CI: 0.18–3.52) **[NS]**. [3/901 vs. 4/899 in vacuum vs. forceps group, respectively].

Johanson and Menon 2000 [8]	Malaysia. Cochrane review. 1 RCT included with death as outcome (N = 72 women).	To assess the effects of soft (intervention) vs. rigid vacuum extractor cups (control) on perineal injury, fetal scalp injury and success rate.	Death: OR = 1.26 (95% CI: 0.08–20.85) **[NS]**. [1/32 vs. 1/40 in intervention and control groups, respectively].

***Intervention studies***			

Mustafa and Mustafa 2002 [10]	Pakistan (Multan). Nishtar Hospital. RCT. Consecutive patients (N = 931), of which 50 were selected (N = 27 ventouse group, N = 23 forceps). 50/931 consecutive patients were randomly selected either to forceps delivery (Group I) or ventouse extraction (Group II).	Compared the effects of ventouse vs. forceps delivery on maternal and perinatal outcome.	SBR: 0/27 vs. 1/23 in the vacuum and forceps groups, respectively. Success rate: 26/27 (96.30%) vs. 22/23 (95.65%) in vacuum and forceps groups, respectively. There was one failure in each category which was later on delivered by Caesarean section.

Weerasekera et al. 2002 [9]	Sri Lanka. Tertiary care setting. RCT. Women (N = 442) undergoing instrumental delivery in the second stage (N = 238 forceps group, N = 204 vacuum).	Compared the impact of forceps vs. vacuum delivery on the stillbirth rate.	SBR or NMR: 1/238 vs. 1/204 in the forceps and vacuum groups, respectively; P > 0.05. There was no significant difference in babies needing resuscitation at birth or admission to neonatal intensive care unit.

***Observational studies***			

Broekhuizen et al. 1987 [11]	USA. Tertiary care setting. Retrospective study. N = 256 vacuum extractions, and N = 300 randomly chosen forceps deliveries were analyzed.	Compared the impact of the vacuum extraction vs. forceps deliveries.	Death: one event in each group.

Gachiri et al. 1991 [13]	Kenya (Nairobi). Kenyatta National Hospital. Prospective study. Vacuum extractions (N = 167).	Assessed the fetal and maternal outcome among women undergoing vacuum extraction	SBR: 6/167 (3.6%). PMR: 8/167 (4.8%).

Mesleh 2002 [136]	Saudi Arabia. Retrospective review. Vaginal deliveries (N = 304) with instrument use (N = 258 ventouse group, N = 46 forceps).	Assessed the effects of vacuum vs. forceps deliveries on pregnancy outcomes.	SBR: 1/258 vs. 0/46 in the forceps and ventouse groups, respectively. The single stillbirth in the vacuum delivery group was due to intrapartum asphyxia and true knot in the umbilical cord.

An RCT by Weerasekera et al. [9] not included in the above reviews compared the outcomes associated with vacuum and forceps deliveries (N = 442 women) in the second stage of labour. There were no significant differences between the two methods in the incidence of third-degree perineal tears, postpartum haemorrhage or ruptured uterus, but cervical tears were slightly higher in the forceps group. Cephalhaematoma incidence was higher among the vacuum extraction group, but there were no significant differences between the groups in babies needing resuscitation at birth, admission to neonatal intensive care unit, stillbirth or neonatal death rates ***[LOE: 2+]***. Another RCT from Pakistan reported similar success rates with both instruments [10].

Multiple observational studies have evaluated the complications and outcomes related to vacuum deliveries either alone or in comparison with alternative methods of instrumental deliveries [11,12]. In a prospective study of 167 vacuum extractions (6.3% of total deliveries) at the national hospital in Kenya, Gachiri et al. [13] documented perinatal morbidity and mortality rates of 16.2% and 4.8% respectively ***[LOE: 3]***. Lurie et al [14] conducted a decision-to-delivery time analysis and found that it was faster to undertake forceps deliveries compared to vacuum extractions. While some studies suggested higher rates of vaginal tears with forceps [15], in general no differences in complication rates were found in other studies [16], and no studies found differences in stillbirth incidence.

#### New meta-analysis

**Figure 1:**
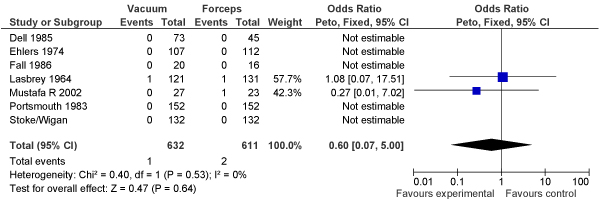
**Results of new meta-analysis of impact of vacuum versus forceps delivery on stillbirths**.

We identified 7 trials comparing vacuum extraction versus forceps-assisted deliveries that reported stillbirth incidence (N = 632 vacuum, N = 611 forceps). Our meta-analysis found no evidence of differential impact of either method on stillbirths (OR = 0.60, 95% CI: 0.07–5.00) (Figure 1).

#### Conclusion

Although vacuum extraction was associated with a trend toward lower Caesarean section rates and fewer significant maternal injuries and less anaesthetic requirement than forceps delivery, there was no difference in rates of intrapartum stillbirth or perinatal mortality. Vacuum extraction tended to be associated with increased, albeit low, risk of neonatal cephalohaematoma and retinal haemorrhage. The lower rate of Caesarean section despite higher failure rate among vacuum extractions may be due to superiority of the vacuum for managing certain fetal malpositions (such as deflexed occipital posterior position, for example), or more likely, because following a failed vacuum extraction, delivery is usually by forceps, while failed forceps is usually followed by Caesarean section [7,17]. The reduced maternal morbidity and limited, largely short-term risk of neonatal complications associated with vacuum extraction suggest that although there is no evidence that either method is superior to the other in preventing stillbirths, vacuum extraction may be preferable in areas where vacuum extractors are available and practitioners are suitably trained to perform vacuum extraction [17]. In other areas where forceps deliveries are the norm and health practitioners do not have training in vacuum extraction, the significant investment needed for the purchase of vacuum extractors and quality training to capacitate practitioners to perform the procedure may be prohibitive for certain low-resource settings. Relatively inexpensive manual vacuum extractors are available that may require less training to use than forceps and may be a useful alternative to forceps to facilitate rapid delivery in the presence of signs of fetal distress in the second stage of labour; however, these require further evaluation.

### Emergency obstetric care, including Caesarean section

#### Background

The vast majority of the world's one million intrapartum stillbirths each year occur in low-/middle-income countries marked by high rates of unmet obstetric need [18,19], suggesting that many of these deaths could be prevented with improved obstetric care [20,21]. The availability of comprehensive essential obstetric care (EOC), supported by emergency transport, skilled providers, and aftercare, is regarded as critical for effective maternal health services in obstetric emergencies [22]. Essential obstetric care (EOC) refers to elements of obstetric care needed for the management of normal and complicated pregnancy, delivery and the postpartum period. Basic EOC can be performed in primary health care facilities, and includes administration of anti-biotics, oxytocics, anti-convulsants including magnesium sulfate, manual removal of the placenta, treatment for incomplete miscarriage, post-abortion care, and instrumental vaginal delivery with forceps or vacuum extractor. Comprehensive EOC includes all basic EOC functions plus Caesarean section, anaesthesia, and blood transfusion, and generally requires a secondary or higher-level health facility. The subset of comprehensive EOC interventions used to respond to unexpected intrapartum complications such as haemorrhage and obstructed labour is referred to as emergency obstetric care (EmOC) [23], and includes some elements of basic EOC such as manual removal of the placenta and medical treatment in labour, as well as all anaesthesia, blood transfusion, and Caesarean section.

Non-availability of EmOC, especially Caesarean section, in resource-poor settings has been implicated as a risk factor for intrapartum stillbirths, particularly those associated with prolonged labour and its associated fetal asphyxia, infection, and birth trauma [24-28]. Reductions in intrapartum stillbirth rates observed in the UK have been attributed to more liberal use of Caesarean section [20], though this is controversial. There is evidence to suggest that the optimal Caesarean section rate to reduce the number and proportion of intrapartum stillbirths lies between 5 and 10 percent [3]. Recent data from global estimates of Caesarean section availability at population level indicate that many countries fall far short of this range, while others exceed this, to the possible detriment of maternal health outcomes [29]. Some studies have shown that it can be comparatively safe to deliver in rural hospitals even if they lack Caesarean section capability [30].

Quality of EmOC is also critical. These issues have been documented in high-income countries, particularly among rural populations [26,31,32], but the data associating quality of EmOC with stillbirth or perinatal mortality rates has not been compiled.

Because the components of EmOC are by definition life-saving interventions, RCTs of EmOC versus no EmOC would be unethical, so the evidence base for improving access to and quality of EmOC, especially Caesarean section, consists largely of observational studies.

#### Literature-based evidence

The literature on EmOC considered two primary issues: availability and optimal rates of Caesarean section (and associations with intrapartum stillbirth rates); and the impact of quality of obstetric care on perinatal outcomes. We identified 40 studies reporting stillbirth or perinatal mortality outcomes that reviewed or implemented interventions to provide EmOC (Table 3).

**Table 3:** Impact of emergency obstetric care on stillbirths and perinatal mortality

***Source***	***Location and Type of Study***	***Intervention***	***Stillbirths/Perinatal Outcomes***
***Quality and availability of obstetric care***			

Abdel-Latif et al; NICUS Group 2006 [57]	Australia (New South Wales and Australian Capital Territory). 10 neonatal intensive care units; stillbirth analysis done from other regional data. Retrospective analysis. Infants (N = 8654) < 32 wks' gestation born 1992–2002 (N = 1879 rural areas, N = 6775 urban). Regional SB analysis: N = 14,707 births.	Compared the impact of rural vs. urban residence and associated differentials in access to higher-level emergency obstetric care on perinatal mortality measures.	NMR (in NICU): adj. OR = 1.26 (95% CI: 1.07–1.48, P = 0.005) in rural vs. urban group. SBR: OR = 1.20 (95% CI: 1.09–1.32; P < 0.001). [727/3530 (20.6%) vs. 1991/11177 (17.8%) in rural and urban infants, respectively].

Cameron 1998 [25]	Australia (Far North Queensland). Atherton Hospital. Descriptive study. N = 2883 deliveries from 1981–1990 (N = 1974 public confinements, N = 909 private confinements).	Assessed annual obstetric audit data from 1981–1990 to compare publicly versus privately funded facilities.	PMR: 5.1/1000 vs. 5.5/1000 in public and private confinements, respectively. PMR (corrected): 9.6/1000 vs. 13.5/1000 vs. 16.9/1000 in public patients, Queensland (1987) and the Far North Statistical Division (1987), respectively.

Gaffney et al. 1994 [48]	UK (Oxford). National Perinatal Epidemiology Unit. Case control study. N = 573 participants, of whom N = 141 cerebral palsy group (N = 257 controls) and N = 62 perinatal deaths (N = 119 controls).	Compared the frequency of events during labour and delivery, and the suboptimal care among cases (with perinatal deaths) vs. controls.	Intrapartum haemorrhage: OR = 5.3 (95% CI: 1.4–20.1) in cases of deaths vs. controls. Meconium stained amniotic fluid: OR = 12.3 (95% CI: 3.6–41.4) in cases of deaths vs. controls. Failure to respond to signs of severe fetal distress: OR = 26.1 (95% CI: 6.2 – 109.7) in cases of deaths vs. controls.

Goldenberg et al. 2007 [3]	Review. Data from 51 countries (WHO and other sources).	Logistic regression analysis of measures of antenatal and obstetric care with perinatal outcomes.	Intrapartum SB: for each 1% increase in the percentage of women with at least 4 antenatal visits, the intrapartum SBR decreased by 0.16/1000 births (P < 0.0001). Intrapartum SB: as Caesarean section rates increased from 0 to 8%, for each 1% increase, the intrapartum SBR decreased by 1.61/1000 births. No relationship between Caesarean section and SBR in high-income countries. Stronger relationship between various measures of care with intrapartum versus antepartum SBR.

Grzybowski et al. 1991 [59]	British Columbia (Queen Charlotte Islands). 21-bed hospital and medical clinic. Descriptive study. All women (N = 286) >20 wks' gestation delivering from 1984–88. 33% were primiparous, 20% native. N = 192 (67%) delivered locally, N = 33 (12%) transferred after admission for complications, N = 61 (21%) delivered elsewhere.	Assessed the PMR among women delivering at a small hospital without Caesarean section capability delivering <50 infants per year.	PMR: 20.8 (95% CI: 4.4–37.2); N = 6. Adverse perinatal outcome: 6.2% (12/193 newborns) (95% CI: 2.8–9.6%).

Kiely et al. 1985 [56]	USA (New York City). Prospective study. All births of infants weighing > 1000 g from 1976–78.	Computed fetal mortality rates (adjusted for confounding by birth weight, gestational age, and other variables) at different levels of care.	Intrapartum SBR: 61% excess risk in Level 1 (community hospital) vs. Level 3 (perinatal intensive care) maternity units)(P > 0.01). Intrapartum SBR: 35% excess risk in Level 2 units (intermediate level of care) vs. Level 3 units (P = 0.06).

Korhonen and Kariniemi 1994 [54]	Finland. Prospective study. Cases of emergency Caesarean section (N = 101). N = 60 cases study group, N = 41 controls.	Compared the impact on survival of cases with the operating team in the hospital (study group) vs. cases with the team on call (outside the hospital) (controls).	Live birth/neonatal survival rate: significantly higher when the operating team was in the hospital vs. on call outside the hospital), P = 0.05. SBR: 0/60 vs. 3/41 in intervention vs. controls, respectively . Hypoxic ischemic encephalopathy: 1/41 in the controls.

Lansky et al. 2007 [50]	Brazil (Belo Horizonte). Population-based in 24 hospitals. Cohort study. N = 36,469 births, N = 419 perinatal deaths in 1999.	Compared PMR in hospitals contracted to the National Public Health System (SUS) with non-SUS hospitals.	PMR: OR = 2.92 (95% CI: 1.87–4.54) in the private-SUS vs. private non-SUS hospitals. PMR: OR = 1.81 (1.12–2.92) in the philanthropic-SUS vs. private non-SUS hospitals. PMR: OR = 1.30 (95% CI: 0.82–2.05) **[NS] **in the public SUS vs. private non-SUS hospitals.

Lansky et al. 2007 [49]	Brazil (Belo Horizonte). Cohort study. N = 40,953 births and N = 826 perinatal deaths in 1999.	Compared PMR in hospitals linked to the national Universal Public Health System (SUS) vs. non-SUS hospitals.	PMR: highest in private and philanthropic SUS-contracted hospitals relative to private, non-SUS-contracted hospitals. Quality of care also associated with PMR.

Leeman et al. 2002 [30]	USA (New Mexico). Native American hospital. Retrospective cohort study. All pregnant women (N = 1132) > 20 weeks gestation 1992–1996. N = 735 (64.7%) gave birth at the hospital without operative facilities; N = 290 (25.6%) were transferred before labour; and N = 107 (9.5%) were transferred during labour.	Compared the PMR at hospital lacking on-site Caesarean capability but with a high-risk obstetric population) with the nationwide PMR (historical controls).	PMR: 11.4/1000 (95% CI: 5.1–17.8) vs. 12.8/1000 at the hospital vs. nationwide, respectively **[NS]**. Caesarean section rate: 7.3% vs. 20.7% at the hospital and nationwide, respectively (statistically significant). Low Apgar score: 0.54% vs. 1.4% at the hospital and the nationwide, respectively (statistically significant). Resuscitation required: 3.4% vs. 2.9% at the hospital and nationwide, respectively **[NS]**.

Longombe et al. 1990 [41]	Zaire. Rural setting. Retrospective study. Total deliveries (N = 9947) during a five-year period. N = 8476 (85.2%) normal deliveries; N = 1014 (10.2%) Caesarean; N = 484 (4.9%) complicated vaginal deliveries.	Compared the impact on perinatal mortality in the Caesarean group (study group #1) vs. complicated vaginal deliveries (study group #2) vs. normal deliveries (comparison group).	PMR: 3.67% vs. 2.29% vs. 0.75% in study group #1, study group #2, and comparison group, respectively.

McClure et al; NICHD FIRST BREATH Study Group 2007 [137]	Democratic Republic of Congo, Guatemala, India, Zambia, Pakistan, Argentina. Population-based study, community-based. Prospective cohort study. N = 60,324 deliveries over an 18-month period.	Assessed care-based risk factors for SBR in different low-/middle-income countries.	SBR: 34/1000 vs. 9/1000 in Pakistan and Argentina, respectively. Maceration: 17.2% of stillbirths. Higher SBR significantly associated with less-skilled providers, out-of-hospital births, and low Caesarean section rates.

Rautava et al. 2007 [51]	Finland. 14 level II (central) and 5 level III (university) hospitals. Retrospective national medical birth-register study. N = 2291 very pre-term infants (gestational age <32 weeks at birth or birth weight of ≤ 1500 g) born from 2000–2003.	Compared PMR between level II (central) and level III (university) hospitals.	IMR + SBR: 494/2291 infants (21.6%). IMR: 224/2021 (11.1%) among live-born infants. Both the total 1-year mortality and the 1-year mortality of live-born infants were higher in level II hospitals compared with level III hospitals.

Steyn et al. 1998 [58]	South Africa. Hospital records. Retrospective analysis. N = 174,713 deliveries during 1975–1994), of which N = 22,773 were by Caesarean.	To describe trends in Caesarean section and PMR over the study period.	PMR: 34.7/1000 vs. 18.4/1000 in 1975 vs. 1994, respectively. The Caesarean section rate stayed constant at about 13% during this period.

***Practice of Caesarean section and impact on perinatal mortality***			

Bottoms et al. 1997 [37]	USA. Academic referral centers with neonatal intensive care units. Prospective observational study. Singleton extremely low birth weight (LBW) infants (N = 713) over a one-year study period (N = 482 study group, N = 231 controls).	Compared the impact on PMR of provider willingness to perform Caesarean delivery at 24 weeks for indications of fetal distress (intervention) vs. provider unwillingness to provide early Caesarean for these indications (controls).	Neonatal survival: adj. OR = 3.7 (95% CI: 2.3–6.0); P = 0.0001 in the study vs. control group, respectively. Survival without serious neonatal morbidity: OR = 1.8 (94% CI: 1.0 = 3.3) **[NS] **in the study vs. control group, respectively. SB: 19.5% vs. 0% for 21 weeks vs. > 28 weeks, respectively. NMR: 78% vs. 3.3% in 21 weeks and 30+ weeks, respectively.

De Muylder and Amy 1993 [40]	Zimbabwe (the Midlands Province). 12 hospitals. Prospective study. Deliveries from 1985–1986 in 12 hospitals (N = 19,363 deliveries/year), with Caesarean section rates ranging from 2.2–16.8%.	To assess the impact of high versus low Caesarean section rates on perinatal outcome.	PMR: 51.9/1000 vs. 39.7/1000 births in 6 hospitals with high rate of Caesarean section vs. 6 hospitals with high rate of instrumental delivery, respectively. Statistically significantly higher in hospitals with instrumental:Caesarean section ratio < 0.2. Caesarean section and PMR positively correlated: R^2 ^= 0.429 (P = 0.021).

Hankins et al. 2006 [36]	USA. Review.	To assess the impact on neonatal morbidity and mortality in a high-income country setting of allowing women to opt for delivery by elective Caesarean section at 39 weeks of gestation.	Extracted findings from reviewed studies: SBR: steady from 23–40 wks gestation, 5% of all stillbirths occurring at each week of gestation (Copper). SBR: 0.6/1000 vs. 1.9/1000 live births at 33–39 wks vs. >39 wks' gestation (Yudkin). SBR: 1.3–4.6/1000 live births from 37–41 wks' gestation (Fretts). Estimated prevention of SB associated with elective Caesarean for all births at 39 wks: 2/1000 living fetuses (6000 SBs prevented in the US each year).

Iffy et al. 1994 [38]	Ireland (Dublin) and USA (Newark, New Jersey). 2 large hospitals. Observational study. N = 68479 births (excluding malformations). Caesarean section rates: 6% in Ireland hospital, 17.5% at USA hospital.	Compared the PMR associated with different Caesarean section rates at 2 different hospitals.	PMR: 611/50768 (12.0/1000) vs. 343/17711 (19.8/1000) in Newark vs. Dublin, respectively; P < 0.01. NMR: No impact.

Ilesanmi et al. 1996 [47]	Nigeria (Ibadan). Oluyoro Catholic Hospital. Descriptive study. Breech singleton deliveries (N = 441 of 21,243 deliveries).	Compared the intrapartum stillbirth rate associated with breech (study group) vs. cephalic deliveries (controls).	Fresh SBR: 7.8% vs. 1.2% for breech and cephalic, respectively over the same time period. Caesarean section performed for 15.7% of breech singleton deliveries (indicatioN = fetal distress).

McClure et al. 2007 [21]	188 developed and developing countries. WHO data. Regression analysis.	To analyze correlation between SBR and multiple measures of antenatal and obstetric care (Caesarean section rates, skilled delivery attendance, and complete ANC).	SBR and MMR: strongly correlated, ~5 SBs for each maternal death. Ratio: 2:1 in least developed countries vs. 50:1 in the most developed countries. SBR: Decreased sharply as Caesarean section rates increased from 0 to about 10%, (same for MMR). SBR: No significant reductions associated with skilled attendance until coverage rates ~40%. SBR: No reductions associated with complete ANC until 60% coverage was achieved (modest reduction).

Mekbib and Teferi 1994 [138]	Ethiopia (Addis Ababa). Hospital-based study. Retrospective review of hospital records. N = 11,657 consecutive deliveries 1987–1992). N = 645 Caesarean sections (5.5%).	Compared the impact PMR of deliveries by Caesarean section vs. all deliveries (controls).	PMR: 153.5/1000 (N = 99) vs. 67.5/1000 live births in Caesarean section group vs. rate for all deliveries respectively (P < 0.01).

O'Driscoll et al. 1988 [39]	USA (Dallas, TX) and Ireland (Dublin). Retrospective analysis of hospital records from 1982–84. Pregnant women admitted to hospital (N = 24441 at Dublin; N = 22580 women in Dallas).	Compared the impact on PMR between a low Caesarean section rate hospital (Dublin) vs. a high Caesarean section rate hospital (Dallas).	CS rates: 482/8068 (6.0%) vs. 2001/10988 (18.0%) in Dublin vs. Dallas, respectively in 1983. [330/7782 (4.2%) vs. 2022/11592 (17.3%) in Dublin vs. Dallas, respectively in 1984.] PMR: 148/8199 (17.9/1000) vs. 161/11098 (14.5/1000) in Dublin vs. Dallas, respectively in 1983. [119/7879 (15.1/1000) vs. 207/11716 (17.8/1000) at Dublin and Dallas, respectively in 1984.] Intrapartum SB: 7-fold lower in Dallas compared to Dublin in 1983. Including 1982 & 1984, no significant difference in overall PMR despite 4 times as many Caesareans in Dallas as Dublin.

Wright et al. 1991 [139]	Nigeria. Jos University Teaching Hospital (high-risk population). Descriptive study. N = 757 patients undergoing Caesarean section.	Assessed PMR among a case series of Caesarean section.	PMR: 235/1000 (N = 69 stillbirths and N = 107 early neonatal deaths). Caesarean section rate: 4.4%.

***Management of risk factors for stillbirth***			

Abate et al. 2006 [140]	Ethiopia (Addis Ababa). Two teaching hospitals. Retrospective study. Eclamptic cases (N = 216) diagnosed, admitted and managed from October 1994 to September 1999.	To assess the stillbirth rate (SBR) and perinatal mortality rate (PMR) among women admitted to hospital who presented with or who developed eclampsia.	SBR: 44/216. Early neonatal deaths: 25/216. PMR: 312.2/1000 deliveries.

Alessandri et al. [35]	Australia (Western Australia). Matched case-control study. Intrapartum stillbirths ≥1000 g (cases) and live born infants (controls) matched for year of birth (1980–1983), plurality, sex, birth weight, and race of mother.	To determine antenatal and intrapartum risk factors for intrapartum stillbirths at the population level.	Placental abruption: OR = 9.55 (95% CI: 2.09–43.69) in cases versus controls, respectively. Fetal distress: OR = 4.64 (95% CI: 1.92–11.19) in cases versus controls, respectively. Cord prolapse: OR = 10.00 (95% CI: 1.17–85.60) Placental problems (OR = 2.26, 95% CI: 1.13–4.52) Vaginal breech delivery: OR = 3.51 (95% CI: 1.40–8.80) and Emergency Caesarean section: OR = 2.15 (95% CI: 1.13–4.10). No antenatal risk factors predicted deaths.

Basso et al. 2006 [141]	Norway. Population-based using data from the Medical Birth Registry. Longitudinal study. Singleton firstborn fetuses (N = 804,448) with Norwegian-born mothers born 1967–2003.	Compared the impact on perinatal outcomes of being born to preeclamptic (exposed) vs. non-preeclamptic (unexposed) mothers in the period from 1991–2003 vs. 1967–1978.	SBR: OR = 1.3 (95% CI: 1.1–1.7) in exposed vs. unexposed group, respectively from 1991–2003 vs. adj. OR = 4.2 (95% CI: 3.8–4.7) in exposed vs. unexposed group from 1967–78. Induction before 37 weeks for preeclampsia: 20% vs. 8% in 1991–2003 vs. 1967–78, respectively.

Bhattacharyya et al. 1979 [142]	India. Prospective study. Patients (N = 60) with previous stillbirths. A majority (75%) had a history of repeated stillbirths, and responsible pathology was detected in 55% of the cases.	To assess the impact of active antepartum, intrapartum, and early postnatal care in women with previous stillbirths.	Live birth: 75%.

Cruikshank and Linyear 1987 [53]	USA (Virginia). Perinatal audit. N = 108 term fetal deaths in 1983.	Assessed circumstances and management of term fetal deaths occurring in Virginia to determine potential preventability.	Preventable fetal death: 52/108 (48%) Risk factors for antepartum fetal death: maternal hypertension, diabetes, inadequate fetal surveillance, post-term pregnancy Major cause of intrapartum fetal death: delay between obvious fetal compromise onset and delivery. Incidence of preventable term stillbirth lower in larger hospitals.

Onyiriuka 2006 [143]	Nigeria (Benin City). Retrospective, observational study. All babies born weighing > 4000 g.	Compared the fetal outcome in high birth weight babies (study group) vs. normal weight babies (controls).	Risk of fetal death higher in high birth weight babies (full text not available). Risk of Caesarean section higher in high birth weight babies (full text not available).

***Management and mortality of twin delivery, including impact of Caesarean section***			

Ananth et al. 2004 [144]	USA. Retrospective cohort study. Twin live births and stillbirths between 1989–91 and 1997 = 99 (N = 1,102,212).	Compared the changes in the SBR (≥22 weeks), labour induction, and Caesarean rates among twin births from 1989–91 and 1997–99.	SBR: RR = 0.52 (95% CI: 0.49–0.55) [13.9/1000 vs. 24.4/1000 in 1999 vs. 1989, respectively (48% decrease).] SBR excluding births weighing < 500 g and adjusting for changes in labour induction and Caesarean delivery: RR = 0.75 (95% CI: 0.72–0.79)(25% decrease). Labour induction: 13.8% vs. 5.8% in 1997–99 vs. 1989–91, respectively (138% increase). Caesarean delivery: 55.6% vs. 48.3% in 1997–99 vs. 1989–91, respectively (15% increase).

Fakeye 1988 [145]	Nigeria (Ilorin). University of Ilorin Teaching Hospital. Descriptive study. Consecutive twin pairs (N = 622). N = 146 twin-1 and N = 192 twin-2 breech births.	Compared PMR between first and second twin breech infants.	SB and asphyxia (Apgar 1,2, or 3) high in both first and second twin breech infants. PMR: 13.7% vs. 18.8% for twin-1 and twin-2 breech, respectively. Corrected PMR: 9.3% vs. 12.4% for twin-1 and twin-2 respectively among infants weighing 2.0 kg or more. Twin-specific breech PMR lowest in 2.5–2.9 kg group (higher for smaller and larger twins). Breech-breech or primary breech managed by Caesarean section: lower PMR than vaginally delivered breech twin pairs.

Rydhstrom and Ingemarsson 1991 [146]	Sweden (Stockholm). The National Medical Birth Registry. Matched case-control twin study. N = 273 twin pregnancies delivered 1973–1983 weighing 1500–2499 g. N = 91 pregnancies (cases), N = 182 controls.	To compare the Caesarean section rates between the cases where one or both twins died vs. controls with similar birth weight (+/- 100 g) and year of delivery (+/- 1 year).	Caesarean section rate: 20% vs. 50–65% in 1973–76 vs. 1981–83 respectively, with an increase for both cases and controls. No significant difference between groups [NS]

Smith et al. 2005 [147]	UK (Scotland). Retrospective cohort study. All twin births (N = 8073) ≥36 weeks of gestation, excluding antepartum stillbirths and perinatal deaths due to congenital abnormality, 1985–2001; of which N = 1472 deliveries by planned Caesarean section.	To determine PMR among twins born at term in relation to mode of delivery.	PMR (2^nd ^twin vs. 1^st^): OR = 5.00 (95% CI: 2.00–14.70) [6 vs. 30 deaths in first vs. second twins, respectively]. PMR (either twin): OR = 0.26 (95% CI: 0.03–1.03) **[NS]**. [2/1472 (0.14%) vs. 34/6601 (0.52%) deliveries in either twin by planned Caesarean section vs. other means, respectively; P = 0.05]. No association of birth order and PMR among those delivered by planned Caesarean section.

Smith et al. 2007 [148]	UK (England, Northern Ireland and Wales). Retrospective cohort study. N = 1377 twin pregnancies with one twin dying perinatally (excluding malformations) and one surviving, 1994–2003.	To assess PMR based on birth order in twin pregnancies.	Birth order and the risk of death overall: OR = 1.0 (95% CI: 0.9–1.1) for the second twin **[NS]**. However, there was a highly significant interaction with gestational age (P < 0.001). PMR among 2nd twins born at term: OR = 2.3 (95% CI: 1.7–3.2, P < 0.001). Higher risk for vaginal birth (OR = 4.1, 95% CI: 1.8 to 9.5) compared with Caesarean section (OR = 1.8, 95% CI: 0.9 to 3.6); P = 0.10. PMR among 2^nd ^twins at term associated with intrapartum anoxia or trauma (OR = 3.4, 95% CI: 2.2 – 5.3).

***VBAC vs. repeat Caesarean section***			

Bahtiyar et al. 2006 [149]	USA. Perinatal mortality data (1995 to 1997). Cross-sectional study. Deliveries of singleton term pregnancies (N = 11,061,599) in women 15–44 years collected 1995–97. Caesarean delivery rate was 19.6%.	Compared the impact on SBR among pregnant women with a prior Caesarean delivery (intervention) vs. women with no prior Caesarean delivery (control).	Crude fetal death (miscarriage+SB): 1.3/1000 vs. 1.5/1000 births in intervention vs. control groups, respectively. Adjusted fetal death (miscarriage+SB): 0.4/1000 vs. 0.6/1000 births in intervention vs. control groups, respectively. *Subset of women with only 1 prior delivery:* Fetal death (miscarriage+SB): RR = 0.90 (95% CI: 0.76–1.06) **[NS]**. [0.7/1000 vs. 0.8/1000 births in intervention vs. control groups, respectively].

Kumar et al. 1996 [42]	Western Australia. Retrospective study. Women (N = 79) with prior Caesarean section. N = 33 (41.8%) women agreed to a trial of vaginal birth. N = 29 women had labour induced and 26 (89.7%) of them had a successful vaginal delivery.	To assess the PMR in women attempting vaginal birth after Caesarean section (VBAC).	PMR: 0/79. Vaginal delivery rate: 87.9% in women undergoing a trial of vaginal birth. Caesarean section for fetal distress: 4/33 (12.1%). Caesarean section rate: fell from 32.2% to 11% in hospital during the study period.

Meehan et al. 1989 [43]	Ireland (Galway). Regional Hospital. Retrospective analysis. N = 27,072 babies born 1972–1982. N = 1498 patients with prior Caesarean section, including N = 654 (44%) with repeat elective Caesarean section and N = 844 (56%) with VBAC.	Compared the impact on PMR among women with prior Caesarean section according to the mode of delivery: elective Caesarean section, VBAC, and emergency Caesarean section).	PMR: 30.3/1000 (N = 46) vs. 22.5/1000 in all women with prior Caesarean section vs. overall hospital population, respectively. PMR: 10.6/1000 vs. 90.3/1000 in those delivered by elective Caesarean section vs. those by emergency Caesarean section (statistically significant) Successful vaginal delivery occurred in 702 (83%) patients and 142 (17%) had emergency repeat operations. Corrected PMR was twice as high in the trial of scar group.

Mock et al. 1991 [45]	West Africa. Rural hospital based. Descriptive study. Women (N = 220) with prior Caesarean section delivering 1987–1990. N = 169 patients given a trial of labour, of whom vaginal delivery was achieved in 111 (66%).	Compared the impact on maternal and fetal outcome between women given a trial of labour and those given elective repeat Caesarean section.	PMR:** [NS]**

Nyirjesy et al. 1992 [44]	Northeastern Zaire. Rural referral hospital. Descriptive study. Women (N = 33) with previous Caesarean given trial of labour in 1989–1990, of which 22 (67%) had successful vaginal deliveries.	Assessed the PMR in women given a trial of labour (study group) vs. the overall rate for the institution (controls).	PMR: 60.1/1000 (study group) **[NS] **compared to controls.

van Roosmalen 1991 [46]	Tanzania. 2 rural hospitals. Observational study. N = 134 women with a history of previous Caesarean section, of which N = 87 had a vaginal delivery after a trial of labour.	Compared PMR in women with a previous Caesarean birth in relation to the indication of the previous operation, a history of previous vaginal delivery and the number of previous operations.	PMR: 9/64 (14%) vs. 4/45 (9%) vs. 0/25 (0%) where the indication for previous Caesarean was CPD vs. nonrecurrent vs. unknown, respectively **[NS]**. PMR: 3/43 (7%) vs. 10/91 (11%) in women without previous vaginal birth vs. with previous vaginal respectively **[NS]**. PMR: 10/114 (9%) vs. 3/20 (14%) in women with one previous Caesarean vs. more than one Caesarean, respectively **[NS]**.

#### Availability and practice of Caesarean section

In low-/middle-income countries, prolonged and/or obstructed labour complicated by fetal asphyxia, fetal or placental infection, and birth trauma is a major cause of stillbirth, often arising from a small maternal pelvis due to childhood malnutrition, which subsequently leads to cephalopelvic disproportion during delivery [33]. Timely delivery, often by Caesarean section or instrumental vaginal delivery, can reduce associated intrapartum stillbirth, and is largely credited for the relatively low rates of intrapartum stillbirth in high-income countries [3].

**Figure 2:**
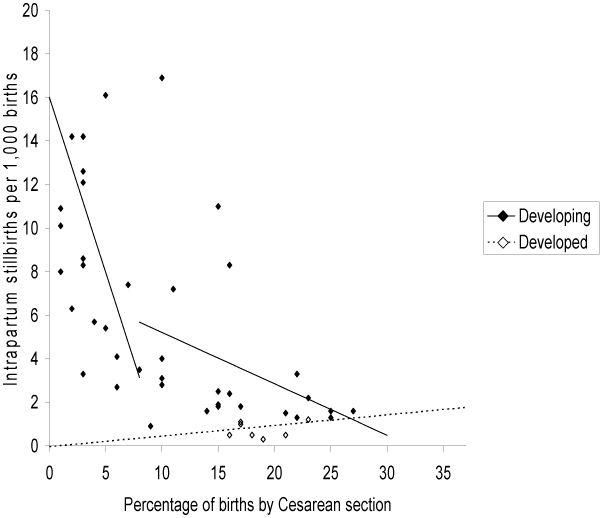
**Source: Goldenberg et al. 2007**.

**Figure 3:**
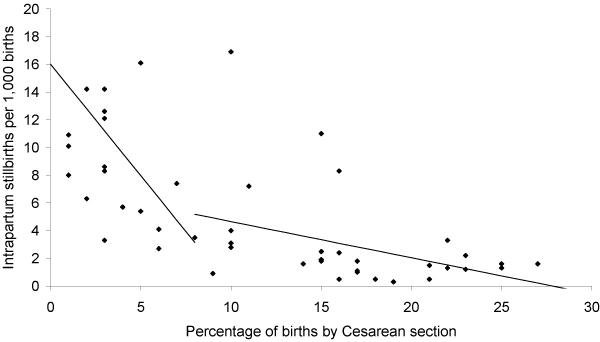
**Relationship between Cesarean sections and intrapartum stillbirths**. Source: Goldenberg et al. 2007.

**Figure 4:**
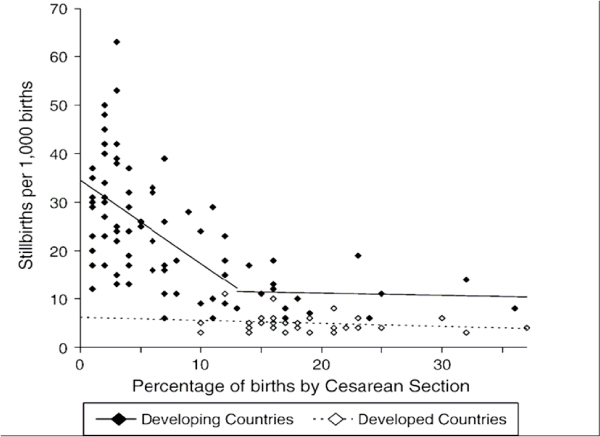
**Source: McClure et al. 2007**.

Using regression analysis of data from WHO and other sources to examine the association between stillbirth rates and obstetric care, Goldenberg et al. [3] and McClure et al. [21] observed that Caesarean section availability in low-/middle-income countries was associated with reductions in intrapartum stillbirths (Figures 2, 3 and 4). They [3] reported that intrapartum stillbirths dropped by 1.61 per 1000 births for every one percentage point increase in Caesarean section from 0 to 8 percent. Thereafter, they observed a small, non-significant increase in intrapartum stillbirths for each percent increase in Caesarean section. Intrapartum stillbirth rates correlated more closely with the measures of obstetric care in the regression analysis than did antepartum stillbirth rates, corroborating the theory that obstetric care availability and quality improvements will impact intrapartum stillbirth incidence more than antepartum stillbirth incidence. There was no relationship between Caesarean section and intrapartum stillbirths in high-income countries, which all had rates of Caesarean section of 15 percent or more. The WHO recommends Caesarean section rates of 10 to 15 percent, although they also note that 'countries with some of the lowest perinatal mortality rates in the world have a Caesarean rate of less than 10 percent,' and that perinatal mortality declines are steep until Caesarean section rates reach approximately 8 percent of deliveries, after which point the relationship becomes less clear [34]

Using data from the early 1980s in Australia, Alessandri et al. [35] performed a case-control study of intrapartum stillbirths, and could not identify any specific antenatal risk factors that predicted these demises. However, they did suggest that because the odds of emergency Caesarean section were significantly higher among stillbirth cases than live born controls, prompt Caesarean section might reduce intrapartum stillbirths, the risk of which was strongly associated with placental abruption or other placental problems, fetal distress, umbilical cord prolapse, and vaginal breech delivery. Other studies have confirmed that in many high-income country settings, liberal policies of Caesarean section are associated with positive effects on fetal survival, even when performed well before term [36-39].

While Caesarean section can be a life-saving intervention for both mother and child, its liberal use exposes some proportion of mothers and babies who do not need the procedure to unnecessary risks of morbidity. Additionally, in some low-/middle-income countries, particularly in low-resource areas, there is some evidence that the practice of Caesarean section may be associated with an *increased *risk of perinatal mortality [40,41], suggesting that Caesarean section may be performed too late or by inadequately skilled practitioners in these settings. Additionally, the elevated risk of uterine rupture in subsequent pregnancies after a Caesarean section should be a consideration in providing the procedure in remote areas. In addition to the risk of uterine rupture, the data are conflicting about whether vaginal labour after previous Caesarean is associated with increased risk of perinatal mortality [42-46]. Because of the known risk of uterine rupture, particularly among women whose prior Caesarean incision was classical rather than lower-segment, it is recommended that women who have had prior classical Caesarean section have immediate access to EmOC in subsequent pregnancies to reduce the risk of maternal mortality and stillbirth.

#### Quality of obstetric care

A number of studies associated suboptimal care, particularly inadequate, inappropriate, or delayed care of complications such as obvious fetal distress, placental abruption, breech presentation, twin pregnancy, or eclampsia, with increased perinatal mortality [47,48]. Several studies comparing hospitals or assessing health systems found that quality of care provided in facilities was directly associated with perinatal mortality [49-51,25]. Recent improvements in term stillbirth rates, especially intrapartum stillbirths, suggest that more rapid performance of Caesarean section in cases of placental abruption has resulted in a lower incidence of abruption-associated stillbirth [52]. In Virginia, USA, Cruikshank and Linyear [53] observed that intrapartum stillbirths associated with abruption were characterised by a fairly long delay between the appearance of fetal compromise and delivery, suggesting that timely Caesarean section could have prevented these deaths. In Finland, Korhonen and Kariniemi [54] found that having a surgical team in-hospital to perform Caesarean sections versus having the team on-call resulted in lower perinatal mortality (P = 0.05) and prevented all cases of fetal death and hypoxic ischemic encephalopathy (versus 3/41 in controls and 1/41 in controls, respectively).

Jehan et al. [55] recently reviewed stillbirths in rural Sindh Province, Pakistan, and found that a large proportion of these stillbirths occurred in facility settings with ostensibly skilled care providers. Because the rate of fetal mortality in labour seems to be reduced with adequate care, Kiely et al [56] have proposed using fetal deaths in labour as an epidemiologic measure of the quality of obstetric care. They found that delivery in a level 1 (primary care) hospital was associated with a 60% increase in intrapartum stillbirth compared to a level 3 facility (equipped for perinatal intensive care). Similarly, a retrospective analysis [57] found that rural residence was associated with elevated stillbirth rates (OR = 1.20; 95% CI: 1.09–1.32, P < 0.001) and neonatal death in hospital (adjusted OR = 1.26; 95% CI: 1.07–1.48) compared to urban residence, which granted women better access to higher-level emergency obstetric care.

There is some limited evidence that Caesarean section capability is not the only component of EmOC with the potential to reduce perinatal mortality. Advances in labour management and perinatal care have also contributed to significant declines in perinatal mortality. Steyn et al [58] observed that over a 20-year period in South Africa, the Caesarean section rate remained constant, but perinatal mortality decreased by 50 percent. In a remote area of the US in a high-risk Native American obstetric population, Leeman et al [30] documented a Caesarean section rate one-third of the national average without any adverse impact on perinatal mortality rate (PMR), which was the same as the national average. Even in remote areas without Caesarean section capability, intrapartum stillbirths and perinatal deaths can be significantly reduced with improved quality of obstetric and perinatal care alone. Grzybowski [59] undertook a prospective study to determine whether a small, isolated hospital without Caesarean section capability and which handled fewer than 50 births annually could provide safe obstetric and perinatal care. Over the 5-year study period, there were 6 perinatal deaths, for a perinatal mortality rate of 20.8 per 1000 (95% CI: 4.4–37.2 per 1000); however, the wide confidence intervals indicate the study was underpowered to measure perinatal mortality, and the perinatal mortality rate was still more than double that of many high-income countries, including the US [60].

#### Conclusion

While varied in design and lacking the rigor of RCTs, evidence from available, largely observational studies, taken together, indicates that availability of facilities capable of providing EmOC with trained care providers who are able to undertake safe and timely Caesarean section for appropriate indications is critical for reducing the risk of intrapartum stillbirths. It is not possible, however, based on the available data to ascertain the relative contribution of various components of EmOC to the mortality reductions observed. The need for Caesarean section capacity in rural settings is apparent from the data. The risk of uterine rupture and placental invasion of the uterine scar, and the higher rate of stillbirth in subsequent pregnancies after Caesarean section [61] suggests that liberal Caesarean section policies are unjustifiable, especially where access to emergency obstetric care is limited or home birth is common. Medically unnecessary Caesarean sections would place a large group of women at risk of uterine rupture if they do not or cannot access EmOC in subsequent pregnancies. A Caesarean section rate of 10–15% as recommended by WHO appears appropriate, particularly in resource-constrained settings; it is paramount that the procedure is performed for indications of fetal distress or other standard indications, rather than for the convenience of the provider or on maternal request. Additionally, several studies reported significant improvement in perinatal mortality rates in facilities without Caesarean section capability, and others demonstrated that Caesarean section provided too late or in too remote a setting increased perinatal mortality, suggesting that quality of obstetric care, rather than mere availability of Caesarean section, is key in preventing stillbirths and perinatal deaths. Despite the obvious lack of RCTs, the evidence in support of increased emergency Caesarean section availability to reduce stillbirths is strong (Grade B); EmOC is needed in all low-/middle-income country health systems.

### Induction of labour (versus expectant management)

#### Background

Induction of labour, a common intervention in obstetric practice, is indicated when it is determined that the fetus or mother will more likely have a healthy outcome than if birth is delayed. While many studies have compared different regimens and administration techniques of drugs to ripen the cervix and induce labour, few have evaluated the outcomes associated with induction versus expectant management of spontaneous labour for different indications [62]. Induction is frequently practised at term or post-term, or in cases in which the fetus is suspected to be macrosomic and therefore likely to require complicated operative delivery [63]. Additional common indications for induction of labour include pre-eclampsia, cases of premature rupture of membranes (PROM) where labour does not quickly become spontaneously established thereafter, and twin pregnancy. The process of induction of labour should only be considered when vaginal delivery is felt to be the appropriate route of delivery. Many different agents and methods can be used to induce and augment labour, including drugs such as oxytocin or prostaglandins, and physical interventions including sweeping the membranes or early amniotomy. Little is known about the impact of induction for different indications on stillbirth and perinatal outcomes.

#### Literature-based evidence

Our literature search identified 5 Cochrane reviews and 3 other interventional/observational studies (Table 4).

**Table 4:** Impact of elective induction of labour versus expectant management on stillbirths and perinatal mortality

**Source**	**Location and Type of Study**	**Intervention**	**Stillbirths/Perinatal Outcomes**
***Reviews and meta-analyses***			

Dare et al. 2006 [67]	Canada, Scotland, Netherlands, Israel, other countries. Meta-analysis (Cochrane). 5 RCTs included (N = 5870 participants).	To assess the effects of planned early birth (intervention) vs. expectant management (controls) for women with term pre-labour rupture of membranes on fetal, infant and maternal wellbeing.	Fetal death (miscarriage + SB)/PMR: OR = 0.46 (95% CI: 0.13–1.66) **[NS]**. [3/2946 vs. 7/2924 in intervention and control groups, respectively].

Dodd et al. 2003 [70]	Japan (Tokyo). Cochrane review. 1 RCT included (N = 72 participants).	To assess a policy of elective delivery from 37 weeks' gestation (intervention) vs. an expectant approach (controls) for women with an otherwise uncomplicated twin pregnancy.	PMR: RR not estimable. [0/34 vs. 0/38 in intervention and control groups, respectively].

Gülmezoglu et al. 2006 [64]	Thailand, USA, Turkey, Norway, Canada, UK, India, Finland, China. Meta-analysis (Cochrane). 12 RCTs included (N = 5939 women).	To assess the impact of a policy of labour induction at term or post-term (intervention) vs. awaiting spontaneous labour or later induction of labour (controls).	SBR: RR = 0.28 (95% CI: 0.05–1.67) **[NS]**. [0/2986 vs. 4/2953 in intervention and control groups, respectively]. PMR: RR = 0.30 (95% CI: 0.09–0.99). [1/2986 vs. 9/2953 in intervention and control groups, respectively].

Irion et al. 1998 [63]	USA, unknown. Meta-analysis (Cochrane). 2 RCTs included (N = 99 women).	To assess the effects of a policy of labour induction (intervention) vs. expectant management (controls) for suspected fetal macrosomia on method of delivery and maternal or perinatal morbidity.	PMR: RR not estimable. [0/49 vs. 0/50 in intervention and control groups, respectively].

Boulvain et al. 2001 [65]	USA. Cochrane review. 1 RCT included (N = 200 women).	To assess the effect of a policy of elective delivery (intervention) vs. expectant management (controls) in term diabetic pregnant women, on maternal and perinatal mortality and morbidity.	PMR: RR not estimable. [0/100 vs. 0/100 in intervention and control groups, respectively].

***Intervention studies***			

Chattopadhyay et al. 1986 [71]	Saudi Arabia. Prospective, controlled study. Grand multiparae (N = 300) between 38 and 42 weeks' gestation (N = 150 intervention group, N = 150 controls).	To compare the impact on labour characteristics and outcome in women where labour was electively induced by intracervical prostaglandin E2 tablets (intervention) vs. women who went into labour spontaneously.	SBR: 0/150 vs. 4/150 in intervention and control groups, respectively. Mean duration of the active phase of labour: 2.1 +/- 0.79 h vs. 2.8 +/- 0.47 h vs. 4.7 +/- 2.2 h in women who delivered on the first day of induction vs. on the second day vs. the controls. Similarly, the mean duration of the second and third stage was longer in the controls.

da Graca Krupa et al. 2005 [69]	Brazil (Campinas City). Public university hospital. RCT. N = 150 pregnancies, half of them allocated to each group.	To compare the effectiveness of immediate induction of labour with vaginal misoprostol (intervention) vs. expectant management for 24 hours followed by oxytocin induction (controls) in women with premature rupture of membranes at term (term PROM).	PMR: 0/75 in each group.

***Observational studies***			

Duff et al. 2000 [150]	Ireland. Northern Ireland Maternity System (NIMATS). Retrospective comparative study. N = 3262 women who delivered during 1994 – 96 (N = 1008 intervention group, N = 2254 controls).	Compared the impact on Caesarean section rates and Apgar scores in women who had labour induced (intervention) vs. those in whom the labour commenced spontaneously (controls).	Caesarean section rate: 12.2% vs. 7.06% in intervention and control groups, respectively **[NS] **(Chi sq = 4.39, p <= 0.2). 1 minute Apgar score: 7.78 vs. 7.9 in intervention and control groups, respectively, t = 2.9, P <= 0.01. 5 minute Apgar score: 8.99 vs. 9.05 in intervention and control groups, respectively, t = 2.42, P <= 0.02).

##### Post-term pregnancy/prevention of obstructed labour due to cephalo-pelvic disproportion

Considering the merits of induction in post-term pregnancies, Gülmezoglu et al. [64] reported a non-significant reduction in stillbirth risk (RR = 0.28, 95% CI: 0.05–1.67, 12 trials, N = 5939 women), but a statistically significant reduction in perinatal mortality (RR = 0.30, 95% CI: 0.09–0.99, 12 trials, N = 5939 women), in induced versus expectantly managed groups ***[LOE: 1+] ***(Additional file 3).

Irion et al. [63] (Additional file 4) reviewed three trials involving 372 non-diabetic women who underwent induction of labour for suspected fetal macrosomia. Compared to expectant management, induction of labour for suspected macrosomia did not reduce risk of Caesarean section (RR = 0.96, 95% CI: 0.67–1.38) or instrumental delivery (RR = 1.02, 95% CI: 0.60–1.74) ***[LOE: 1++]. ***Boulvain et al. [65] (Additional file 5) conducted a review of elective delivery, either by induction of labour or by elective Caesarean section, compared to expectant management, and included one trial [66] which compared a policy of active induction of labour at 38 weeks to expectant management until 42 weeks. The risk of Caesarean section was not statistically different between groups (RR = 0.81, 95% CI: 0.52–1.26), but the risk of fetal macrosomia was reduced in the active induction group (RR = 0.56, 95% CI: 0.32–0.98). No other perinatal morbidity was reported ***[LOE: 1-].***

##### Premature rupture of membranes (PROM)

Dare et al. [67] conducted a Cochrane review to assess the effects of induction of labour versus expectant management among women with premature rupture of membranes (PROM) at term (12 RCTs, N = 6814 women) (Additional file 6). They found that planned management generally involved induction with oxytocin or prostaglandin; one trial used homoeopathic caulophyllum. Significantly fewer women in the induced compared with the expectant management groups developed chorioamnionitis (RR = 0.74, 95% CI: 0.56–0.97; 9 trials, 6611 women) or endometritis (RR = 0.30, 95% CI: 0.12 to 0.74; 4 trials, 445 women). There was a non-significant trend toward lower perinatal mortality (OR [fixed]: 0.46, 95% CI: 0.13–1.66) and neonatal infection incidence (RR = 0.83, 95% CI: 0.61–1.12; 9 trials, 6406 infants) in the induced versus the expectantly managed groups. There was no difference between the two groups in Caesarean section rates (12 trials, N = 6814 women, RR = 0.94, 95% CI: 0.82–1.08) or operative vaginal birth (7 trials, N = 5511 women, RR = 0.98, 95% CI: 0.84–1.16) ***[LOE: 1+]. ***Another Cochrane review on induction of labour for pre-term PROM (prior to 37 weeks of gestation) is in progress [68]. An intervention study from Brazil [69] not included in the above Cochrane review reported no difference in fetal deaths among a group of women with PROM at term (N = 150) given vaginal misoprostol vs. those managed expectantly for 24 hours, then given intravenous oxytocin ***[LOE: 1+]***.

##### Multiple pregnancy

Dodd et al. [70] investigated the optimal timing of elective induction of labour after 37 weeks in twin pregnancy (N = 72 women) and reported no statistically significant differences between a group electively induced at 37 weeks versus an expectantly managed group; the study was underpowered to detect true differences in perinatal mortality rates ***[LOE: 1-] ***(Additional file 7).

##### Grand multiparity

Several other intervention and observational studies not included in the Cochrane reviews addressed the impact of induction of labour on perinatal outcomes. In Saudi Arabia among a group of grand multiparas at term, Chattopadhayay et al. [71] reported a lower stillbirth rate in a group receiving intracervical prostaglandin E2 tablets compared to a group in which labour was managed expectantly (SBR: 0/150 vs. 4/150 in prostaglandin versus expectantly managed groups, respectively; no statistical significance data given).

#### New meta-analysis

**Figure 5:**
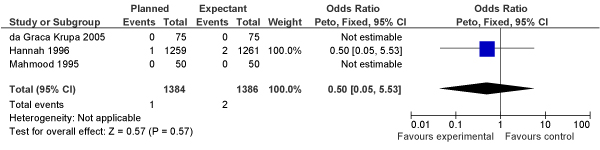
**Results of new meta-analysis of impact of planned versus expectant management for pre-labour rupture of membranes at term (by use of prostaglandin) on fetal/perinatal mortality**.

We conducted a meta-analysis of trials (3 RCTs, N = 1770 women) of planned induction of labour using prostaglandins versus expectant management in women with PROM. No significant decrease in perinatal mortality was found (OR = 0.50, 95% CI: 0.05–5.53) in the planned induction versus expectantly managed groups, respectively (Figure 5).

#### Conclusion

The evidence from this review indicates that induction of labour does not impact the likelihood of delivery by Caesarean section. Two trials, however, showed a significant reduction in the risk of Caesarean section from a policy of routine induction of labour among low-risk women with post-term pregnancy [72,73], though performance bias may have skewed the results in the Hannah trial.

Induction of labour for suspected fetal macrosomia in non-diabetic women has not been shown to alter the risk of maternal or neonatal morbidity, but the power of the included studies to show a difference in rare events is limited. Large trials are ongoing to address this question. Induction of labour appears to be an appropriate intervention in post-term pregnancy at 41 completed weeks of gestation or later. The Cochrane review by Gülmezoglu et al. [64] comparing elective induction of labour with expectant management showed a statistically significant reduction in perinatal mortality and meconium aspiration syndrome. Although increasingly more commonplace, elective inductions are not advised before 39 weeks gestation given the potential, albeit low, for complications associated with prematurity. Even in cases of post-term pregnancy, 500 inductions may be required to prevent one perinatal death. The reduction in stillbirth incidence noted in the Gülmezoglu review was not statistically significant, likely because the number of stillbirths was too small.

Larger RCTs are needed to address the impact of induction of labour on stillbirth and perinatal mortality for the following indications: suspected macrosomia in non-diabetic mothers, multiple pregnancy, and mild pre-eclampsia.

### Drugs for cervical ripening and induction of labour

#### Background

There are a variety of drugs, including oxytocin and a number of prostaglandins and prostaglandin analogues, and many different administration methods including intracervical, vaginal, oral, and intravenous (IV) routes, for the induction of labour. Drugs to induce labour can have adverse side effects, fail to induce labour, or cause dysfunctional labour or hyperstimulation of the uterus leading to fetal distress and Caesarean section. Fetal and possibly maternal death is possible if Caesarean section is not available or delayed when these drugs are used. The state of cervical ripening and favourability for induction should be assessed before a regimen is selected, as oxytocin induction, in particular, often fails unless the cervix is ripe. In women with unfavourable cervical ripening, different prostaglandin drugs, including prostaglandin F2-alpha, prostaglandin E2 (dinoprostone), and prostaglandin E1 (misoprostol) promote cervical ripening and initiation of labour [74]. Misoprostol is the only prostaglandin analogue that is effective in inducing labour without gastrointestinal side effects when given as an oral preparation. It is inexpensive and stable at room temperature, making it an easily administered intervention appealing for use in low-resource settings, though the risk of uterine hyperstimulation at high doses may produce increased risk of maternal or perinatal death [75]. Insertion of a Foley catheter has also been shown to be as effective as prostaglandin E2 for stimulating preinduction cervical ripening, potentially providing an effective, safe, non-pharmacological mechanical method of preinduction cervical ripening [76-78].

Given the use of induction for certain indications including post-term pregnancy, pre-eclampsia, and PROM in which the fetus is at higher risk of perinatal death, as well as the potential for drugs used to induce labour to cause fetal distress, an assessment of the evidence of impact of specific drugs available for induction of labour on perinatal outcomes is warranted.

#### Literature-based evidence

Our literature search identified 8 Cochrane reviews and 11 other studies (Table 5) assessing the impact of different drugs for cervical ripening and/or induction of labour. The trials identified are grouped below by the drugs being compared and the route of administration.

**Table 5:** Impact of drugs for cervical ripening and induction of labour on stillbirth and perinatal outcomes

**Source**	**Location and Type of Study**	**Intervention**	**Stillbirths/Perinatal Outcomes**
***Reviews and meta-analyses***			

Kelly et al. 2003 [90]	UK, Austria, New Zealand, Singapore, USA, Pakistan, Canada. Meta-analysis (Cochrane). 8 RCTs included (N = 3648 women).	To compare the effects of vaginal prostaglandins E2 (all regimens) for third trimester cervical ripening or induction of labour (intervention) vs. placebo/no treatment (controls).	PMR: RR = 0.56 (95% CI: 0.14–2.22) **[NS]**. [2/1833 vs. 4/1815 in the intervention and control groups, respectively].

Boulvain et al. 2008 [62]	USA, Europe, Africa, UK, Italy. Meta-analysis (Cochrane). 4 RCTs included (N = 1081 women).	Compared the impact on perinatal mortality of intracervical prostaglandin (prostaglandin E2) (intervention) vs. placebo/no treatment (controls) for third trimester cervical ripening and induction of labour.	PMR: RR = 0.20 (95% CI: 0.01–4.05) **[NS]**. [0/587 vs. 2/494 in intervention and control groups, respectively].

Hutton et al. 2001 [92]	2 RCTs. Zimbabwe, Australia. Cochrane review. 2 RCTs included (N = 25 women).	To assess the effects of extra-amniotic prostaglandin (PGF2 alpha) (intervention) for third trimester cervical ripening or induction of labour vs. extra amniotic placebo gel (controls).	PMR: RR = 2.06 (95% CI: 0.09–46.11) **[NS]**. [1/15 vs. 0/10 in intervention and control groups, respectively].

Hofmeyr et al. 2003 [79]	Chile, Zimbabwe, USA, Canada, Jamaica, Malaysia. Meta-analysis (Cochrane). 7 RCTs included (N = 268 women).	To assess the effects of vaginal misoprostol for third trimester cervical ripening or induction of labour (intervention) vs. vaginal prostaglandin (controls).	PMR: RR = 2.85 (95% CI: 0.12–68.95) **[NS]**. [1/136 vs. 0/132 in intervention and control groups, respectively].

Neilson 2000 [94]	France, Sweden. Meta-analysis (Cochrane). 2 RCTs included (N = 68 women).	To assess the effects of mifepristone (all doses) for third trimester cervical ripening or induction of labour (intervention) vs. placebo/no treatment (controls).	PMR: RR not estimable. [0/40 vs. 0/28 in intervention and control groups, respectively].

French 2001 [89]	India, Denmark. Cochrane review. 2 RCTs included (N = 35 women).	To assess the effects of oral prostaglandin E2 for third trimester induction of labour (intervention) vs. intravenous oxytocin (controls) on perinatal mortality.	PMR: RR not estimable. [0/15 vs. 0/20 in intervention and control groups, respectively].

Luckas et al. 2000 [93]	USA, UK, Denmark, Belgium and Netherlands. Meta-analysis (Cochrane). 11 RCTs included (N = 990 women).	To assess the effects of intravenous prostaglandin for third trimester cervical ripening or induction of labour (intervention) vs. IV oxytocin (controls).	PMR: RR = 3.59 (95% CI: 0.60–21.53) **[NS]**. [4/499 vs. 0/491 in intervention and control groups, respectively].

Alfirevic 2006 [75]	Hong Kong, Switzerland, South Africa, UK, Spain, Canada, USA. Meta-analysis (Cochrane). 17 RCTs included (N = 1508 women).	To assess the effectiveness and safety of oral misoprostol used for labour induction in women with a viable fetus in the third trimester of pregnancy (intervention) vs. vaginal prostaglandin (controls).	PMR: RR = 0.60 (95% CI: 0.08–4.50) **[NS]**. [data from 4 RCTs; 1/756 vs. 2/752 in intervention and control groups, respectively].

***Intervention studies***			

Elhassan et al. 2005 [85]	Sudan. Non-blinded RCT. N = 140 patients (N = 70 intervention, N = 70 controls).	Assessed the impact of vaginal misoprostol, 50 μg, six hourly until initiation of labour or maximum of 4 doses (intervention) vs. IV infusion of oxytocin at 2 mU/min, doubled at 30-minute intervals until the appropriate contraction pattern obtained or dose increased to a maximum of 20 mU/minute and maintained as such (controls).	Neonatal outcomes (birth weight, Apgar score and SBR): **[NS]**

Garry et al. 2003 [80]	USA. RCT. Singleton gestations (N = 200) with an indication for cervical ripening and induction of labour.	Compared the impact of 50 μg of vaginal misoprostol every 3 h (intervention) vs. a 10-mg prostaglandin E2 vaginal insert every 12 h for a maximum of 24 h (controls).	Neonatal outcomes: **[NS]** Vaginal delivery <12 hr: 44% vs. 12% in misoprostol vs. prostaglandin E2 group, respectively (P < 0.0001) Vaginal delivery <24 hr: 68% vs. 38 in misoprostol vs. prostaglandin E2 group, respectively (P < 0.001). Caesarean delivery for fetal distress: 71.4% (20/28) vs. 40% (14/35) in misoprostol group vs. prostaglandin E2 group (P = 0.03).

Jindal et al. 2007. [87]	India. Quasi-RCT. N = 100 women (N = 50 intervention group, N = 50 controls).	Compared the impact of 50 μg of vaginal misoprostol 4 hourly for a maximum of six doses (intervention) vs. transcervical Foley catheter with simultaneous intravenous oxytocin (controls).	SBR: 1/50 vs. 0/50 in intervention and control groups, respectively.

Lokugamage et al. 2003 [81]	UK. Hospital based. RCT. N = 191 patients.	Compared the impact of 50 μg vaginal misoprostol initially then a further identical dose 6 hrs later (intervention) vs. 2 mg vaginal prostaglandin E2 initially followed by 1 mg 6 hrs later, over a period of 24 hrs (controls). All participants not in labour after 24 hrs received prostaglandin E2 alone as per hospital protocol.	Neonatal outcome: **[NS]** Induction-to-delivery interval: 1047 vs. 1355 min (P = 0.01) in intervention and control groups, respectively. Delivery <12 hrs: 35.4% vs. 18.9%, (P = 0.02) in intervention and control groups, respectively. Delivery <24 hrs: 83.3% vs. 63.3%, (P = 0.01) in intervention and control groups, respectively. Oxytocin augmentation: **[NS] **(P = 0.47), Tachysystole: **[NS] **(P = 0.32) and Hyperstimulation syndrome: **[NS] **(P = 0.82).

Majoko et al. 2002 [151]	Zimbabwe. RCT. N = 152 women admitted for induction of labour (N = 76 in each group).	Compared the impact of vaginal misoprostol (intervention) versus extra-amniotic prostaglandin F2α gel (controls).	SBR: 1/76 in each group due to asphyxia (both mothers induced for pre-eclampsia; deaths resulting from inadequate response to fetal distress).

Meyer et al. 2005 [86]	USA. RCT. N = 84 patients.	Compared the impact of 0.25μg misoprostol vaginally (intervention) vs. 0.5 mg prostaglandin E2 gel intracervically (controls), the evening before oxytocin induction.	Neonatal outcome: **[NS]** Caesarean rate: 9/42 vs. 8/42 in intervention and control groups, respectively **[NS]**

Papanikolaou et al. 2004 [84]	Greece. RCT. Nulliparous pregnant women (N = 163) with an unfavorable cervix and > 285 days of gestation (N = 80 intervention group, N = 83 controls).	Compared the efficacy of 50 μg vaginal misoprostol (intervention) versus 3 mg prostaglandin E2 (controls), administered every 9 hrs for a maximum of three doses for elective induction of labour.	SBR or PMR: 0/80 vs. 1/83 (1.2%) in intervention and control groups, respectively **[NS]**.

Rowlands et al. 2001 [83]	Australia. RCT. N = 126 women recruited to the study (N = 63 in each group).	Compared the effect on neonatal outcomes of vaginal prostaglandin E2 (group 1) vs. vaginal misoprostol (controls) for cervical priming prior to induction of labour.	Neonatal outcome (low cord pH, Apgar score at delivery or admission to the neonatal special care nursery): **[NS]**

Sahin et al. 2002 [88]	Turkey. RCT. N = 100 pre-eclamptic women with a modified Bishop score of = 4 (N = 50 in each group).	Compared the impact of 50 μg vaginal misoprostol 4 times at 4 hour intervals (intervention) vs. oxytocin infusion for induction of labour starting from 1 mIU/per min, increasing it every 30 min with 2 mIU/min increments up to maximum of 30 mIU/min (controls).	Intrapartum SB: 0/50 in both groups.

Sahraoui et al. 2005 [91]	Tunisia. RCT. All uncomplicated pregnancies that reached 41 weeks'gestation with a Bishop score of < or = 4.	Compared the impact on fetal outcomes of cervical prostaglandin E2 gel for cervical ripening (intervention) vs. control.	Caesarean rates: **[NS]** Rates of admission into the neonatal unit and fetal outcomes: **[NS]**

Van Gemud et al. 2004 [82]	The Netherlands. Labour wards of one university hospital and two teaching hospitals. RCT. Women (N = 681) with indication for labour induction at > or = 36 weeks of gestation, singleton pregnancy and no previous Caesarean section.	Compared the impact on pregnancy outcomes of misoprostol (25 mcg, hospital-prepared capsule) in the posterior vaginal fornix, every four hours, maximum three times daily (intervention) vs. prostaglandin E2 gel every four hours (controls). Oxytocin was administered if necessary	Neonatal deaths: (excluding malformations): 0 in both groups. Adverse neonatal outcome: 21% vs. 23% in intervention and control groups, respectively **[NS]**. Median induction-delivery interval: 25 vs. 19 h in intervention and control groups, respectively (P = 0.008). Caesarean rate: RR = 0.8 (95% CI: 0.6–1.04) **[NS]**. [16.1% vs. 21% in intervention and control groups, respectively]. Admission to NICU: RR = 0.7 (95% CI: 0.5–0.98). [19% vs. 26% in intervention and control groups, respectively].

##### Oral misoprostol versus placebo, other prostaglandins, or vaginal misoprostol

Alfirevic et al. [75] (Additional file 8) conducted a Cochrane systematic review of all trials (41 trials, N = 8606 women) comparing oral misoprostol with various other drugs for induction of labour. No studies comparing oral misoprostol to placebo reported perinatal outcomes. Comparing oral misoprostol with vaginal prostaglandin E2, there was no significant reduction in risk of perinatal mortality (5 trials, N = 2249, RR = 0.60, 95% CI: 0.08–4.50); risk of Caesarean section (9 trials, N = 2627 participants) reached statistical significance only in the subgroup with intact membranes (RR = 0.78, 95% CI: 0.66–0.94). Uterine hyperstimulation was more common after oral misoprostol (RR = 1.63, 95% CI: 1.09–2.44) although this was not associated with any adverse fetal events. Comparing oral misoprostol versus vaginal misoprostol preparations, the meta-analysis found no difference in perinatal outcomes (16 trials, N = 3645 participants) ***[LOE: 1++].***

##### Vaginal misoprostol versus placebo, other prostaglandins, or oxytocin

A Cochrane review by Hofmeyr et al. [79] (Additional file 9) evaluated 70 RCTs to determine the impact of vaginal misoprostol for cervical ripening or induction of labour. Compared to placebo, vaginal misoprostol was associated with increased success in achieving vaginal delivery within 24 hours (RR = 0.36, 95% CI: 0.19–0.68), but the risk of uterine hyperstimulation without fetal heart rate changes was increased (RR = 11.66, 95% CI: 2.78–49). Only one study reported perinatal mortality and uterine rupture as outcomes, risk ratios were not determined. The findings were similar when vaginal misoprostol was compared with vaginal prostaglandin E2, intracervical prostaglandin E2, and oxytocin, though none of these interventions had any differential impact on perinatal outcome. Many studies were small and reported no perinatal deaths in either group. Compared with vaginal or intracervical prostaglandin E2, oxytocin augmentation was less common and meconium-stained liquor more common with misoprostol ***[LOE: 1+].***

Our literature search identified additional RCTs and a cohort study not included in the above-mentioned Cochrane review comparing vaginal misoprostol with vaginal prostaglandin E2; all indicated no significant differences in neonatal outcomes, and none specifically reported stillbirth rates. RCTs by Garry et al [80]***[LOE: 1-] ***and Lokugamage et al. [81]***[LOE: 1-] ***compared vaginal misoprostol versus vaginal prostaglandin E2 inserts for cervical ripening and labour induction, finding no significant differences in perinatal outcomes. A similar RCT by van Gemund et al  [82] comparing vaginal misoprostol with prostaglandin E2 for induction of labour found similar rates of adverse neonatal outcome in both groups: 21% in the misoprostol and 23% in the prostaglandin E2 groups ***[LOE: 1+]. ***An RCT by Rowlands et al. [83] comparing vaginal misoprostol and vaginal prostaglandin E2 for cervical priming prior to the induction of labour found no differences between the groups in low cord pH, Apgar score at delivery, or admission to the neonatal special care nursery ***[LOE: 1-]. ***An RCT in Greece by Papanikolaou et al. [84] reported no statistically significant difference in perinatal mortality in babies born to women receiving vaginal misoprostol versus vaginal prostaglandin E2 (0/80 vs. 1/83 [1.2%], respectively) ***[LOE 1-]***.

Several other trials compared vaginal misoprostol to oxytocin and other prostaglandins administered via different administration routes. None reported any significant differences in impact on perinatal outcomes. An RCT in Sudan by Elhassan et al. [85] compared vaginal misoprostol to IV oxytocin and reported no difference in birth weight, Apgar score at birth, or stillbirth rates ***[LOE: 1-]***. Meyer et al [86] found no difference in impact of a single outpatient dose of vaginal misoprostol (versus intracervical prostaglandin E2 gel) on subsequent use of oxytocin for induction, short-term neonatal outcome or rates of Caesarean delivery ***[LOE: 1+]. ***A quasi-RCT in India [87] comparing vaginal misoprostol to Foley catheter for cervical dilatation plus IV oxytocin for induction of uterine contractions reported one stillbirth in the misoprostol group and none in the oxytocin plus Foley catheter group, but the sample size was too small to reach statistical significance ***[LOE: 2+]***. A similar RCT of vaginal misoprostol versus IV oxytocin in Turkey by Sahin et al. [88] reported no intrapartum stillbirths in either group (0/50 in each group) ***[LOE: 1+].***

##### Oral prostaglandins versus IV oxytocin

One Cochrane review by French et al. [89] (Additional file 10) compared oral prostaglandin E2 for third trimester induction of labour to IV oxytocin (2 RCTs, N = 35 women), reporting no perinatal deaths in either group (0/15 vs. 0/20 in prostaglandin E2 versus IV oxytocin groups, respectively).

##### Intracervical prostaglandins versus placebo

A Cochrane review by Boulvain et al. [62] (28 trials, N = 3764 women) comparing intracervical prostaglandins with placebo found a trend toward a lower PMR in the prostaglandin group but the result was not significant (RR [fixed] = 0.20, 95% CI: 0.01–4.05 [NS]) (Additional file 11). Prostaglandin E2 was associated with a decreased rate of labours lasting more than 24 hours versus placebo (4 trials, RR = 0.61, 95% CI: 0.47–0.79), but only a trend toward reduced risk of Caesarean section (RR = 0.88, 95% CI: 0.77–1.00). This decreased Caesarean section risk was statistically significant in the subgroup of women with intact membranes and unfavourable cervix (RR = 0.82; 95% CI: 0.68–0.98). The risk of hyperstimulation with fetal heart rate changes was not significantly increased (RR = 1.21; 95% CI: 0.72–2.05). However, the risk of hyperstimulation without fetal heart rate changes was significantly increased (RR = 1.59, 95% CI: 1.09–2.33) ***[LOE: 1+].***

##### Vaginal prostaglandins versus placebo

Kelly et al. [90] conducted a Cochrane review to determine the effects of vaginal prostaglandins E2 and F2α for cervical ripening or induction of labour in comparison with placebo/no treatment, and reported a non-significant reduction in PMR (RR [fixed] = 0.56, 95% CI: 0.14–2.22 [NS])*[****LOE: 1+] ***(Additional file 12). One RCT compared vaginal prostaglandin E2 versus placebo [91], reporting no difference in fetal or neonatal outcomes or rates of Caesarean section ***[LOE: 2-]***.

##### Extra-amniotic prostaglandins versus placebo

A Cochrane review by Hutton et al. [92] compared the impact of extra-amniotic PGF2 versus extra-amniotic placebo gel for third trimester cervical ripening or induction of labour, and found no significant impact on PMR (RR [fixed] = 2.06, 95% CI: 0.09–46.11 [NS])*[****LOE: 1++] ***(Additional file 13).

##### Intravenous prostaglandins versus intravenous oxytocin

A Cochrane review by Luckas et al. [93] determined the effects of IV prostaglandin versus IV oxytocin for third trimester cervical ripening or induction of labour, and reported a non-significant trend toward increased risk of perinatal mortality with IV prostaglandin (RR [fixed] = 3.59, 95% CI: 0.60–21.53 [NS]) ***[LOE: 1+] ***(Additional file 14).

##### Mifepristone versus placebo

A Cochrane analysis by Neilson [94] assessed the effectiveness of mifepristone, typically used to induce abortion or induce labour in cases of antepartum miscarriage or stillbirth, versus placebo in cervical softening and induction of labour (2 trials, N = 68 women). There were no perinatal deaths in either group (Additional file 15).

#### New meta-analysis

##### Vaginal misoprostol versus prostaglandin E2

**Figure 6:**
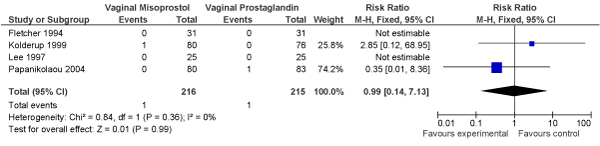
**Results of new meta-analysis (Fixed model) of impact of vaginal misoprostol vs. prostaglandin E2 for cervical ripening and induction of labour on perinatal mortality**.

**Figure 7:**
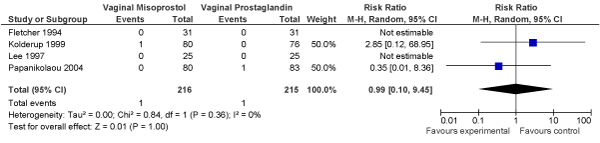
**Results of new meta-analysis (Random model) of impact of vaginal misoprostol vs. prostaglandin E2 for cervical ripening and induction of labour on perinatal mortality**.

We included 4 RCTs reporting perinatal death as outcome in a meta-analysis comparing vaginal misoprostol to prostaglandin E2 for inducing labour (N = 431 women; N = 216 misoprostol, N = 215 prostaglandin E2). We found no difference in perinatal death comparing misoprostol to prostaglandin E2 (RR [fixed] = 0.99, 95% CI: 0.14–7.13; RR [random] = 0.99, 95% CI: 0.10–9.45) (Figures 6 and 7).

#### Conclusion

Both vaginal and oral misoprostol are more effective than placebo and comparable to intravenous oxytocin or vaginal prostaglandin E2 in inducing labour at term [75,79]. Misoprostol at any dosage carries higher risk of meconium staining, but this was not associated with any adverse perinatal outcomes. Compared with vaginal prostaglandins, oral misprostol appears to reduce rates of Caesarean section [79] and vaginal misoprostol is associated with shorter labour, fewer side effects, and lower incidence of retained placenta, with no difference in perinatal mortality. However, there remain questions about the safety of both vaginal and oral misoprostol because of a relatively high rate of uterine hyperstimulation and the lack of appropriate dose ranging studies. While no increase in adverse fetal outcomes was reported in any of the misoprostol studies, the number of adverse fetal outcomes was too few to draw meaningful conclusions. There is no evidence to support the selective use of either oral or vaginal misoprostol on either perinatal mortality or stillbirths.

There was no statistically significant impact of any prostaglandin preparation on perinatal outcomes; it should be noted that most studies reviewed were small and the numbers of adverse outcomes too small to accurately measure differences in stillbirth or perinatal mortality rates between study arms. Vaginal prostaglandin E2, especially as vaginal tablets, appears to be an effective labour induction agent, improving the likelihood of vaginal delivery within 24 hours and reducing the need for augmentation with oxytocin, without increasing the risk of Caesarean section [90]. Vaginal prostaglandin E2 may reduce perinatal mortality, but the non-significant findings in the Cochrane review by Kelly et al indicate the need for further large RCTs. The intracervical mode of administration of prostaglandins is less used and less well evaluated than other modes of administration [62], and appears to be less effective in inducing labour than vaginal prostaglandin application, with no benefit for perinatal mortality. The data for perinatal mortality associated with extra-amniotic prostaglandins are extremely limited and require further trials [92].

### Planned Caesarean section for breech presentation

#### Background

Approximately 3–4% of term singleton pregnancies are complicated by breech presentation at term, and in most high-income countries, the majority of these births are handled via either planned or emergency Caesarean section. Whether planned caesarean section for breech presentation at term results in better perinatal outcomes is a contentious issue. An alternative to breech Caesarean section or breech vaginal delivery is external cephalic version, which has been shown to reduce numbers of breech births and breech Caesarean sections without impacting perinatal mortality [95,96]. Until recently the major evidence for Caesarean section for breech presentation has been derived from patient case studies and register studies lacking sufficient rigor on which to make policy or clinical management recommendations. Here, we review the evidence for impact of planned Caesarean section for breech presentation on perinatal mortality and stillbirth rates.

#### Literature-based evidence

Our literature search identified 1 Cochrane review and 3 other intervention/observational studies assessing the impact of planned Caesarean section for breech presentation on perinatal mortality outcomes (Table 6). The Cochrane review by Hofmeyr et al. [97] (Additional file 16) evaluated RCTs (3 trials, N = 2396 women), including the large Term Breech Trial [98], comparing planned Caesarean section with planned vaginal birth for singleton breech presentation at term. Perinatal or neonatal death (excluding fatal anomalies) or serious neonatal morbidity was reduced with planned Caesarean section compared to planned vaginal delivery [3/1166 (0.26%) vs. 14/1222 (1.15%), respectively; RR = 0.29, 95% CI: 0.10–0.86; 3 trials; N = 2388 women]. Compared with planned vaginal breech delivery, planned Caesarean section was associated with an increased risk of avoidable short-term maternal morbidity, including postpartum infection, haemorrhage, and anaemia (RR = 1.29, 95% CI: 1.03–1.61) ***[LOE: 1+].***

**Table 6:** Impact of planned Caesarean section for breech presentation on stillbirth and perinatal mortality

**Source**	**Location and Type of Study**	**Intervention**	**Stillbirths/Perinatal Outcomes**
***Reviews and meta-analyses***			

Hofmeyr et al. 2003 [97]	USA. Meta-analysis (Cochrane). 3 RCTs included (N = 2388 women).	To assess the effects of planned Caesarean section for singleton breech presentation at term (intervention) vs. planned vaginal delivery on measures of pregnancy outcome.	PMR/NMR (excluding fatal malformations): RR = 0.29 (95% CI: 0.10–0.86). [3/1166 vs. 14/1222 in intervention and control groups, respectively].

***Intervention studies***			

Irion et al. 1998 [99]	Switzerland (Geneva). University hospital. Cohort study. N = 705 consecutive singleton term breech presentations (N = 385 planned vaginal deliveries, N = 320 planned Caesarean sections).	Compared the impact on neonatal mortality of planned vaginal delivery vs. elective Caesarean section in term breech presentations.	NMR (all major malformations): RR = 3.33 (95% CI: 0.37–29.60). [1% vs. 0.3% in planned vaginal vs. Caesarean groups, respectively; P = 0.38].

Molkenboer et al. [100]	The Netherlands. 2 centres. Retrospective matched cohort study. N = 1119 deliveries between July 1998 and April 2000 (N = 373 breech between 37(+0) and 41(+6) weeks, N = 746 cephalic position).	Compared the impact on perinatal mortality in babies with breech presentation (exposed) vs. those with cephalic presentation (unexposed).	PMR: 0 in both groups. Planned Caesarean: 23.3% vs. 3.5% in breech vs. cephalic deliveries, respectively; P < 0.001. Emergency Caesarean: 29.2% vs. 8.8% in exposed vs. unexposed groups, respectively; P < 0.001.

Nalliah et al. 2009 [152]	Malaysia (Ipoh). Hospital based study. Retrospective analysis. N = 4886 breech presentations from 1992–2004, of which 3725 were evaluated.	Compared perinatal mortality among breech births delivered vaginally versus by Caesarean section.	PMR: Mode of delivery did not improve PMR in breech cases.

In Switzerland, a cohort study by Irion et al. [99] of planned vaginal versus planned Caesarean section for breech singleton presentation at term (N = 705 women) found a non-significant increased risk of neonatal mortality among the group delivering vaginally, but all deaths were attributable to major malformations (1% vs. 0.3% in planned vaginal vs. Caesarean section groups, RR = 3.33, 95% CI: 0.37–29.60 [NS]) ***[LOE: 2-]***.

In The Netherlands, Molkenboer et al. [100] conducted a 2-centre retrospective matched cohort study including all singleton breech deliveries from 1998–2000 of at least 37 and less than 42 weeks' gestation, excluding antepartum stillbirths, but no perinatal deaths occurred in either group ***[LOE: 2-]***.

#### Conclusion

The above evidence for planned caesarean section delivery for singleton breech presentation at term indicates that this intervention is associated with a statistically significant reduction in perinatal and neonatal mortality and morbidity, though it is associated with increased short-term maternal morbidity associated with recovery from a Caesarean section. The largest study in the Cochrane review, the Term Breech Trial, demonstrates strong evidence of benefit of planned caesarean section in reducing perinatal mortality, though it should be noted that no point estimates for impact on stillbirths were reported. Additionally, a number of methodological and analytical criticisms have been levied at the largest study in the Cochrane review (the Term Breech Trial, whose principal investigator co-authored the Cochrane review) including a lack of adherence to inclusion criteria, great variability in level of care between facilities and labours (including insufficient monitoring in some cases), and a lack of clinicians with expertise in vaginal breech delivery, Glezerman M: Five years to the term breech trial: the rise and fall of a randomised controlled trial. Am J Obstet Gynecol 2006, 194:20-25.    

Although planned Caesarean for breech presentation appears to be a promising intervention, in light of these criticisms and the downstream implications of recommending a policy of Caesarean section in low-resource settings, our recommendation for Caesarean section in term breech presentation carries several caveats. Given the risks associated with Caesarean section, a planned Caesarean section should only be considered after external cephalic version has been unsuccessful and sufficient time has been allowed for spontaneous version and cephalic birth to take place [95]. Caesarean section poses short-term risks of infection and haemorrhage, as well as long-term risks including uterine rupture (particularly where a classical incision is performed), placental invasion of the uterine scar, stillbirth, and maternal death, for women who cannot or do not access facilities with comprehensive essential obstetric care in subsequent pregnancies. For these reasons, in some low-resource settings, the complications associated with Caesarean section may pose greater risks to both mothers' and babies' lives than vaginal delivery for breech presentation. Vaginal birth for breech presentations is advised in these circumstances, as 97% of breech-position singleton infants will be born without complications [97]. Additionally, adoption of a policy of breech delivery by Caesarean section will ultimately lead to the disappearance of the specialised clinical skills required to perform vaginal breech delivery, meaning that women with breech fetuses who do deliver vaginally may face greater risk.

### Magnesium sulphate supplementation for pre-eclampsia/eclampsia and pre-term labour

#### Background

Magnesium sulphate has both anti-convulsant and tocolytic applications. Anti-convulsants, including magnesium sulphate, diazepam, and phenytoin, are a key strategy for preventing or stopping eclamptic seizures in pregnant women with pre-eclampsia and eclampsia, respectively, which account for 50,000 maternal deaths per year worldwide (10% of all direct maternal deaths) [101]. Magnesium sulphate is generally regarded as the first choice drug, and superior to diazepam or phenytoin, for controlling eclamptic fits and preventing associated maternal and fetal deaths [102-104]. However, the use of magnesium sulphate presents potential hazards including severe maternal adverse effects such as respiratory and cardiac arrest, and risk of fetal neurological depression (e.g., decreased respiratory effort), suggesting that while magnesium sulphate may prevent some fetal and neonatal deaths due to eclampsia, it may present an increased risk of harm in some pregnancies.

Magnesium sulphate also has tocolytic properties: it relaxes smooth muscle and inhibits uterine contractile activity. Tocolytic drugs can theoretically prevent or delay labour and pre-term birth for women at high risk of pre-term delivery [105]. Ideally, a tocolytic agent would delay labour long enough to administer corticosteroids to hasten fetal lung maturation prior to delivery. Magnesium sulphate is widely used as a tocolytic agent for preventing pre-term birth in the United States [106]; below, we explore the evidence for the impact of its use on stillbirth.

#### Literature-based evidence

Our literature search identified 4 Cochrane reviews on the role of magnesium sulphate for management of pre-eclampsia and eclampsia and 3 Cochrane reviews and 1 other study on its role in threatened preterm labour (Table 7). The role of antenatal magnesium supplementation in prevention of pre-eclampsia in magnesium-deficient populations has been discussed in a previous paper in this series [107].

**Table 7:** Impact of magnesium sulphate in treatment of pre-eclampsia/eclampsia and threatened pre-term labour on stillbirth and perinatal mortality

**Source**	**Location and Type of Study**	**Intervention**	**Stillbirths/Perinatal Outcomes**
**Magnesium sulphate for treatment of pre-eclampsia and eclampsia**			

***Reviews and meta-analyses***			

Duley 2003 [108]	Bangladesh, South Africa, USA, Malaysia. Meta-analysis (Cochrane). 5 RCTs included (N = 9,961 women).	To assess the effects of magnesium sulphate for pre-eclampsia (intervention) vs. placebo or no anti-convulsant (controls) on the women and their children.	SBR: RR = 0.99 (95% CI: 0.87 – 1.12) **[NS]**. [424/5003 vs. 426/4958 in intervention and control groups, respectively]. PMR: RR = 0.98 (95% CI: 0.88 – 1.10) **[NS]**. [538/4655 vs. 541/4604 in intervention and control groups, respectively].

Duley et al. 2000 [109]	India. Meta-analysis (Cochrane). 2 RCTs included (N = 177 women).	To compare the effects of magnesium sulphate (intervention) vs. those of lytic cocktail (controls) when used for the care of women with eclampsia.	SBR: RR = 0.55 (95% CI: 0.26 – 1.16) **[NS]**. [9/89 vs. 16/88 in intervention and control groups, respectively]. NMR: RR = 0.39 (95% CI: 0.14 – 1.06) **[NS]**. [5/90 vs. 13/93 in intervention and control groups, respectively].

Duley et al. 2003 [104]	South Africa, India. Meta-analysis (Cochrane). 2 RCTs included (N = 665 women).	To assess the effects of magnesium sulphate (intervention) vs. phenytoin (controls) when used for the care of women with eclampsia.	SBR: RR = 0.83 (95% CI: 0.61 – 1.13) **[NS]**. [57/325 vs. 72/340 in intervention and control groups, respectively]. PMR: RR = 0.85 (95% CI: 0.67 – 1.09) **[NS]**. [84/325 vs. 103/340 in intervention and control groups, respectively]. NMR: RR = 0.95 (95% CI: 0.59 – 1.53) **[NS]**. [29/325 vs. 32/340 in intervention and control groups, respectively].

Duley et al. 2003 [103]	Malaysia, Zimbabwe, Africa, Asia and South America. Meta-analysis (Cochrane). 4 RCTs included (N = 756 women).	To assess the effects of magnesium sulphate (intervention) vs. diazepam (controls) when used for the care of women with eclampsia.	SBR: RR = 0.89 (95% CI: 0.63 – 1.26) **[NS]**. [51/385 vs. 55/371 in intervention and control groups, respectively]. PMR: RR = 1.04 (95% CI: 0.80 – 1.36) **[NS]**. [87/379 vs. 80/366 in intervention and control groups, respectively]. NMR: RR = 1.34 (95% CI: 0.84 – 2.14) **[NS]**. [38/364 vs. 27/352 in intervention and control groups, respectively].

**Magnesium sulphate for threatened pre-term labour.**			

***Reviews and meta-analyses***			

Crowther et al. 2002 [105]	USA. Meta-analyses (Cochrane). 7 RCTs included (N = 635 women).	To assess the effectiveness and safety of magnesium sulphate therapy (intervention) vs. placebo, no placebo or alternative tocolytic therapy (controls) given to women in threatened pre-term labour with the aim of preventing pre-term birth and its sequelae.	Fetal deaths (miscarriage+SB): RR = 5.70 (95% CI: 0.28 – 116.87) **[NS]**. [2/293 vs. 0/342 in intervention and control groups, respectively].

Crowther and Moore 1998 [111]	USA. Cochrane review. 1 RCT included (N = 50 women).	To assess the effects of magnesium maintenance therapy (intervention) vs. placebo/no treatment (controls) on preventing pre-term birth after threatened pre-term labour.	Death before hosp discharge: RR = 5.00 (95% CI: 0.25 – 99.16) **[NS]**. [2/25 vs. 0/25 in intervention and control groups, respectively].

Doyle et al. 2007 [110]	Australia, New Zealand, France, USA. Meta-analysis (Cochrane). 4 RCTs included (N = 3,701 women).	To assess the effectiveness and safety of magnesium sulphate as a neuroprotective agent (intervention) vs. placebo or no placebo (controls) when given to women considered at risk of pre-term birth.	Fetal death (miscarriage + SB): RR = 0.98 (95% CI: 0.78 – 1.24) **[NS]**. [123/1864 vs. 125/1837 in intervention and control groups, respectively].

***Intervention studies***			

Rouse et al. 2008 [112]	USA. Multicentre. RCT. N = 2241 women 24–31 weeks of gestation deemed at high risk of pre-term labour.	Compared the impact of IV magnesium sulphate (a loading dose of 6 g infused for 20 to 30 minutes, followed by a maintenance infusion of 2 g per hour) (intervention) with identical-appearing placebo (controls).	SBR+IMR): RR = 1.12 (95% CI: 0.85 – 1.47); P = 0.41 **[NS]**. [99/1041 (9.5%) vs. 93/1095 (8.5%) in intervention and control groups, respectively]. Moderate or severe cerebral palsy: RR = 0.55 (95% CI: 0.32–0.95); P = 0.03. [20/1041 (1.9%) vs. 38/1095 (3.5%) in intervention and control groups, respectively].

##### Magnesium sulphate for treatment of pre-eclampsia and eclampsia

A Cochrane review by Duley et al. [108] (Additional file 17) compared the impact of magnesium sulphate versus placebo or no treatment in women with pre-eclampsia, and reported no difference in the risk of stillbirth and/or neonatal death (3 trials; RR = 1.04, 95% CI: 0.93–1.15) ***[LOE: 1++]***. A second Cochrane review by Duley et al. [103] (Additional file 18) comparing magnesium sulphate with diazepam in eclampsia cases showed no difference in stillbirth rates between the two treatments (RR = 0.89, 95% CI: 0.63–1.26); a separate Cochrane review [104] comparing magnesium sulphate to phenytoin found a similar non-significant impact on stillbirth rates (2 trials, 665 babies, RR = 0.83, 95% CI: 0.61–1.13). However, magnesium sulphate was associated with fewer admissions of babies to a neonatal special care unit (1 trial, N = 518 babies, RR = 0.73, 95% CI: 0.58–0.91), and fewer neonatal deaths or special care unit admissions exceeding 7 days (1 trial, N = 665 babies, RR = 0.77, 95% CI: 0.63–0.95) ***[LOE: 1+] ***(Additional file 19).

Duley et al. [109] (Additional file 20) evaluated RCTs comparing any use of magnesium sulphate with any use of lytic cocktail (a combination of drugs, usually chlorpromazine, promethazine and pethidine) in women with eclampsia (2 trials, N = 199 women). Magnesium sulphate proved superior to lytic cocktail in preventing further seizures (RR = 0.09, 95% CI: 0.03–0.24), but the comparative impact of magnesium sulphate on stillbirths was not statistically significant (RR = 0.55, 95% CI-0.26–1.16) ***[LOE: 1+]***.

##### Magnesium sulphate for prevention of preterm birth

Doyle et al. [110] reviewed RCTs of studies evaluating the impact of antepartum magnesium sulphate therapy in women at high risk of, or with threatened, pre-term labour (4 trials, N = 3701 babies) (Additional file 21). There was no difference in risk of fetal death between women administered magnesium sulphate versus no magnesium sulphate (RR = 0.98, 95% CI: 0.78–1.24, 4 trials). Additionally, there was no impact of antepartum magnesium sulphate therapy on paediatric mortality (RR = 0.97, 95% CI: 0.74–1.28, 4 trials, 3701 infants) or neurological impairments or disabilities in early childhood ***[LOE: 1++]. ***A second, large Cochrane review of the use of magnesium sulphate to prevent preterm birth by Crowther et al. [105] reported an elevated risk of baby death (fetal plus infant death) among infants exposed to magnesium sulphate (RR = 2.82, 95% CI: 1.20–6.62, 7 trials, 727 infants) (Additional file 22). Considering fetal deaths separately, there was no significant impact on risk of fetal death (1 trial, RR = 5.70, 95% CI: 0.28–116.87 [NS]); both fetal deaths occurred in just 1 of the 7 studies reporting fetal or child death. Magnesium sulphate made no difference in the risk of delivery within 48 hours of treatment (RR = 0.85, 95% CI: 0.58–1.25, 11 trials, 881 women) compared to controls not given magnesium sulphate. Similarly, no benefit was seen for magnesium sulphate on the risk of giving birth pre-term (<37 weeks) or very pre-term (<34 weeks). The third Cochrane review [111] on magnesium maintenance therapy after administration of an initial tocolytic for preventing preterm births, had 3 trials, including 303 women. The magnesium group had a five-fold higher risk of perinatal mortality than placebo, no or alternative treatment (Additional file 23).

A multicentre RCT of magnesium sulphate use to reduce cerebral palsy and death by Rouse et al. [112] in women at risk of preterm birth between 24 and 31 weeks of gestation showed that the women in the intervention group had a statistically non-significant slightly elevated risk of the composite outcome of stillbirth or infant death (RR = 1.12; 95% CI: 0.85–1.47). Risk of moderate or severe cerebral palsy was significantly lower in children of women given magnesium sulphate compared to placebo (RR = 0.55; 95% CI: 0.32–0.95) ***[LOE 1+]***.

#### Conclusion

Intravenous or intramuscular magnesium sulphate significantly decreases the risk of eclampsia. IV or intramuscular magnesium sulphate appears to be substantially more effective than diazepam and phenytoin for treatment of eclampsia, and is therefore the treatment of choice. No trials reported any statistically significant impact of magnesium sulphate on stillbirth incidence or perinatal mortality.

Among women deemed to be at risk of preterm birth, magnesium sulphate is ineffective at delaying or preventing labour, and while its use may be associated with side effects in the infant such as neurological depression, there is evidence that magnesium sulphate may also prevent moderate or severe cerebral palsy in live-born infants [112]. There is not enough evidence to show any difference between magnesium maintenance therapy and either placebo or no treatment, or alternative therapies (other tocolytics, for example) in preventing preterm birth after an episode of threatened preterm labour. Thus, despite good quality evidence and impact on maternal pre-eclampsia (Grade A), evidence for impact of magnesium therapy on stillbirth prevention is insufficient.

### Maternal hyperoxygenation for suspected impaired fetal growth

#### Background

Long-term fetal hypoxia, which results from diminished oxygen and/or nutrient flow to the fetus from the mother, is often implicated in cases of impaired fetal growth. Impaired fetal growth may result from a number of factors, including fetal characteristics (e.g., congenital abnormalities), placental factors (e.g., small placenta, poor placentation), and maternal conditions (e.g., drug use, malnutrition, renal or vascular problems) [113]. Severe deprivation of oxygen and/or nutrients can result in hypoxia or nutrient deprivation so severe as to cause stillbirth. Several observational uncontrolled trials have evaluated the physiological basis for maternal hyperoxygenation (40–55% humidified oxygen by face mask at 8 litres per minute, 24 hours per day) as a way to improve oxygen flow to the fetus and thereby alleviate hypoxia and stimulate nutrient transfer [114-118].

#### Literature-based evidence

Our literature search identified 1 Cochrane review and 1 other intervention study (Table 8) that evaluated the impact of maternal hyperoxygenation on perinatal mortality. Say et al. [119] reviewed RCTs (3 RCTs, N = 94 women) comparing maternal oxygen therapy with no oxygen therapy in suspected impaired fetal growth, and found statistically significantly lower PMR in the oxygenation group compared to the non-oxygen group in all three included trials (RR = 0.50, 95% CI: 0.32–0.81). However, the reviewers cautioned that higher gestational age in the oxygenation groups might have accounted for the difference in mortality rates ***[LOE: 1++] ***(Additional file 24).

**Table 8:** Impact of maternal hyperoxygenation for impaired fetal growth on stillbirth and perinatal mortality

**Source**	**Location and Type of Study**	**Intervention**	**Stillbirths/Perinatal Outcomes**
***Reviews and meta-analyses***			

Say et al. 2003 [119]	Italy, UK, South Africa. Meta-analysis (Cochrane). 3 RCTs included (N = 94 women).	To assess the effects of maternal oxygen therapy (intervention) vs. management without additional oxygen (controls) in suspected impaired fetal growth on fetal growth and perinatal outcome.	PMR: RR = 0.50 (95% CI: 0.32 – 0.81). [15/46 vs. 31/48 in intervention and control groups, respectively].

***Intervention studies***			

Battaglia et al. 1994 [120]	Italy (Modena). Tertiary referral hospital (University of Modena). Quasi-RCT. N = 38 patients with intrauterine growth retardation (N = 18 intervention group, N = 20 controls).	Compared the impact on fetal survival of bed rest plus humidified 55% oxygen at a rate of 8 l/min continuously (intervention) vs. bed rest (plus anti-hypertensive treatment, when necessary). Ultrasound assessment of amniotic fluid volume was performed on alternate days, and the fetal abdominal circumference was evaluated weekly. Doppler analysis of fetal/maternal circulation was performed upon the patient's arrival at hospital, after 12 h, and thereafter on alternate days until parturition.	PMR: No deaths.

A quasi-RCT by Battaglia et al. [120] attempted to compare the impact of maternal oxygenation versus no oxygen in a group of pregnant women (N = 38) prescribed bed rest, but no perinatal deaths occurred in either group ***[LOE: 2-]***.

#### Conclusion

The evidence for maternal hyperoxygenation in the Cochrane suggests that in pregnant women treated with long-term oxygen therapy, perinatal mortality is significantly reduced [119]. Methodological deficiencies in the trials included in the Cochrane review, including the greater average gestational age of fetuses in the oxygen intervention groups and the small size of the included trials, suggest that this promising finding must be confirmed with rigorous, large multicentre RCTs reporting stillbirths and early neonatal mortality separately. Interestingly, while there appeared to be an impact on perinatal mortality, maternal hyperoxygenation did not appear to improve fetal growth. We classify maternal hyperoxygenation as having some evidence of benefit in reducing perinatal mortality given the promising findings of the Cochrane review on this subject, but suggest that this intervention not be included in programs until confirmatory results become available, as some prior studies have suggested that maternal hyperoxygenation may inadvertently reduce uterine blood flow, and that long-term oxygen therapy may be associated with maternal pulmonary dysfunction [121-123].

### Amnioinfusion

#### Background

Amnioinfusion is a procedure whereby fluid (either saline or Ringer's lactate) is added to the uterine cavity transcervically via catheter, when the membranes have ruptured, or transabdominally using a needle if amniotic membranes are still intact. The procedure is often performed in an effort to prevent or relieve umbilical cord compression during labour associated with low amniotic fluid volume (oligohydramnios); the infusion of fluid also dilutes meconium detected in the amniotic fluid. Dilution of meconium is thought to minimize the risk of meconium aspiration, as thick meconium in cases of oligohydramnios is associated with significant perinatal mortality and morbidity [124]. Amnioinfusion may impact fetal survival via one or both of these mechanisms and the two can be difficult to differentiate. Amnioinfusion has also been used in an effort to prevent ascending infection in cases of premature rupture of membranes (PROM)[125], or to facilitate external cephalic version at term, though the impact of amnioinfusion for external cephalic version on perinatal outcomes has not been examined [126]. The evidence for whether amnioinfusion for this variety of indications has any impact on perinatal mortality outcomes has not been systematically compiled.

#### Literature-based evidence

The literature search identified 2 Cochrane reviews and 11 other interventional/observational studies, presented below by indication (Table 9).

**Table 9:** Impact of amnioinfusion on stillbirth and perinatal outcomes

**Source**	**Location and Type of Study**	**Intervention**	**Stillbirths/Perinatal Outcomes**
***Reviews and meta-analyses***			

Hofmeyr 2002 [124]	South Africa, Zimbabwe, USA. Meta-analysis (Cochrane). 8 RCTs included (N = 1,481 women).	To assess the effects of amnioinfusion for meconium-stained liquor (intervention) vs. no amnioinfusion (controls) on perinatal outcome.	PMR: RR = 0.34 (95% CI: 0.11 – 1.06) **[NS]**. [4/727 vs. 12/754 in intervention and control groups, respectively].

Hofmeyr 1998 [125]	USA. Meta-analysis (Cochrane). 8 RCTs included (N = 584 women).	To assess the effects of amnioinfusion (intervention) vs. no amnioinfusion (controls) on maternal and perinatal outcome for potential or suspected umbilical cord compression or potential amnionitis.	PMR: RR = 0.51 (95% CI: 0.11 – 2.24) **[NS]**. [2/301 vs. 4/283 in intervention and control groups, respectively].

***Intervention studies***			

Ashfaq 2004 [130]	Pakistan (Karachi). Jinnah Postgraduate Medical Centre. Matched case control study. N = 400 patients between 1^st ^January 1998 to 31^st ^December 2000 (N = 200 intervention group, N = 200 controls) with meconium staining of liquor.	Compared the impact on fetal outcome of amnioinfusion (intervention) vs. no amnioinfusion (controls) in cases of meconium staining.	SBR: 0/200 vs. 8/200 (4%) in intervention and control groups, respectively. PMR/perinatal morbidity: 6% vs. 14% in intervention and control groups, respectively (statistically significant).

Das et al. 2007 [133]	India (West Bengal). Prospective comparative study. Women (N = 150) who were in labour and had meconium-stained amniotic fluid (N = 50 intervention group, N = 100 controls).	Compared the impact of transcervical amnioinfusion (intervention) vs. standard care (controls).	PMR: RR = 0.31 (95% CI: 0.07 – 1.31) **[NS]**. [2/50 (4%) vs. 13/100 (13%) in intervention and control groups, respectively].

Fraser et al. 2005 [129]	13 countries. Multicentered (56 centers). RCT. Pregnant women (N = 1998) in labour at 36 or more weeks of gestation who had thick meconium staining of the amniotic fluid. (81.3% of these women did not have recurrent variable decelerations in fetal heart rate on monitoring). N = 995 intervention group, N = 1003 controls).	Compared the impact of transcervical amnioinfusion (800 ml saline over 40 min, followed by 2 ml/min to 1500 ml max; intervention) vs. standard care (no amnioinfusion) (controls). Women were assessed by continuous monitoring of intrauterine pressure or by uterine palpation at 15-minute intervals for signs of uterine overdistention or hypertonic contractions. Continuous electronic fetal heart-rate monitoring was performed in both groups.	PMR: RR = 1.00 (95% CI: 0.29 – 3.45) **[NS]**. [N = 5 (0.5%) vs. N = 5 (0.5%) in intervention and control groups, respectively]. PMR, moderate or severe meconium aspiration syndrome, or both: RR = 1.26 (95% CI: 0.82 – 1.95) **[NS]**. [44/986 (4.5%) vs. 35/989 (3.5%) in intervention and control groups, respectively].

Kirubamani 2000. [132]	India. RCT. N = 50 labouring women with clinically analysed meconium (light, moderate, thick) (N = 30 intervention group, N = 20 controls).	Compared the impact on perinatal mortality of amnioinfusion with warm saline at room temperature, along with standard obstetric care (intervention) vs. standard care only without amnioinfusion (controls).	PMR: 0/30 vs. 1/20 in intervention and control groups, respectively.

Mukhopadhyay et al. 2006 [153]	India. Quasi-RCT. N = 200 women (N = 100 in each group).	Compared the impact on perinatal mortality of intraamniotic infusion of normal saline (intervention) vs. no amnioinfusion (controls).	PMR: 2/93 (2.1%) vs. 3/93 (3.2%) in intervention and control groups, respectively; P = 0.9748.

Rathore et al. 2002 [134]	India. RCT. Women (N = 200) during labour with meconium stained amniotic fluid (N = 100 in each group).	Assessed the effect on perinatal deaths of amnioinfusion (intervention) vs. no amnioinfusion (controls).	PMR: 2 vs. 5 deaths in intervention and control groups, respectively. SBR: 1 death in each group. Early NMR (excluding malformations): 0 vs. 1 death in intervention and control groups, respectively.

***Observational studies***			

Chhabra et al. 2007 [128]	India. Case-control study. Pregnant women (N = 100) with oligohydramnios (N = 50 study group, N = 50 controls).	Compared the impact of antepartum transabdominal amnioinfusion (cases) vs. conservative treatment without amnioinfusion (controls).	PMR: 4% vs. 18% in cases and controls, respectively (statistically significant).

Das 2001 [154]	India. Prospective case control study. Women (N = 290); (N = 100 amnioinfusion group, N = 190 controls).	Compared the impact on perinatal mortality of amnioinfusion (intervention) vs. no amnioinfusion (controls).	PMR: 1/100 vs. 16/190 in amnioinfusion and control groups, respectively; P = 0.01.

De Santis et al. 2003 [127]	Italy. Tertiary care center. Quasi-RCT. Women (N = 71) with pre-term premature rupture of membranes (pPROM) at <26 weeks of gestational age (N = 37 amnioinfusion group, N = 34 controls).	Compared the impact on fetal survival of serial transabdominal amnioinfusion with saline every 7 days in case of persistent oligohydramnios (intervention) vs. expectant management (controls).	Intrauterine fetal survival: 24/37 (64.8%) vs. 11/34 (32.3%) in intervention and control groups, respectively, p < 0.01.

Halvax 2002 [155]	Hungary. Tertiary referral hospital (University of Pecs). Retrospective analysis. N = 228 women (N = 118 amnioinfusion group, N = 110 controls).	Compared the impact of simultaneous use of fetal pulse oximetry and amnioinfusion in meconium stained amniotic fluid (intervention) vs. no amnioinfusion (controls). All monitored with cardiotocography.	Meconium below the vocal cords: 0% vs. 10.1% in intervention and control groups, respectively; P < 0.01. Operative delivery rate: 22.0% vs. 30.9% in intervention and control groups, respectively; P < 0.05.

Sahu 2003 [131]	India. Prospective case-control study. Women (N = 250) having meconium stained amniotic fluid during labour (N = 100 amnioinfusion group, N = 150 controls.	Compared the impact on perinatal mortality of amnioinfusion (study group) vs. no amnioinfusion (controls).	PMR: 1/100 (1%) vs. 12/150 (8%) in the study and control groups, respectively; P = 0.01.

##### Amnioinfusion for PROM

The original Cochrane review by Hofmeyr et al. on amnioinfusion [125] evaluated 14 relatively small (<200 participants) RCTs of amnioinfusion compared with no amnioinfusion in pregnancies with PROM as a treatment to alleviate umbilical cord compression and prevent intrauterine infection. There was no significant impact of amnioinfusion compared with controls on perinatal mortality (8 trials, RR = 0.51, 95% CI: 0.11–2.24) (Additional file 25). Transcervical amnioinfusion for potential or suspected umbilical cord compression reduced the risk of fetal heart rate decelerations (4 trials, N = 227 women: RR = 0.54; 95% CI: 0.43–0.68); Caesarean section (9 trials, N = 953 women, RR = 0.52, 95% CI: 0.40–0.69), Apgar score < 7 at 5 minutes (7 trials, N = 828 women, RR = 0.54, 95% CI: 0.30–0.97), and low cord arterial pH (6 trials, N = 660 women, RR = 0.45, 95% CI 0.31–0.64). Transabdominal amnioinfusion showed similar trends, though numbers studied were too small to reach statistical significance. Transcervical amnioinfusion to prevent infection in women with membranes ruptured for more than six hours (one trial of 66 women) was associated with a reduction in puerperal infection (1 trial, N = 68 women, RR = 0.50, CI 0.26–0.97) ***[LOE: 1-].***

In a tertiary care facility in Italy, a quasi-RCT by De Santis et al. 2003 [127] assessed the impact on fetal survival of weekly transabdominal amnioinfusion in a group of women with pre-term PROM at less than 26 weeks gestation (N = 71), reporting twice the survival rate among fetuses born to women in the amnioinfusion group versus the expectantly managed group (64.8% vs 32.3% of fetuses survived, respectively, P < 0.01).

##### Amnioinfusion for oligohydramnios with intact membranes

A case-control study of women with oligohydramnios in pregnancy (N = 100) by Chhabra et al. [128] administered antepartum transabdominal amnioinfusion to 50 women, treating the remainder conservatively. The perinatal mortality rate was significantly lower among the amnioinfusion group compared to controls given conservative management (4% vs. 18%, respectively) ***[LOE: 2++]***.

##### Prevention of meconium aspiration

A second Cochrane review by Hofmeyr [124] evaluated RCTs (12 small trials) comparing amnioinfusion with no amnioinfusion for women in labour under either normal or limited perinatal surveillance with moderate or thick meconium staining of the amniotic fluid (Additional file 26). Amnioinfusion was associated with significant reductions in heavy meconium staining of the amniotic fluid (RR = 0.03, 95% CI: 0.01–0.15) and variable fetal heart rate deceleration (RR = 0.65, 95% CI: 0.49–0.88). No perinatal deaths were reported. Under limited perinatal surveillance, amnioinfusion was associated with a trend towards reduced perinatal mortality (RR = 0.34, 95% CI: 0.11–1.06 **[NS]**), as well as significantly decreased risk of meconium aspiration syndrome (RR = 0.24, 95% CI: 0.12–0.48), neonatal hypoxic ischaemic encephalopathy (RR = 0.07, 95% CI: 0.01–0.56) and neonatal ventilation or intensive care unit admission (RR = 0.56, 95% CI: 0.39–0.79); ***[LOE: 1+].***

A large, multicentre RCT (56 centres, 13 countries, N = 1998 women) by Fraser et al. [129] of transcervical amnioinfusion versus standard care in women with thick meconium staining reported 5 perinatal deaths in each group, suggesting no beneficial impact of amnioinfusion over standard care. The study also suggested the possibility that the risk of meconium aspiration syndrome could be elevated rather than reduced after amnioinfusion relative to controls (RR = 1.39, 0.88–2.19 [NS]).

A matched case-control study by Ashfaq and Shah [130] in Pakistan tested the impact of amnioinfusion in cases of meconium stained liquor (N = 400 women). Both perinatal morbidity and mortality were reduced in the amnioinfusion group relative to controls (6% vs. 14%, respectively), and the stillbirth rate was 0/200 (0%) in the amnioinfusion group versus 8/200 (4%) in the control group, though no statistical significance data was given. The prevalence of meconium aspiration syndrome was lower in the amnioinfusion group than among controls (22% vs. 56%, respectively).

Several relatively small studies on amnioinfusion for meconium staining in India reported lower rates of perinatal mortality in the amnioinfusion groups. A case-control study of amnioinfusion in women with meconium staining (N = 250) reported substantially fewer deaths in the amnioinfusion group than among controls (1/100 [1%] vs. 12/150 [8%], respectively, P = 0.01) [131]. Kirubamani [132] tested the impact of intrapartum amnioinfusion with saline versus standard care in women with light, moderate, or thick meconium (N = 50), and documented no deaths in the intervention group versus one among controls (0/30 vs. 1/20, respectively). In West Bengal, a study by Das et al. [133] of women in labour with meconium-stained amniotic fluid (N = 150) found that the group that received transcervical amnioinfusion (N = 50) showed a possible trend toward reduced perinatal mortality compared to a group (N = 100) receiving standard care (4% vs. 13%, respectively; RR = 0.31; 95% CI: 0.07–1.31). Rathore [134] performed a trial of amnioinfusion during labour for women with meconium staining (N = 200), and found one stillbirth in the amnioinfusion group and one stillbirth and one neonatal death in the control group ***[LOE: 1+]***.

#### New meta-analysis

**Figure 8:**
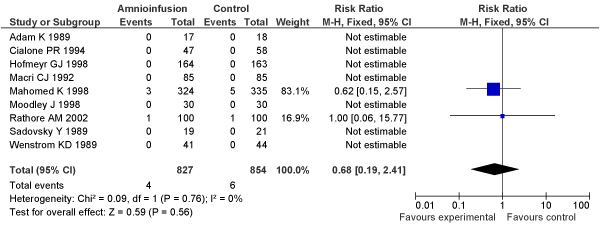
**Meta-view: Impact of amnioinfusion for meconium-stained liquor on stillbirth (Fixed model)**.

**Figure 9:**
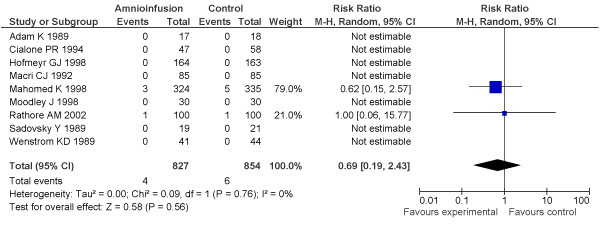
**Meta-view: Impact of amnioinfusion for meconium-stained liquor on stillbirth (Random model)**.

**Figure 10:**
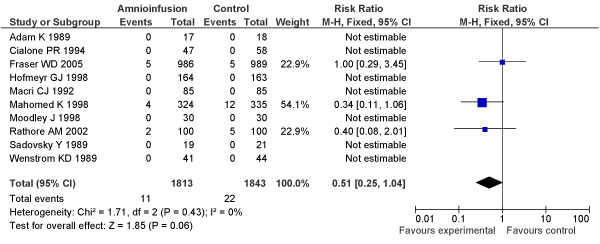
**Meta-view: Impact of amnioinfusion for meconium-stained liquor on perinatal mortality (Fixed model)**.

**Figure 11:**
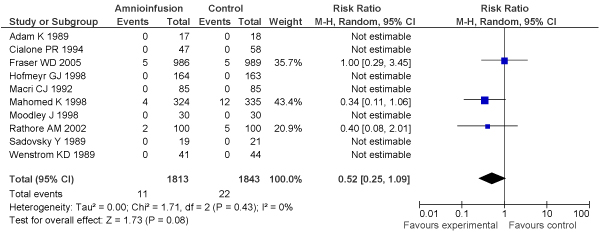
**Meta-view: Impact of amnioinfusion for meconium-stained liquor on perinatal mortality (Random model)**.

Our literature search identified ten randomised and quasi-randomised trials reporting an impact of amnioinfusion on stillbirths (9 trials, N = 1681 women) and perinatal mortality (10 trials, N = 3656 women). Pooled analysis of the impact of amnioinfusion on stillbirth incidence revealed a non-significant reduction in risk associated with amnioinfusion (RR [fixed] = 0.68, 95% CI: 0.19–2.41 [NS]; RR [random] = 0.69, 95% CI: 0.19–2.43 [NS]) (Figures 8 and 9). Comparing the impact of amnioinfusion versus controls without amnioinfusion on perinatal deaths yielded a trend toward reduced mortality (RR = 0.51, 95% CI: 0.25–1.04, RR [random] = 0.52, 95% CI: 0.25–1.09 [NS]) (Figures 10 and 11).

#### Conclusion

In low-resource settings, paediatric facilities for the management of meconium aspiration syndrome are scarce and interventions to prevent meconium aspiration are needed. Amnioinfusion, as one preventive option, is comparatively more feasible than management of meconium aspiration syndrome in settings with limited intrapartum facilities. Pooled data from the Hofmeyr meta-analysis support the use of amnioinfusion for meconium stained amniotic fluid to reduce the incidence of meconium aspiration syndrome, with a trend toward reduced perinatal mortality. However, the only large RCT on this subject by Fraser et al [129], performed after the Cochrane review, found no statistically significant impact on either meconium aspiration syndrome or perinatal mortality, suggesting the possibility of small study bias (an overrepresentation of published small trials favouring a treatment effect) in the Cochrane review [135].

For other antepartum and intrapartum indications for amnioinfusion, including PROM, oligohydramnios with intact membranes, alleviation of umbilical cord compression, and prevention of infection after membrane rupture, the available evidence is too limited to formulate recommendations on the use of amnioinfusion. Some small studies show promising differentials in mortality and other outcomes between intervention and control groups, but larger, more rigorous studies are needed to determine the impact of amnioinfusion on these outcomes.

Further studies of amnioinfusion are needed to confirm the obstetric indications and administration techniques by which amnioinfusion might reduce perinatal mortality. When performing amnioinfusion in low-resource settings, providers should remain vigilant about the risk of infection if aseptic conditions are not maintained.

## Summary

While few studies reported consistent and statistically significant evidence of impact on perinatal mortality associated with the intrapartum interventions we reviewed, several interventions show promising indications of benefit for specific indications or in certain settings. Induction of labour rather than expectant management in post-term pregnancies showed strong evidence of impact, though the choice of drug(s) for induction of labour remains unclear. Planned Caesarean section for term breech presentation has been shown in a large RCT to reduce stillbirths three-fold compared to vaginal breech delivery, but questions of feasibility, potential enrolment bias, and consequences of implementing this intervention routinely in low-/middle-income countries prevent a universal recommendation for its practice. Magnesium sulphate for pre-eclampsia and eclampsia is effective in preventing eclamptic seizures, but studies, many underpowered, did not demonstrate an impact on perinatal mortality. Transcervical amnioinfusion for meconium staining appears promising for improving perinatal outcomes in low/middle income-country applications according to the findings of many small studies, but a large randomised trial of the intervention had no significant impact on perinatal mortality Other novel interventions like maternal hyperoxygenation had statistically significant evidence of impact on stillbirth, but the limited evidence base requires confirmation by other studies.

A distillation of the weight of the evidence for each of the 8 intrapartum interventions we reviewed is presented in Table 10.

**Table 10:** Summary of evidence grading for all interventions during the intrapartum period to prevent stillbirth and perinatal mortality reviewed in this paper

	**Evidence of no or negative impact** (leave out of programmes)	**Uncertain evidence** (need for additional research before including in programmes)	**Some evidence** (may include in programmes, but further evaluation is warranted)	**Clear evidence** (merits inclusion in programmes)
Instrumental delivery (vacuum vs. forceps)			X (neither method superior, but either or both should be in programs)	

Comprehensive emergency obstetric care packages, including Caesarean section				X

Induction of labour (vs. expectant management)				X (for post-term pregnancy only)

Drugs for cervical ripening and induction of labour		X		

Planned Caesarean for breech presentation			X (attendant risks in low-resource settings with poor EOC access)	

Maternal hyperoxygenation for impaired fetal growth		X (some evidence but biodynamics poorly understood)		

Amnioinfusion		X		

Magnesium sulphate for pre-eclampsia/eclampsia and pre-term labour		X		

### Implications for programmes

While the implementation of any of the interventions examined in this paper could potentially prevent a stillbirth, multiple variables inherent to intrapartum care influence the outcome and complicate comparison between studies and assessment of the evidence of impact. Drug dosages, route of administration, and regimens often vary. The risk of a poor outcome of a forceps or vacuum extraction, or vaginal breech birth, is likely to be lower in the hands of an experienced versus an inexperienced practitioner. In the absence of adequate antisepsis or availability of clean water supplies and antibiotics, interventions such as instrumental delivery, Caesarean section and amnioinfusion may increase the risk of harm to the fetus or mother from infection, despite having been developed to save these lives. Monitoring quality and consistency during labour, prompt recognition of complications, and rapid performance of appropriate interventions while avoiding inappropriate interventions that put mother and fetus at unnecessarily increased risk are hallmarks of obstetric care quality that are difficult to assess, and likely varied, in the studies we reviewed.

The available evidence indicates broadly that where women receive high-quality intrapartum care, including monitoring of labour with access to operative delivery (instrumental delivery, whether forceps or vacuum, or Caesarean section), rates of perinatal deaths decrease. Particularly in low-resource settings, avoiding liberal use of Caesarean section, even for breech presentation at term, is advised.

Several interventions appear relatively well supported by the evidence. Timely delivery in the presence of intrapartum complications or maternal risk factors, often by Caesarean section or instrumental delivery, can reduce associated intrapartum stillbirth. This intervention is largely credited for the relatively low rates of intrapartum stillbirth in high-income countries [3]. Induction of labour is beneficial at or after 41 or 42 weeks for post-term pregnancy, especially if early ultrasound dating was performed to confirm gestational age. Vacuum extraction and forceps have different risks and benefits, but use of either method is justified; more essential than the instrument chosen is the need for facilities to be equipped to provide safe instrumental delivery and Caesarean section.

Planned Caesarean section for breech delivery at term reduces perinatal mortality three-fold compared to vaginal breech delivery, and where most births are in facilities that can provide safe Caesarean section, the available evidence supports providers and their clients planning a Caesarean section for breech presentation with informed consent. In low-resource settings with poor access to EmOC, however, having a Caesarean scar introduces risk of subsequent poor pregnancy outcome and maternal death, so vaginal breech delivery should be encouraged, commensurate with skills of the birth attendants. Moreover, Goldenberg et al. [3] and McClure EM et al [21] showed that population-based rates of Caesarean section exceeding 10% had no further impact on stillbirth incidence.

### Research gaps

As research of intrapartum interventions reporting stillbirth as a primary or secondary outcome is rarely conducted, many research gaps exist, making this an important area for future research (Table 11). None of the studies included in this review reported a consistent, statistically significant Grade A evidence of impact on stillbirth incidence. It follows that large RCTs (wherever ethically and logistically possible) powered to detect changes in stillbirth incidence are still needed for virtually all the interventions we reviewed.

**Table 11:** Research gaps investigating interventions to prevent intrapartum stillbirths

***Biodynamics and descriptive studies***			
• Frequency of uterine rupture in pregnancies subsequent to Caesarean section in rural settings
• Dynamics of maternal hyperoxygenation in placental perfusion and feto-placental circulation (risk or benefit to fetus?)
• Drug safety studies (fetal/neonatal outcomes): magnesium sulphate
• Safety of misoprostol for induction of labour
◦ Vaginal misoprostol optimal dosing and dose-range studies

***Pilot/clinical/cohort studies of interventions***			

• Trials/comparisons of lesser-studied induction methods
◦ Extra-amniotic prostaglandins
◦ IV prostaglandins
• Feasibility and effectiveness of oral misoprostol administration in low-resource settings
• Foley catheter insertion for pre-induction cervical ripening
• Transabdominal amnioinfusion, especially in cases of intact membranes
• Effective interventions for pre-term labour
• Acceptability and utility of inexpensive manual vacuum extractors compared to forceps for assisted vaginal delivery

***Rigorously designed large RCTs powered to detect impact on stillbirth***			

• Induction vs. expectant management for macrosomia and mild pre-eclampsia
• Comparison of first attempting assisted vaginal delivery in operating theatre vs. immediate Caesarean for obstructed labour in low-/middle-income countries
• Distress-to-decision-to-incision studies for Caesarean in low-/middle-income country settings
• Planned Caesarean vs. vaginal breech trials to confirm or refute recommendation of Term Breech Trial for routine policy of planned Caesarean for breech
• Impact of hyperoxygenation on stillbirth rate
• Impact of amnioinfusion on stillbirth rate

***Large effectiveness trials at scale or population level***			

• Unmet obstetric need studies reporting stillbirth outcomes (in addition to maternal impact)
• Association of facility quality improvement in comprehensive EOC/EmOC services with perinatal outcomes

### Conclusion

In settings where safe, comprehensive EOC is already available, and diagnostic and monitoring capacity allow, advanced interventions to manage pre-eclampsia, PPROM, and oligohydramnios are needed. A few interventions examined in this review show strong evidence of impact for certain indications, including Caesarean for breech birth at term and induction of labour for post-term pregnancy. There is some evidence that other interventions such as amnioinfusion and maternal hyperoxygenation may reduce perinatal mortality, but further research on their safety and effectiveness in a range of settings is required before they can be routinely included in programs. In areas without comprehensive essential obstetric care capacity, it is key to prioritise improved access to EmOC, especially vacuum extraction and Caesarean section. EmOC is a package of clearly life-saving interventions, and there is an association between countries with high unmet obstetric need and intrapartum stillbirth rates [3]. Safe EmOC, ideally as part of a package of comprehensive essential obstetric care services to address obstetric problems before they become emergencies, will have the greatest impact on intrapartum stillbirth rates in low-resource settings, though expanding provision of EmOC requires developing solutions for numerous logistical and infrastructural challenges. These interventions would need complementary measures to ensure staff training and optimise delivery strategies in health systems.

## List of abbreviations used

ANC: antenatal care; CI: confidence interval; CP: cerebral palsy; EmOC: emergency obstetric care; EOC: essential obstetric care; LBW: low birth weight; NMR: neonatal mortality rate; OR: odds ratio; PMR: perinatal mortality rate; RCT: randomised controlled trial; RR: relative risk; SB: stillbirth; SBR: stillbirth rate; IV: intravenous; VBAC: vaginal birth after Caesarean; WHO: World Health Organisation

## Competing interests

The authors declare that they have no competing interests.

## Authors' contributions

The paper was written and reviewed by all the authors.

## Supplementary Material

Additional file 1 **Web Table 1. Component studies in Johanson and Menon 1999 meta-analysis: Impact of vacuum vs. forceps delivery on perinatal mortality**. Component studies in Johanson and Menon 1999 meta-analysis showing impact on stillbirths/perinatal mortality. Click here for file

Additional file 2 **Web Table 2. Component studies in Johanson and Menon 2000: Impact of soft vs. rigid cup delivery on perinatal mortality**. Component studies in Johanson and Menon 2000 meta-analysis showing impact on stillbirths/perinatal mortality. Click here for file

Additional file 3 **Web Table 3. Component studies in Gulmezoglu et al. 2006 meta-analysis: Impact of induction of labour at or beyond term by cervical status on stillbirth and perinatal mortality**. Component studies in Gulmezoglu et al. 2006 meta-analysis showing impact on stillbirths/perinatal mortality. Click here for file

Additional file 4 **Web Table 4. Component studies in Irion and Boulvain 1998 meta-analysis: Impact of induction of labour for suspected fetal macrosomia on perinatal mortality**. Component studies in Irion **and Boulvain** 1998 meta-analysis showing impact on stillbirths/perinatal mortality. Click here for file

Additional file 5 **Web Table 5. Component studies in Boulvain et al. 2001: Impact of elective delivery in term diabetic pregnant women on perinatal mortality**. Component studies in Boulvain et al. 2001 meta-analysis showing impact on stillbirths/perinatal mortality. Click here for file

Additional file 6 **Web Table 6. Component studies in Dare et al. 2006 meta-analysis: Impact of planned early birth on perinatal mortality**. Component studies in Dare et al. 2006 meta-analysis showing impact on stillbirths/perinatal mortality. Click here for file

Additional file 7 **Web Table 7. Component studies in Dodd and Crowther 2003: Impact of elective delivery of women with a twin pregnancy from term on perinatal mortality**. Component studies in Dodd **and Crowther **2003 meta-analysis showing impact on stillbirths/perinatal mortality. Click here for file

Additional file 8 **Web Table 8. Component studies in Alfirevic and Weeks 2006 meta-analysis: Impact of oral misoprostol for induction of labour on perinatal mortality**. Component studies in Alfirevic and Weeks 2006 meta-analysis showing impact on stillbirths/perinatal mortality. Click here for file

Additional file 9 **Web Table 9. Component studies in Hofmeyr and Gulmezoglu. 2003 meta-analysis: Impact of vaginal misoprostol for cervical ripening and labour induction on perinatal mortality**. Component studies in Hofmeyr **and Gulmezoglu** 2003 meta-analysis showing impact on stillbirths/perinatal mortality. Click here for file

Additional file 10 **Web Table 10. Component studies in French 2001: Impact of oral prostaglandin E2 for inducing labour on perinatal mortality**. Component studies in French 2001 showing impact on stillbirths/perinatal mortality. Click here for file

Additional file 11 **Web Table 11. Component studies in Boulvain et al. 2008 meta-analysis: Impact of intracervical prostaglandin for labour induction on perinatal mortality**. Component studies in Boulvain et al. 2008 meta-analysis showing impact on stillbirths/perinatal mortality. Click here for file

Additional file 12 **Web Table 12. Component studies in Kelly et al. 2003 meta-analysis: Impact of vaginal prostaglandin (prostaglandin E2 and PGF2α) for term labour induction on perinatal mortality**. Component studies in Kelly et al. 2003 meta-analysis showing impact on stillbirths/perinatal mortality. Click here for file

Additional file 13 **Web Table 13. Component studies in Hutton and Mozurkewich 2001: Impact of extra-amniotic prostaglandin for labour induction on perinatal mortality**. Component studies in Hutton **and Mozurkewich** 2001 meta-analysis showing impact on stillbirths/perinatal mortality. Click here for file

Additional file 14 **Web Table 14. Component studies in Luckas et al. 2000 meta-analysis: Impact of intravenous prostaglandin for induction of labour on perinatal mortality**. Component studies in Luckas et al. 2000 meta-analysis showing impact on stillbirths/perinatal mortality. Click here for file

Additional file 15 **Web Table 15. Component studies in Neilson 2000 meta-analysis: Impact of mifepristone for labour induction on perinatal mortality**. Component studies in Neilson 2000 meta-analysis showing impact on stillbirths/perinatal mortality. Click here for file

Additional file 16 **Web Table 16. Component studies in Hofmeyr and Hannah 2003 meta-analysis: Impact of planned Caesarean section for term breech on perinatal/neonatal mortality**. Component studies in Hofmeyr **and Hannah ** 2003 meta-analysis showing impact on stillbirths/perinatal mortality. Click here for file

Additional file 17 **Web Table 17. Component studies in Duley et al. 2003 meta-analysis: Impact of magnesium sulphate and other anti-convulsants for pre-eclampsia on stillbirth and perinatal mortality**. Component studies in Duley et al. 2003 meta-analysis showing impact on stillbirths/perinatal mortality. Click here for file

Additional file 18 **Web Table 18. Component studies in Duley and Henderson-Smart 2003 meta-analysis: Impact of magnesium sulphate versus diazepam for eclampsia on stillbirth and perinatal mortality**. Component studies in Duley **and Henderson-Smart **2003 meta-analysis showing impact on stillbirths/perinatal mortality. Click here for file

Additional file 19 **Web Table 19. Component studies in Duley and Henderson-Smart 2003 meta-analysis: Impact of magnesium sulphate versus phenytoin for eclampsia on stillbirth and perinatal mortality**. Component studies in Duley and **Henderson-Smart** 2003 meta-analysis showing impact on stillbirths/perinatal mortality. Click here for file

Additional file 20 **Web Table 20. Component studies in Duley and Gulmezoglu 2000 meta-analysis: Impact of magnesium sulphate vs. lytic cocktail on stillbirth and neonatal mortality**. Component studies in **Duley and Gulmezoglu ** 2000 meta-analysis showing impact on stillbirths/perinatal mortality. Click here for file

Additional file 21 **Web Table 21. Component studies in Doyle et al. 2007 meta-analysis: Impact of magnesium sulphate as a neuroprotective agent in women at risk of pre-term birth on fetal death**. Component studies in Doyle et al. 2007 meta-analysis showing impact on stillbirths/perinatal mortality. Click here for file

Additional file 22 **Web Table 22. Component studies in Crowther et al. 2002 meta-analysis: Impact of magnesium sulphate in threatened pre-term labour on fetal deaths**. Component studies in Crowther et al. 2002 meta-analysis showing impact on stillbirths/perinatal mortality. Click here for file

Additional file 23 **Web Table 23. Component studies in Crowther and Moore 1998: Impact of magnesium maintenance therapy for preventing pre-term birth on perinatal mortality**. Component studies in Crowther and Moore 1998 showing impact on stillbirths/perinatal mortality. Click here for file

Additional file 24 **Web Table 24. Component studies in Say et al. 2003 meta-analysis: Impact of maternal oxygen therapy on perinatal mortality**. Component studies in Say et al. 2003 meta-analysis showing impact on stillbirths/perinatal mortality. Click here for file

Additional file 25 **Web Table 25. Component studies in Hofmeyr 1998 meta-analysis: Impact of amnioinfusion for potential or suspected umbilical cord compression on perinatal mortality**. Component studies in Hofmeyr 1998 meta-analysis showing impact on stillbirths/perinatal mortality. Click here for file

Additional file 26 **Web Table 26. Component studies in Hofmeyr 2002 meta-analysis: Impact of amnioinfusion for meconium-stained liquor on perinatal mortality**. Component studies in Hofmeyr 2002 meta-analysis showing impact on stillbirths/perinatal mortality. Click here for file
