# Supplementary material for: Reducing stillbirths: interventions during labour
Source: BMC Pregnancy Childbirth. 2009 May 7;9(Suppl 1):S6. doi: 10.1186/1471-2393-9-S1-S6 (PMC2679412; doi:10.1186/1471-2393-9-S1-S6)
Supplement: Additional file 1 — Web Table 1. Component studies in Johanson and Menon 1999 meta-analysis: Impact of vacuum vs. forceps delivery on perinatal mortality. Component studies in Johanson and Menon 1999 meta-analysis showing impact on stillbirths/perinatal mortality. [file 1471-2393-9-S1-S6-S1.doc]

**Web Table 1. Component studies in Johanson and Menon 1999 [1] meta-analysis: Impact of vacuum vs. forceps delivery on perinatal mortality**

| **Source** | **Location and Type of Study** | **Intervention** | **Stillbirths / Perinatal Outcomes** |
| --- | --- | --- | --- |
| 1. Dell et al. 1985 [2] | USA (New Orleans). Louisiana State University.  RCT. N=118 nulliparous patients [N=73 vacuum group (N=37 Mityvac, N=36 Silastic), N=45 forceps]. | Compared the impact on perinatal mortality of vacuum (study group) vs. Tucker-McLane forceps delivery (controls). | PMR: OR=not estimable. [0/73 vs. 0/45 in the study vs. control groups, respectively]. |
| 2. Ehlers et al. 1974 [3] | Denmark.  RCT. N=206 participants (N=107 vacuum group, N=99 forceps). | Compared the impact on perinatal mortality of vacuum (study group) vs. forceps delivery (controls). | PMR: OR=not estimable.  [0/107 vs. 0/112 in vacuum vs. forceps groups, respectively]. |
| 3. Fall et al. 1986 [4] | Sweden.  RCT. N=36 participants (N=20 vacuum group, N=16 forceps). | Assessed the effect on perinatal mortality of vacuum (study group) vs. forceps delivery (controls). | PMR: OR=not estimable.  [0/20 vs. 0/16 in vacuum and forceps groups, respectively]. |
| 4. Johanson et al. 1993 [5, 6] | England (Keele, West Midlands). District general hospitals (secondary setting).  Non-blinded RCT. N=607 participants [N=296 vacuum group (Silc-cup - 177, OA metal - 95, OP metal - 23, VE not used - 1); N=311 forceps (Neville Barnes - 258, Kjellands - 44, Manual rotation - 5, Lift Out - 0, Forceps not used - 4)]. | Compared the impact on perinatal mortality of vacuum extraction (study group) vs. forceps delivery (controls). | PMR: OR=1.05 (95% CI: 0.07-16.85) [NS].  [1/296 vs. 1/311 in vacuum vs. forceps groups, respectively].  Stillbirth data not given. |
| 5. Lasbrey et al. 1964 [7] | South Africa (Durban).  Non-blinded RCT. N=252 participants (N=121 vacuum, N=131 forceps). | Compared the impact on perinatal mortality of Malmstrom vacuum extractor (study group) vs. forceps delivery (controls). | PMR: OR=0.39 (95% CI: 0.05-2.83) [NS].  [1/121 vs. 3/131 in vacuum vs. forceps groups, respectively].  Fresh SBR: 1/121 vs. 1/131 in study vs. control groups, respectively. |
| 6. Vacca et al. [8-11] | England (Portsmouth).  Non-blinded RCT. N=304 single, vertex pregnancies (N=152 vacuum group, N=152 forceps). | Compared the impact on perinatal mortality of 50mm anterior and posterior Bird vacuum extractor cups (study group) vs. Haig Ferguson's and Kjellands forceps (controls). | PMR: OR=7.39 (95% CI: 0.15-372.38) [NS].  [1/152 vs. 0/152 in study and control groups, respectively]. |
| 7. Johanson et al. 1989 [12, 13] | England (Wigan). North Staffordshire and Billinge Maternity Hospital.  RCT. N=264 single, cephalic pregnancies (N=132 ventouse group, N=132 forceps). | Compared the impact on perinatal mortality of 'Silc cup' ventouse (study group) vs. forceps delivery (controls). | PMR: OR=not estimable.  [0/132 in both the groups]. |

**References**

1. Johanson RB, Menon V: **Vacuum extraction versus forceps for assisted vaginal delivery**. *Cochrane Database of Systematic Reviews;* 1999(2):CD000224.

2. Dell DL, Sightler SE, Plauche WC: **Soft cup vacuum extraction: a comparison of outlet delivery**. *Obstet Gynecol* 1985, **66**(5):624-628.

3. Ehlers N, Jensen IK, Hansen KB: **Retinal haemorrhages in the newborn. Comparison of delivery by forceps and by vacuum extractor**. *Acta Ophthalmol (Copenh)* 1974, **52**(1):73-82.

4. Fall O, Ryden G, Finnstrom K, Finnstrom O, Leijon I: **Forceps or vacuum extraction? A comparison of effects on the newborn infant**. *Acta Obstet Gynecol Scand* 1986, **65**(1):75-80.

5. Johanson RB, Wilkinson P, Bastible A, Ryan S, Murphy H, Redman CWE, O'Brien PMS: **Health after assisted vaginal delivery; follow-up of a random controlled study**. *J Obstet Gynaecol;* 1993, **13**:242-246.

6. Johanson RB, Rice C, Doyle M, Arthur J, Anyanwu L, Ibrahim J, Warwick A, Redman CW, O'Brien PM: **A randomised prospective study comparing the new vacuum extractor policy with forceps delivery**. *Br J Obstet Gynaecol* 1993, **100**(6):524-530.

7. Lasbrey AH, Orchard CD, Crichton D: **A study of the relative merits and scope for vacuum extraction as opposed to forceps delivery**. *S Afr J Obstet Gynaecol;* 1964, **2**:1-3.

8. Carmody F, Grant A, Mutch L, Vacca A, Chalmers I: **Follow up of babies delivered in a randomized controlled comparison of vacuum extraction and forceps delivery**. *Acta Obstet Gynecol Scand* 1986, **65**(7):763-766.

9. Garcia J, Anderson J, Vacca A, Elbourne DR, Grant AM, Chalmers I: **Views of women and their medical and midwifery attendants about instrument delivery using vacuum extraction and forceps**. *J Psychosom Obstet Gynaecol;* 1985, **4**:1-9.

10. Vacca A, Grant AM: **Portsmouth operative delivery trial. A randomised controlled trial to compare vacuum extraction with forceps delivery**. *Eur J Obstet Gynecol Reprod Biol;* 1983, **15**:305-309.

11. Vacca A, Grant A, Wyatt G, Chalmers I: **Portsmouth operative delivery trial: a comparison vacuum extraction and forceps delivery**. *Br J Obstet Gynaecol* 1983, **90**(12):1107-1112.

12. Johanson R, Pusey J, Livera N, Jones P: **North Staffordshire/Wigan assisted delivery trial**. *Br J Obstet Gynaecol* 1989, **96**(5):537-544.

13. Pusey J, Hodge C, Wilkinson P, Johanson R: **Maternal impressions of forceps or the Silc-cup**. *Br J Obstet Gynaecol* 1991, **98**(5):487-488.
