# Supplementary material for: Reducing stillbirths: interventions during labour
Source: BMC Pregnancy Childbirth. 2009 May 7;9(Suppl 1):S6. doi: 10.1186/1471-2393-9-S1-S6 (PMC2679412; doi:10.1186/1471-2393-9-S1-S6)
Supplement: Additional file 2 — Web Table 2. Component studies in Johanson and Menon 2000: Impact of soft vs. rigid cup delivery on perinatal mortality. Component studies in Johanson and Menon 2000 meta-analysis showing impact on stillbirths/perinatal mortality. [file 1471-2393-9-S1-S6-S2.doc]

**Web Table 2. Component studies in Johanson and Menon 2000 [1]: Impact of soft vs. rigid cup delivery on perinatal mortality**

| **Source** | **Location and Type of Study** | **Intervention** | **Stillbirths / Perinatal Outcomes** |
| --- | --- | --- | --- |
| 1. Lee 1996 [2] | Malaysia (Kuala Lumpur). General Hospital, Jalan Pahang.  RCT. N=72 singleton, vertex pregnancies (N=32 intervention group, N=40 controls). | Compared the impact on death rate of soft silicone cup (intervention) vs. rigid metal cup (controls). | Death rate: OR=1.26 (95% CI: 0.08-20.85) **[NS]**.  [1/32 vs. 1/40 in the soft vs. rigid cups, respectively]. |

**References**

1. Johanson R, Menon V: **Soft versus rigid vacuum extractor cups for assisted vaginal delivery**. *Cochrane Database Syst Rev* 2000(2):CD000446.

2. Lee HY, Subramaniam N, Nordin MM: **Vacuum delivery at The Maternity Hospital Kuala Lumpur: a comparison of metal and silicone cups**. *Singapore Med J* 1996, **37**(1):55-60.
