# Supplementary material for: Reducing stillbirths: interventions during labour
Source: BMC Pregnancy Childbirth. 2009 May 7;9(Suppl 1):S6. doi: 10.1186/1471-2393-9-S1-S6 (PMC2679412; doi:10.1186/1471-2393-9-S1-S6)
Supplement: Additional file 3 — Web Table 3. Component studies in Gulmezoglu et al. 2006 meta-analysis: Impact of induction of labour at or beyond term by cervical status on stillbirth and perinatal mortality. Component studies in Gulmezoglu et al. 2006 meta-analysis showing impact on stillbirths/perinatal mortality. [file 1471-2393-9-S1-S6-S3.doc]

**Web Table 3. Component studies in Gulmezoglu et al. 2006 [1] meta-analysis: Impact of induction of labour at or beyond term by cervical status on stillbirth and perinatal mortality.**

| **Source** | **Location and Type of Study** | **Intervention** | **Stillbirths / Perinatal Outcomes** |
| --- | --- | --- | --- |
| **Cervix favourable** | | | |
| 1. Chanrachkul 2003 [2] | Thailand (Bangkok). Tertiary care setting.  RCT. N=250 women of gestational age: 41 + weeks. | Compared the impact of intervention with amniotomy + oxytocin (if uterine contractions inadequate after 2 hours). In the control group, spontaneous labour was awaited unless 1) nonreactive NST or 2) amniotic fluid index < 5 cm or 3) medical or obstetric indication for birth or 4) reaching 44 completed weeks. | SBR: RR not estimable.  [0/124 vs. 0/125 in intervention and control groups, respectively].  PMR: RR not estimable.  [0/124 vs. 0/125 in intervention and control groups, respectively]. |
| **Cervix unfavourable** | | | |
| 2. Dyson 1987 [3] | USA (California). Kaiser Permanente Medical Care Hospital.  RCT. N=302 low-risk women at 41 completed weeks of gestation. | Compared the impact on pregnancy outcomes of intervention with prostaglandin E2 gel (initially 3 mg but later reduced to 0.5 mg). If no labour in 24 hours, repeat prostaglandin E2 and oxytocin if needed. The control group had NST twice weekly, pelvic examination and amniotic fluid determination weekly between 41-42 weeks and twice weekly afterwards. | SBR: RR not estimable.  [0/152 vs. 0/150 in intervention and control groups, respectively].  PMR: RR=0.33 (95% CI: 0.01-8.01) **[NS]**.  [0/152 vs. 1/150 in intervention and control groups, respectively]. |
| 3. Gelisen 2005 [4] | Turkey (Ankara). Teaching hospital.  RCT. N=600 low-risk women at 41 completed weeks of gestational age. | Compared the impact of labour induction in 3 groups: misoprostol vaginally 50 mcg or oxytocin infusion initially at 1 mU/min or Foley catheter (intervention). Membrane sweeping was performed in more than 90% women in the induction groups. The expectant management group continued until 42 completed weeks with twice-weekly amniotic fluid index and NST and one biophysical profile measurement. | SBR: RR=0.33 (95% CI: 0.01-8.15) **[NS]**.  [0/300 vs. 1/300 in intervention and control groups, respectively].  PMR: RR=0.33 (95% CI: 0.01-8.15) **[NS]**.  [0/300 vs. 1/300 in intervention and control groups, respectively]. |
| 4. Herabutya 1992 [5] | Thailand (Bangkok). Tertiary care setting.  RCT. N=108 low-risk women at 42 completed weeks of gestation. | Assessed the effects of intervention with prostaglandin E2 intracervical, repeated after 6 hours, amniotomy and oxytocin on day 2 according to contractions. The control group had weekly NST. | SBR: RR not estimable.  [0/57 vs. 0/51 in intervention and control groups, respectively].  PMR: RR=0.30 (95% CI: 0.01-7.18) **[NS]**.  [0/57 vs. 1/51 in intervention and control groups, respectively]. |
| 5. Martin 1989 [6] | USA (Jackson).  RCT. N=22 low-risk women at 41 completed gestational weeks. | Compared the effects on pregnancy outcome of intervention with laminaria tents followed by oxytocin. The control group had weekly ultrasound for amniotic fluid assessment and NST. | SBR: RR not estimable.  [0/12 vs. 0/10 in intervention and control groups, respectively].  PMR: RR not estimable.  [0/12 vs. 0/10 in intervention and control groups, respectively]. |
| 6. NICHHD 1994 [7] | USA. University hospitals.  RCT. N=440 low-risk women at 41 completed weeks of gestation. | Compared the impact of intervention of 1) cervical priming with prostaglandin E2 gel followed 12 hours later with oxytocin; or 2) no cervical priming (placebo gel) followed 12 hours later with oxytocin. The control group had weekly cervix assessments, twice weekly NST and amniotic fluid volume assessment. | SBR: RR not estimable.  [0/174 vs. 0/175 in intervention and control groups, respectively].  PMR: RR not estimable.  [0/174 vs. 0/175 in intervention and control groups, respectively]. |
| **Not mentioned/not separated** | | | |
| 7. Augensen 1987 [8] | Norway (Bergen). Tertiary care.  RCT. N=409 healthy women with singleton pregnancy and certain dates. | Compared the impact on perinatal mortality of intervention with immediate induction with oxytocin (5 IU increased in a stepwise manner). The control group had NST every 3-4 days, induction of labour (IOL) after 7 days. | SBR: RR not estimable.  [0/214 vs. 0/195 in intervention and control groups, respectively].  PMR: RR not estimable.  [0/214 vs. 0/195 in intervention and control groups, respectively]. |
| 8. Bergsjo 1989 [9] | China (Wuhan). Hubei Maternity and Child Health Hospital.  RCT. N=188 women at 42 completed weeks of gestation. | Compared the impact of intervention involving stripping of membranes followed by oxytocin infusion and AROM if cervix sufficiently dilated. The control group had no intervention for one week, IOL at 43 weeks. | SBR: RR not estimable.  [0/94 vs. 0/94 in intervention and control groups, respectively].  PMR: RR=0.50 (95% CI: 0.05-5.42) **[NS]**.  [1/94 vs. 2/94 in intervention and control groups, respectively]. |
| 9. Hannah 1992 [10] | Canada. Tertiary care (22 hospitals).  RCT. N=3418 low-risk women at 41 completed weeks of gestation. | Compared the impact on pregnancy outcomes of intervention involving IOL within 4 days of randomisation, first with prostaglandins and then with oxytocin if necessary. The control group had daily fetal movement counting, NST and amniotic fluid measurement 2-3 times per week. | SBR: RR=0.20 (95% CI: 0.01-4.18) **[NS]**.  [0/1701 vs. 2/1706 in intervention and control groups, respectively].  PMR: RR=0.20 (95% CI: 0.01-4.18) **[NS]**.  [0/1701 vs. 2/1706 in intervention and control groups, respectively]. |
| 10. Henry 1969 [11] | UK (Birmingham).  RCT. N=112 low-risk women of 41+ weeks gestational age. | Compared the impact on pregnancy outcome of intervention with amniotomy and oxytocin. The control group had weekly amnioscopy. | SBR: RR=0.35 (95% CI: 0.01-8.30) **[NS]**.  [0/55 vs. 1/57 in intervention and control groups, respectively].  PMR: RR=0.21 (95% CI: 0.01-4.22) **[NS]**.  [0/55 vs. 2/57 in intervention and control groups, respectively]. |
| 11. James 2001 [12] | India (Vellore). Tertiary care setting.  RCT. N=74 low-risk women at 41 completed weeks of gestation. | Compared the impact on pregnancy outcome of intervention (depending on the cervix ripeness),either direct induction of labour or first ripening then induction of labour. The control group had daily fetal movement counts, biophysical profile every second day. | SBR: RR not estimable.  [0/37 vs. 0/37 in intervention and control groups, respectively].  PMR: RR not estimable.  [0/37 vs. 0/37 in intervention and control groups, respectively]. |
| 12. Suikkari 1983 [13] | Finland (Lappenranta).  RCT. N=119 women with regular menses at 41+ weeks of gestation. | Compared the impact on pregnancy outcome of intervention involving oxytocin alone or with amniotomy depending on the cervix. The control group had obstetric examination, NST, biochemical tests and amniotic fluid determination every 3 days. | SBR: RR not estimable.  [0/66 vs. 0/53 in intervention and control groups, respectively].  PMR: RR not estimable.  [0/66 vs. 0/53 in intervention and control groups, respectively]. |

**References**

1. Gulmezoglu AM, Crowther CA, Middleton P: **Induction of labour for improving birth outcomes for women at or beyond term**. *Cochrane Database Syst Rev* 2006(4):CD004945.

2. Chanrachkul B, Herabutya Y: **Postterm with favorable cervix: is induction necessary?** *European Journal of Obstetrics & Gynecology and Reproductive Biology;* 2003, **106**:154-157.

3. Dyson DC, Miller PD, Armstrong MA: **Management of prolonged pregnancy: induction of labor versus antepartum fetal testing**. *Am J Obstet Gynecol* 1987, **156**(4):928-934.

4. Gelisen O, Caliskan E, Dilbaz S, Ozdas E, Dilbaz B, Haberal A: **Induction of labor with three different techniques at 41 weeks of gestation or spontaneous follow-up until 42 weeks in women with definitely unfavorable cervical scores**. *Eur J Obstet Gynecol Reprod Biol* 2005, **120**(2):164-169.

5. Herabutya Y, Prasertsawat PO, Tongyai T, Isarangura Na Ayudthya N: **Prolonged pregnancy: the management dilemma**. *Int J Gynaecol Obstet* 1992, **37**(4):253-258.

6. Martin JN, Jr., Sessums JK, Howard P, Martin RW, Morrison JC: **Alternative approaches to the management of gravidas with prolonged-postterm-postdate pregnancies**. *J Miss State Med Assoc* 1989, **30**(4):105-111.

7. **A clinical trial of induction of labor versus expectant management in postterm pregnancy. The National Institute of Child Health and Human Development Network of Maternal-Fetal Medicine Units**. *Am J Obstet Gynecol* 1994, **170**(3):716-723.

8. Augensen K, Bergsjo P, Eikeland T, Askvik K, Carlsen J: **Randomised comparison of early versus late induction of labour in post-term pregnancy**. *Br Med J (Clin Res Ed)* 1987, **294**(6581):1192-1195.

9. Bergsjo P, Huang GD, Yu SQ, Gao ZZ, Bakketeig LS: **Comparison of induced versus non-induced labor in post-term pregnancy. A randomized prospective study**. *Acta Obstet Gynecol Scand* 1989, **68**(8):683-687.

10. Hannah ME, Hannah WJ, Hellmann J, Hewson S, Milner R, Willan A: **Induction of labor as compared with serial antenatal monitoring in post-term pregnancy. A randomized controlled trial. The Canadian Multicenter Post-term Pregnancy Trial Group**. *N Engl J Med* 1992, **326**(24):1587-1592.

11. Henry GR: **A controlled trial of surgical induction of labour and amnioscopy in the management of prolonged pregnancy**. *J Obstet Gynaecol Br Commonw* 1969, **76**(9):795-798.

12. James C, George SS, Gaunekar N, Seshadri L: **Management of prolonged pregnancy: a randomized trial of induction of labour and antepartum foetal monitoring**. *Natl Med J India* 2001, **14**(5):270-273.

13. Suikkari AM, Jalkanen M, Heiskala H, Koskela O: **Prolonged pregnancy: induction or observation**. *Acta Obstetricia et Gynecologica Scandinavica Supplement;* 1983, **116**:58.
