# Supplementary material for: Reducing stillbirths: interventions during labour
Source: BMC Pregnancy Childbirth. 2009 May 7;9(Suppl 1):S6. doi: 10.1186/1471-2393-9-S1-S6 (PMC2679412; doi:10.1186/1471-2393-9-S1-S6)
Supplement: Additional file 4 — Web Table 4. Component studies in Irion and Boulvain 1998 meta-analysis: Impact of induction of labour for suspected fetal macrosomia on perinatal mortality. Component studies in Irion and Boulvain 1998 meta-analysis showing impact on stillbirths/perinatal mortality. [file 1471-2393-9-S1-S6-S4.doc]

**Web Table 4. Component studies in Irion and Boulvain 1998 [1] meta-analysis : Impact of induction of labour for suspected fetal macrosomia on perinatal mortality.**

| **Source** | **Location and Type of Study** | **Intervention** | **Stillbirths / Perinatal Outcomes** |
| --- | --- | --- | --- |
| 1. Kean 1998 [2] | Unknown location (unpublished).  RCT. N=59 women (N=30 intervention group, N=29 controls). | Compared the impact on perinatal mortality of immediate induction of labour using either oxytocin or prostaglandins (intervention) vs. women in the expectant management group where labour was induced at 42 completed weeks of gestation (controls). | PMR: RR not estimable.  [0/30 vs. 0/29 in intervention and control groups, respectively]. |
| 2. Tey 1995 [3] | USA.  RCT. N=40 non-diabetic pregnant women at 37 to 42 weeks, with an ultrasound estimated fetal weight between 4000 and 4750 g. | Compared the impact of induction of labour with prostaglandins E2 gel if the cervical status was unfavourable (Bishop score < 6), followed by oxytocin infusion (intervention) vs. expectant management (controls). | PMR: RR not estimable.  [0/19 vs. 0/21 in intervention and control groups, respectively]. |

**References**
