# Supplementary material for: Reducing stillbirths: interventions during labour
Source: BMC Pregnancy Childbirth. 2009 May 7;9(Suppl 1):S6. doi: 10.1186/1471-2393-9-S1-S6 (PMC2679412; doi:10.1186/1471-2393-9-S1-S6)
Supplement: Additional file 5 — Web Table 5. Component studies in Boulvain et al. 2001: Impact of elective delivery in term diabetic pregnant women on perinatal mortality. Component studies in Boulvain et al. 2001 meta-analysis showing impact on stillbirths/perinatal mortality. [file 1471-2393-9-S1-S6-S5.doc]

**Web Table 5. Component studies in Boulvain et al. 2001 [1]: Impact of elective delivery in term diabetic pregnant women on perinatal mortality**

| **Source** | **Location and Type of Study** | **Intervention** | **Stillbirths / Perinatal Outcomes** |
| --- | --- | --- | --- |
| Kjos et al. 1993 [2] | United States of America  Tertiary care setting.  RCT. N=200 women (N=100 intervention group, N=100 controls). | Assessed the impact of intervention consisting of active induction of labour by IV oxytocin within 5 days. For pregnancies with unclear gestational age, amniocentesis was performed, and induction delayed until lecithin/sphingomyelin (L/S) ratio >= 2.0. The control group was subjected to expectant management until 42 weeks with twice weekly antenatal testing and weekly consultation. Induction of labour indicated by fetal distress, preeclampsia, poor metabolic control, estimated fetal weight >4200 g, or term >42 weeks (294 days). | PMR: RR not estimable.  [0/100 in both groups]. |

**References**

**1. Boulvain M, Stan C, Irion O: Elective delivery in diabetic pregnant women. *Cochrane Database Syst Rev* 2001(2):CD001997.**

**2. Kjos SL, Henry OA, Montoro M, Buchanan TA, Mestman JH: Insulin-requiring diabetes in pregnancy: a randomized trial of active induction of labor and expectant management. *Am J Obstet Gynecol* 1993, 169(3):611-615.**
