# Supplementary material for: Reducing stillbirths: interventions during labour
Source: BMC Pregnancy Childbirth. 2009 May 7;9(Suppl 1):S6. doi: 10.1186/1471-2393-9-S1-S6 (PMC2679412; doi:10.1186/1471-2393-9-S1-S6)
Supplement: Additional file 6 — Web Table 6. Component studies in Dare et al. 2006 meta-analysis: Impact of planned early birth on perinatal mortality. Component studies in Dare et al. 2006 meta-analysis showing impact on stillbirths/perinatal mortality. [file 1471-2393-9-S1-S6-S6.doc]

**Web Table 6. Component studies in Dare et al. 2006 [1]meta-analysis: Impact of planned early birth on perinatal mortality**

| **Source** | **Location and Type of Study** | **Intervention** | **Stillbirths / Perinatal Outcomes** |
| --- | --- | --- | --- |
| ***Oxytocin as the mode of induction*** | | | |
| 1. Hannah 1996 [2] | Canada (Toronto). 72 hospitals in 6 countries (tertiary care setting).  RCT. N=2521 women (N=1258 intervention group, N=1263 controls). | Compared the impact on perinatal mortality of planned management of immediate induction of labour with intravenous oxytocin (intervention) vs. expectant management for up to four days, then induced with intravenous oxytocin if spontaneous labour had not occurred (controls). | Fetal death/PMR: OR=0.50 (95% CI: 0.09-2.74) **[NS]**.  [2/1258 vs. 4/1263 in intervention and control groups, respectively]. |
| 2. Mc Queen 1992 [3] | RCT. N=40 women (N=20 intervention group, N=20 controls). | Compared the impact of planned management with oxytocin infusion (intervention) vs. expectant management where if in labour, managed in same way as planned management, or observed until contractions; or if sepsis suspected woman was given antibiotics and induced with oxytocin (controls). | Fetal death/PMR: OR=0.32 (95% CI: 0.01-8.26) **[NS]**.  [0/20 vs. 1/20 in intervention and control groups, respectively]. |
| 3. Ottervanger 1996 [4] | Netherlands. Leyenburg Hospital.  RCT. N=123 women (N=61 intervention group, N=62 controls). | Compared the impact of planned management consisting of intravenous oxytocin, starting at a dose of 2.5 mU/min and augmented every 20 mins until adequate contractility was obtained (intervention) vs. expectant management with admission to hospital for 48 hours; if labour had not ensued within 48 hours, women were offered induction of labour by intravenous oxytocin (controls). | Fetal death/PMR: OR not estimable.  [0/61 vs. 0/62 in intervention and control groups, respectively]. |
| 4. Shalev 1995 [5] | Israel (Afula). Central Emek Hospital.  Prospective nonrandomised trial. N=566 women (N=298 intervention group, N=268 controls). | Compared the impact of planned management consisting of 12 hour expectant management, then oxytocin (intervention) vs. expectant management of 72 hours (controls). | Fetal death/PMR: OR not estimable.  [0/298 vs. 0/268 in intervention and control groups, respectively]. |
| ***Prostaglandin as the mode of induction*** | | | |
| 5. Hannah 1996 [2] | Canada (Toronto). 72 hospitals in 6 countries (tertiary care setting).  RCT. N=2520 women (N=1259 intervention group, N=1261 controls). | Compared the impact on perinatal mortality of planned management of immediate induction of labour with vaginal prostaglandin E2 gel (intervention) vs. expectant management for up to four days, then induced with vaginal prostaglandin E2 gel if spontaneous labour had not occurred (controls). | Fetal death/PMR: OR=0.50 (95% CI: 0.05-5.53) **[NS]**.  [1/1259 vs. 2/1261 in intervention and control groups, respectively]. |
| 6. Mahmood 1995 [6] | Scotland. Aberdeen Maternity Hospital.  RCT. N=100 parous women (N=50 intervention group, N=50 controls). | Compared the impact of planned management that included prostaglandin E2 gel, 1 mg administered at admission to posterior fornix and repeated 6 hours later if labour was not established (intervention) vs. expectant, conservative management (for up to 24 hours).  Both groups received intravenous oxytocin if labour did not start within 24 hours of admission using an escalating scale of 1-32 µ/min. | Fetal death/PMR: OR not estimable.  [0/50 in both the groups]. |

**References**

1. Dare MR, Middleton P, Crowther CA, Flenady VJ, Varatharaju B: **Planned early birth versus expectant management (waiting) for prelabour rupture of membranes at term (37 weeks or more)**. *Cochrane Database Syst Rev* 2006(1):CD005302.

2. Hannah ME, Ohlsson A, Farine D, Hewson SA, Hodnett ED, Myhr TL, Wang EE, Weston JA, Willan AR: **Induction of labor compared with expectant management for prelabor rupture of the membranes at term. TERMPROM Study Group**. *N Engl J Med* 1996, **334**(16):1005-1010.

3. McQueen D: **A randomized controlled trial comparing expectant management with active management in early rupture of the membranes**. In*.*; Personal communication 1992.

4. Ottervanger HP, Keirse MJ, Smit W, Holm JP: **Controlled comparison of induction versus expectant care for prelabor rupture of the membranes at term**. *J Perinat Med* 1996, **24**(3):237-242.

5. Shalev E, Peleg D, Eliyahu S, Nahum Z: **Comparison of 12- and 72-hour expectant management of premature rupture of membranes in term pregnancies**. *Obstet Gynecol* 1995, **85**(5 Pt 1):766-768.

6. Mahmood TA, Dick MJ: **A randomized trial of management of pre-labor rupture of membranes at term in multiparous women using vaginal prostaglandin gel**. *Obstet Gynecol* 1995, **85**(1):71-74.
