# Supplementary material for: Reducing stillbirths: interventions during labour
Source: BMC Pregnancy Childbirth. 2009 May 7;9(Suppl 1):S6. doi: 10.1186/1471-2393-9-S1-S6 (PMC2679412; doi:10.1186/1471-2393-9-S1-S6)
Supplement: Additional file 7 — Web Table 7. Component studies in Dodd and Crowther 2003: Impact of elective delivery of women with a twin pregnancy from term on perinatal mortality. Component studies in Dodd and Crowther 2003 meta-analysis showing impact on stillbirths/perinatal mortality. [file 1471-2393-9-S1-S6-S7.doc]

**Web Table 7. Component studies in Dodd and Crowther 2003 [1]: Impact of elective delivery of women with a twin pregnancy from term on perinatal mortality.**

| **Source** | **Location and Type of Study** | **Intervention** | **Stillbirths / Perinatal Outcomes** |
| --- | --- | --- | --- |
| 1. Suzuki 2000 [2] | Japan (Tokyo). Tertiary care setting.  RCT. N=36 women with a twin pregnancy at 37 weeks gestation, with the first twin in a cephalic presentation. | Compared the impact on perinatal mortality of induction of labour at 37 weeks gestation with vaginal prostaglandin E2 gel followed by ARM and oxytocin infusion as required (intervention) vs. expectant management with continued pregnancy surveillance until spontaneous onset of labor, provided no antenatal complications developed and fetal well-being confirmed (involved daily CTG and twice weekly ultrasound examination) (controls). | PMR: RR not estimable.  [0/34 vs. 0/38 in intervention and control groups, respectively]. |

**References**

1. Dodd JM, Crowther CA: **Elective delivery of women with a twin pregnancy from 37 weeks' gestation**. *Cochrane Database Syst Rev* 2003(1):CD003582.

2. Suzuki S, Otsubo Y, Sawa R, Yoneyama Y, Araki T: **Clinical trial of induction of labor versus expectant management in twin pregnancy**. *Gynecol Obstet Invest* 2000, **49**(1):24-27.
