# Supplementary material for: Reducing stillbirths: interventions during labour
Source: BMC Pregnancy Childbirth. 2009 May 7;9(Suppl 1):S6. doi: 10.1186/1471-2393-9-S1-S6 (PMC2679412; doi:10.1186/1471-2393-9-S1-S6)
Supplement: Additional file 8 — Web Table 8. Component studies in Alfirevic and Weeks 2006 meta-analysis: Impact of oral misoprostol for induction of labour on perinatal mortality. Component studies in Alfirevic and Weeks 2006 meta-analysis showing impact on stillbirths/perinatal mortality. [file 1471-2393-9-S1-S6-S8.doc]

**Web Table 8. Component studies in Alfirevic and Weeks 2006 meta-analysis [1]: Impact of oral misoprostol for induction of labour on perinatal mortality**

| **Source** | **Location and Type of Study** | **Intervention** | **Stillbirths / Perinatal Outcomes** |
| --- | --- | --- | --- |
| **Oral misoprostol versus placebo** | | | |
| 1. Ngai 1996 [2, 3] | Hong Kong.  RCT. N=82 women with a singleton pregnancy at term and pre-labour spontaneous rupture of membranes confirmed by speculum examination. All women had a reactive non-stress test on admission. | Compared the impact on perinatal mortality of 200 mcg oral misoprostol powder (intervention) vs. placebo (vitamin B6) (controls).  If no response after 12 hours labour was induced with oxytocin. | PMR: RR not estimable.  [0/39 vs. 0/41 in intervention and control groups, respectively]. |
| **Oral misoprostol versus vaginal prostaglandin E2** | | | |
| 2. Dallenbach et al. [4] | Switzerland.  RCT. N=202 women with healthy fetuses at term and with unfavourable cervixes (Bishop score </= 6). | Compared the impact of titrated oral misoprostol (20 mcg every 2 hrs x 2 then 40 mcg every 2 hrs x 10 until 3 contractions every 10 mins, max dose 475 mcg) vs. prostaglandin E2 gel 2 mg 6 hours apart. | PMR: RR not estimable.  [0/100 in both the groups]. |
| 3. Hofmeyr et al. 2001.[5] | UK (Liverpool) and South Africa. Academic hospitals.  RCT. N=695 women in whom the decision has been made to induce labour with prostaglandin E2 regardless of membrane and cervical status. | Compared the impact of titrated oral misoprostol (intervention) vs. vaginal prostaglandin E2 2 mg (controls).  Oral misoprostol was administered as solution (200 mcg tablet dissolved in 200 mls of water). Initial 2-3 doses were 20 mcg increased to 40 mcg every 2 hours. Further doses were not given if contractions were judged to be clinically adequate. Vaginal prostaglandin E2 was given as a 2 mg gel followed by another dose 6 hours later. In both groups oxytocin was started if there was no response after 24 hours. | PMR: RR=1.00 (95% CI: 0.06-15.97) **[NS]**.  [1/345 vs. 1/346 in intervention and control groups, respectively]. |
| 4. Matonhodze et al. 2003 [6] | South Africa (Johannesburg). Academic hospitals.  RCT. N=526 women with singleton pregnancies of over 34 weeks and intact membranes. | Compared the impact of labour induction with Foley catheter with 50 ml bulb for 24 hours followed by titrated oral misoprostol if not in labour [intervention] vs. titrated oral misoprostol 20 mcg 2 hourly x 3 then 40 mcg 2-hourly (continued in labour if contractions slowed)[controls] | PMR: RR=0.33 (95% CI: 0.01-8.03) **[NS]**.  [0/176 vs. 1/174 in intervention and control groups, respectively]. |
| 5. Tessier 1997 [7] | Canada.  RCT. N=267 women with an indication for induction of labour (N=135 intervention group, N=132 controls). | Compared the impact of 50 micrograms of oral misoprostol (intervention) every 6 hours for maximum of 4 doses vs. vaginal prostaglandin E2 gel (2 mg) (controls). Each woman received also a placebo gel or tablet. | PMR: RR not estimable.  [0/135 vs. 0/132 in intervention and control groups, respectively]. |
| **Oral misoprostol versus intracervical prostaglandin E2** | | | |
| 6. Bartha et al. 2000. [8] | Spain.  RCT. N=200 women with intact membranes and unfavourable cervix (Bishop score < 6). | Compared the impact of 200 mcg of oral misoprostol as a single dose (intervention) vs. 0.5 mg prostaglandin E2 intracervically every 6 hours (maximum 4 doses) (controls). | PMR: RR not estimable.  [0/100 in both the groups]. |
| 7. Langenegger et al. 2005 [9] | South Africa.  RCT. N=200 women with "indications for induction" at over 34 weeks with intact membranes. | Compared the impact of oral misoprostol 50 mcg 4 hourly (max x 6) (intervention) or intracervical prostaglandin E2 0.5 mg 6 hourly (max x 4) (controls). Dosages could be repeated after a 24 hr rest period. | PMR: RR not estimable.  [0/96 vs. 0/95 in intervention and control groups, respectively]. |
| **Oral misoprostol versus oxytocin** | | | |
| 8. Butt 1999 [10] | Canada.  RCT. N=108 women with PROM at term (N=72 women were nulliparous and N=57 had Bishop score < 7). | Compared the impact of oral misoprostol 50 mcg every 4 hours (intervention) vs. intravenous oxytocin (controls). | PMR: RR not estimable.  [0/55 vs. 0/53 in intervention and control groups, respectively]. |
| 9. Mozurkewich 2003 [11] | USA. Multicentred (10 centers).  RCT. N=305 women. | Compared the impact of oral misoprostol 100 mcg 6-hourly x 2 followed by iv oxytocin (intervention) vs. immediate iv oxytocin (controls). | PMR: RR=2.76 (95% CI: 0.11-67.13) **[NS]**.  [1/159 vs. 0/146 in intervention and control groups, respectively]. |
| 10. Ngai 2000 [12] | China (Hong Kong). Queen Mary Hospital.  RCT. N=86 women with term PROM not in labour after 12 hours. | Compared the impact of oral misoprostol 100 mcg every 4 hours (max 3 doses) (intervention) vs. intravenous oxytocin (controls). | PMR: RR not estimable.  [0/40 in both the groups]. |
| **Oral versus vaginal misoprostol** | | | |
| **Comparison # 1: 50 mcg of oral misoprostol** | | | |
| 11. Bennett 1998 [13, 14] | Canada.  RCT. N=206 women with intact membranes. | Compared the impact of 50 mcg oral tablet with vaginal placebo (intervention) vs. oral placebo with 50 mcg vaginal tablet. Medication was given every 4 hours. | PMR: RR not estimable.  [0/104 vs. 0/102 in intervention and control groups, respectively]. |
| 12. Fisher 2001 [15, 16] | Canada.  RCT. N=124 women with intact membranes at any gestation, all with bishop scores of less than 9. | Compared the impact of oral misoprostol 50 mcg every 3 hours for 48 hours (intervention) vs. vaginal misoprostol 50 mcg every 6 hours for 48 hours (controls). | PMR: RR not estimable.  [0/62 vs. 0/64 in intervention and control groups, respectively]. |
| 13. Shetty 2001 [17-20] | UK. Aberdeen Maternity Hospital.  RCT. N=245 women at term with Bishop scores less than 8 (N=149 were nulliparous, N=116 had BS < 4). | Compared the impact of oral (intervention) vs. vaginal misoprostol (controls) 50 mcg 4 hourly (max 5 doses). | PMR: RR not estimable.  [0/122 vs. 0/123 in intervention and control groups, respectively]. |
| 14. Wing 1999 [21] | USA.  RCT. N=220 women with intact membranes and unfavourable cervix. | Compared the impact of oral misoprostol given 50 mcg every 4 hours to a maximum dose of 300 mcg (intervention) vs. vaginal misoprostol given 25 mcg every four hours to a maximum dose of 150 mcg (controls). | PMR: RR not estimable.  [0/110 vs. 0/110 in intervention and control groups, respectively]. |
| **Comparison # 2: 100 mcg of oral misoprostol** | | | |
| 15. Hall 2002 [22] | USA.  RCT. N=107 women at term with Bishop score < 5 (N=28 had ruptured membranes, N=69 were nulliparous). | Compared the impact of oral misoprostol 100 mcg followed after 3-4 hours by 200 mcg repeated every 3-4 hours until in labour (intervention) vs. vaginal misoprostol 25 mcg followed after 3-4 hours by 50 mcg repeated every 3-4 hours until in labour (controls). | PMR: RR not estimable.  [0/59 vs. 0/48 in intervention and control groups, respectively]. |
| **Comparison # 3: 200 mcg of oral misoprostol** | | | |
| 16. Adair 1998 [23, 24] | USA.  RCT. N=178 women with intact membranes and unfavourable cervix (Bishop score less than 7). | Compared the impact of oral misoprostol 200 micrograms and 1/2 tablet placebo vaginally (intervention) vs. oral placebo tablet and a 1/2 tablet of 100 micrograms misoprostol (50 micrograms) vaginally (controls). Doses were repeated every 6 hours (maximum 3) or until labour was established. | PMR: RR not estimable.  [0/93 vs. 0/85 in intervention and control groups, respectively]. |
| **Oral versus vaginal misoprostol among primiparae** | | | |
| 17. Toppozada 1997 [25] | Egypt.  RCT. N=40 women with singleton pregnancy and a live fetus at 37-42 weeks of gestation scheduled for induction of labour (N=20 intervention group, N=20 controls). | Compared the impact on perinatal mortality of oral misoprostol (100 micrograms). If there was no response within 3 hours, the majority of women were given 200 micrograms of oral misoprostol. The total permitted dose was 1000 mcg. The mean total dose was 510 mcg (SD=137.27 mcg). The control group was given vaginal misoprostol, initial dose was 100 mcg, followed by an assessment 3 hours later. If the response was judged to be adequate, additional 100 mcg were given every 3 hours until cervix was more than 5 cm dilated. If there was no response to the first vaginal tablet, another 100 mcg were given vaginally 3 hours later. If there was no response after the second dose, the third dose was doubled (200 mcg). Maximum permitted dose was 1000 mcg. The mean total dose in this group was 385 mcg (SD=142.44 mcg). | PMR: RR not estimable.  [0/7 vs. 0/9 in intervention and control groups, respectively]. |
| 18. Wing 1999 [21] | USA.  RCT. N=220 women with intact membranes and unfavourable cervix. | Compared the impact of oral misoprostol given 50 mcg every 4 hours to a maximum dose of 300 mcg (intervention) vs. vaginal misoprostol was given 25 mcg every four hours to a maximum dose of 150 mcg (controls). | PMR: RR not estimable.  [0/53 vs. 0/53 in intervention and control groups, respectively]. |

**References**

**1. Alfirevic Z, Weeks A: Oral misoprostol for induction of labour. *Cochrane Database Syst Rev* 2006(2):CD001338.**

**2. Ngai CSW, To WWK, Lao T, Ho PC: Cervical priming with oral misoprostol in prelabour rupture of membranes at term. In: *27th British Congress of Obstetrics and Gynaecology: 1995 July 4-7.; Dublin.*; 1995 July 4-7.: A479.**

**3. Ngai SW, To WK, Lao T, Ho PC: Cervical priming with oral misoprostol in pre-labor rupture of membranes at term. *Obstet Gynecol* 1996, 87(6):923-926.**

**4. Dallenbach P, Boulvain M, Viardot C, Irion O: Oral misoprostol or vaginal dinoprostone for labor induction: a randomized controlled trial. *Am J Obstet Gynecol* 2003, 188(1):162-167.**

**5. Hofmeyr GJ, Alfirevic Z, Matonhodze B, Brocklehurst P, Campbell E, Nikodem VC: Titrated oral misoprostol solution for induction of labour: a multi-centre, randomised trial. *BJOG* 2001, 108(9):952-959.**

**6. Matonhodze BB, Hofmeyr GJ, Levin J: Labour induction at term--a randomised trial comparing Foley catheter plus titrated oral misoprostol solution, titrated oral misoprostol solution alone, and dinoprostone. *S Afr Med J* 2003, 93(5):375-379.**

**7. Tessier F, Dansereau J: A double-blind randomized controlled trial comparing oral misoprostol to vaginal prostaglandin E2 gel for the induction of labour at or near term. *American Journal of Obstetrics and Gynecology;* 1997, 176:S111.**

**8. Bartha JL, Comino-Delgado R, Garcia-Benasach F, Martinez-Del-Fresno P, Moreno-Corral LJ: Oral misoprostol and intracervical dinoprostone for cervical ripening and labor induction: a randomized comparison. *Obstet Gynecol* 2000, 96(3):465-469.**

**9. Langenegger EJ, Odendaal HJ, Grove D: Oral misoprostol versus intracervical dinoprostone for induction of labor. *Int J Gynaecol Obstet* 2005, 88(3):242-248.**

**10. Butt KD, Bennett KA, Crane JM, Hutchens D, Young DC: Randomized comparison of oral misoprostol and oxytocin for labor induction in term prelabor membrane rupture. *Obstet Gynecol* 1999, 94(6):994-999.**

**11. Mozurkewich E, Horrocks J, Daley S, Von Oeyen P, Halvorson M, Johnson M, Zaretsky M, Tehranifar M, Bayer-Zwirello L, Robichaux A, 3rd *et al*: The MisoPROM study: a multicenter randomized comparison of oral misoprostol and oxytocin for premature rupture of membranes at term. *Am J Obstet Gynecol* 2003, 189(4):1026-1030.**

**12. Ngai SW, Chan YM, Lam SW, Lao TT: Labour characteristics and uterine activity: misoprostol compared with oxytocin in women at term with prelabour rupture of the membranes. *BJOG* 2000, 107(2):222-227.**

**13. Bennett K, Butt K, Crane J, Hutchens D, Young D: Misoprostol for labour induction at term. In: *Society of Obstetricians and Gynaecologists of Canada 54th Annual Meeting: 1998 June; Victoria, Canada.*; 1998 June.**

**14. Bennett KA, Butt K, Crane JM, Hutchens D, Young DC: A masked randomized comparison of oral and vaginal administration of misoprostol for labor induction. *Obstet Gynecol* 1998, 92(4 Pt 1):481-486.**

**15. Fisher S, Davies G, Mackenzie P: Oral versus vaginal misoprostol for induction of labour: a double-blind, placebo-controlled randomised trial. *American Journal of Obstetrics and Gynecology* 2001, 184:S117.**

**16. Fisher SA, Mackenzie VP, Davies GA: Oral versus vaginal misoprostol for induction of labor: a double-blind randomized controlled trial. *Am J Obstet Gynecol* 2001, 185(4):906-910.**

**17. Shetty A, Danielian P, Templeton A: A comparison of oral and vaginal misoprostol tablets in induction of labour at term. *BJOG* 2001, 108(3):238-243.**

**18. Shetty A, Danielian P, Templeton A: A comparison of oral and vaginal tablets in the induction of labor at term. In: *XVI FIGO World Congress of Obstetrics & Gynecology: 2000 Sept 3-8.; Washington DC, USA.*; 2000 Sept 3-8.: 28-29.**

**19. Shetty A, Danielian P, Templeton A: A comparison of oral and vaginal misoprostol in the induction of labour at term: a random allocation trial. *Journal of Obstetrics and Gynaecology;* 2000, 20:S19.**

**20. Shetty A, Danielian P, Templeton A: Oral versus vaginal misoprostol in the induction of labour at term: a randomised controlled trial. *BJOG: an international journal of obstetrics and gynaecology;* 2000, 107:813.**

**21. Wing DA, Ham D, Paul RH: A comparison of orally administered misoprostol with vaginally administered misoprostol for cervical ripening and labor induction. *Am J Obstet Gynecol* 1999, 180(5):1155-1160.**

**22. Hall R, Duarte-Gardea M, Harlass F: Oral versus vaginal misoprostol for labor induction. *Obstet Gynecol* 2002, 99(6):1044-1048.**

**23. Adair CD, Weeks JW, Barrilleaux PS, Philibert L, Edwards MS, Lewis DF: Labor induction with oral versus vaginal misoprostol: a randomized, double-blind trial. *American Journal of Obstetrics and Gynecology* 1998, 178:S93.**

**24. Adair CD, Weeks JW, Barrilleaux S, Edwards M, Burlison K, Lewis DF: Oral or vaginal misoprostol administration for induction of labor: a randomized, double-blind trial. *Obstet Gynecol* 1998, 92(5):810-813.**

**25. Toppozada MK, Anwar MY, Hassan HA, el-Gazaerly WS: Oral or vaginal misoprostol for induction of labor. *Int J Gynaecol Obstet* 1997, 56(2):135-139.**
