# Supplementary material for: Reducing stillbirths: interventions during labour
Source: BMC Pregnancy Childbirth. 2009 May 7;9(Suppl 1):S6. doi: 10.1186/1471-2393-9-S1-S6 (PMC2679412; doi:10.1186/1471-2393-9-S1-S6)
Supplement: Additional file 9 — Web Table 9. Component studies in Hofmeyr and Gulmezoglu. 2003 meta-analysis: Impact of vaginal misoprostol for cervical ripening and labour induction on perinatal mortality. Component studies in Hofmeyr and Gulmezoglu 2003 meta-analysis showing impact on stillbirths/perinatal mortality. [file 1471-2393-9-S1-S6-S9.doc]

**Web Table 9. Component studies in Hofmeyr and Gulmezoglu 2003 meta-analysis [1]: Impact of vaginal misoprostol for cervical ripening and labour induction on perinatal mortality**

| **Source** | **Location and Type of Study** | **Intervention** | **Stillbirths / Perinatal Outcomes** |
| --- | --- | --- | --- |
| **Misoprostol versus oxytocin** | | | |
| 1. Campos Perez 1994 [2-4] | Chile.  RCT. N=153 women (N=78 intervention group, N=75 controls). | Compared the impact of misoprostol 50 microgram tablet vaginally (intervention) vs. intravenous oxytocin 2-32 mU/minute (controls). | PMR: RR not estimable.  [0/77 vs. 0/75 in intervention and control groups, respectively]. |
| **Misoprostol lower vs. higher dose** | | | |
| 2, Majoko 2002a. [5] | Zimbabwe. Harare Maternity Hospital.  RCT. N= 127 pregnant women between June to September 1998. | Compared the impact of half (intervention) vs. quarter (controls) 200 µg misoprostol tablet inserted into posterior vaginal fornix. Repeated after 8 hours if cervical score < 10 (maximum 2 doses). | PMR: RR=0.11 (95% CI: 0.01-1.99) **[NS]**.  [0/64 vs. 4/63 in intervention and control groups, respectively]. |
| **Vaginal misoprostol versus placebo/no treatment** | | | |
| 3. Fletcher 1993 [6] | Jamaica (Kingston).  Double blind RCT. N=45 women. | Compared the impact of powdered misoprostol 100 micrograms (intervention) vs. ethinyl estradiol 0.05 mg ('placebo') (controls), each mixed with hydroxyethyl gel 2.7 mg, administered with a syringe into the posterior vaginal fornix; if not in labour after 12 hours, oxytocin induction was commenced or planned. | PMR: RR not estimable.  [0/24 vs. 0/21 in intervention and control groups, respectively]. |
| **Misoprostol versus vaginal prostaglandin** | | | |
| 4. Kolderup 1999 [7] | USA (California). 3 hospitals.  RCT. N=159 women between January 1994 to December 1996. | Compared the impact of misoprostol 50 micrograms vaginally, repeated 4-hourly if necessary to maximum of 6 doses (intervention) vs. prostaglandin E2 gel 0.5 mg intracervically, repeated 6-hourly if necessary, to a maximum of 4 doses (controls).  Fetal and uterine monitoring for at least 1 hour after treatment. Amniotomy attempted when cervix 3-4 cm dilated. If not in adequate labour 4 hours after last dose or arrest of cervical dilation for more than 2 hours after 4 cm dilation, oxytocin infusion commenced or the woman was crossed over to the other group. | PMR: RR=2.85 (95% CI: 0.12-68.95) **[NS]**.  [1/80 vs. 0/76 in intervention and control groups, respectively]. |
| 5. Fletcher 1994 [8] | Jamaica (Kingston).  RCT. N=63 women between September to October 1992. | Compared the impact of insertion into the posterior vaginal fornix of misoprostol 100 micrograms (intervention) vs. prostaglandin E2 3 mg (controls). If not in labour after 12 hours, oxytocin was commenced (sometimes delayed because of staff shortage). | PMR: RR not estimable.  [0/31in both the groups]. |
| 6. Lee 1997 [9] | Malaysia. Kuantan General Hospital.  Double-blind RCT. N=50 pregnant women. | Compared the impact of misoprostol 200 microgram tablet (intervention) vs. prostaglandin E2 3 mg (controls), inserted vaginally 6-hourly, maximum 2 doses. The cervix was assessed every 6 hours. When 'ready for labour', transferred to the labour ward. If no labour ensued, oxytocin given. If the cervix remained unfavourable after 24 hours, Caesarean section was performed. | PMR: RR not estimable.  [0/25 in both the groups]. |
| **Vaginal misoprostol versus intracervical prostaglandin** | | | |
| 7, Kolderup 1999 [7] | USA (California). 3 hospitals.  RCT. N=159 women between January 1994 to December 1996. | Compared the impact of misoprostol 50 micrograms vaginally, repeated 4-hourly if necessary to maximum of 6 doses (intervention) vs. prostaglandin E2 gel 0.5 mg intracervically, repeated 6-hourly if necessary, to a maximum of 4 doses (controls).  Fetal and uterine monitoring for at least 1 hour after treatment. Amniotomy attempted when cervix 3-4 cm dilated. If not in adequate labour 4 hours after last dose or arrest of cervical dilation for more than 2 hours after 4 cm dilation, oxytocin infusion commenced or the woman was crossed over to the other group. | PMR: RR=2.85 (95% CI: 0.12-68.95) **[NS]**.  [1/80 vs. 0/76 in intervention and control groups, respectively]. |
| 8. Mundle 1996 [10] | Canada (St John’s, Newfoundland).  RCT. N=222 women between March to September 1994. | Compared the impact of misoprostol 50 micrograms in the upper vagina 4 hourly until progressive labour, contraction frequency of 3 per 10 minutes, membranes ruptured, non-reassuring fetal heart rate tracing, delivery or maximum of 16 doses (intervention); vs. physician-chosen combinations of prostaglandin E2 0.5 mg intracervically for cervical ripening or 1-2 mg vaginal gel for induction, and oxytocin infusion (controls). Artificial rupture of membranes in both groups was at the discretion of the attending physician. After membrane rupture, augmentation of labour was by oxytocin infusion. Oxytocin was not allowed within 4 hours of the last misoprostol or 6 hours of the last prostaglandin E2 dose. | PMR: RR not estimable.  [0/111 in both the groups]. |

**References**

**1. Hofmeyr GJ, Gulmezoglu AM: Vaginal misoprostol for cervical ripening and induction of labour. *Cochrane Database Syst Rev* 2003(1):CD000941.**

**2. Campos GA, Guzman S, Rodriguez JG, Voto LS, Margulies M: [Misoprostol--a PGE1 analog for induction of labor at term: comparative and randomized study with oxytocin]. *Rev Chil Obstet Ginecol* 1994, 59(3):190-195; discussion 195-196.**

**3. Campos Perez GA, Marguiles M, Ortega I, Voto LS: Induction of labour with misoprostol, a PGE1 analog. A comparative study. In: *Proceedings of 2nd European Congress on Prostaglandins in Reproduction: 1991; The Hague, Netherlands.*; 1991.**

**4. Margulies M, Campos Perez G, Voto LS: Misoprostol to induce labour. *Lancet* 1992, 339(8784):64.**

**5. Majoko F, Nystrom L, Lindmark G: No benefit, but increased harm from high dose (100 microg) misoprostol for induction of labour: a randomised trial of high vs. low (50 microg) dose misoprostol. *J Obstet Gynaecol* 2002, 22(6):614-617.**

**6. Fletcher HM, Mitchell S, Simeon D, Frederick J, Brown D: Intravaginal misoprostol as a cervical ripening agent. *Br J Obstet Gynaecol* 1993, 100(7):641-644.**

**7. Kolderup L, McLean L, Grullon K, Safford K, Kilpatrick SJ: Misoprostol is more efficacious for labor induction than prostaglandin E2, but is it associated with more risk? *Am J Obstet Gynecol* 1999, 180(6 Pt 1):1543-1550.**

**8. Fletcher H, Mitchell S, Frederick J, Simeon D, Brown D: Intravaginal misoprostol versus dinoprostone as cervical ripening and labor-inducing agents. *Obstet Gynecol* 1994, 83(2):244-247.**

**9. Lee HY: A randomised double-blind study of vaginal misoprostol vs dinoprostone for cervical ripening and labour induction in prolonged pregnancy. *Singapore Med J* 1997, 38(7):292-294.**

**10. Mundle WR, Young DC: Vaginal misoprostol for induction of labor: a randomized controlled trial. *Obstet Gynecol* 1996, 88(4 Pt 1):521-525.**
