# Supplementary material for: Reducing stillbirths: interventions during labour
Source: BMC Pregnancy Childbirth. 2009 May 7;9(Suppl 1):S6. doi: 10.1186/1471-2393-9-S1-S6 (PMC2679412; doi:10.1186/1471-2393-9-S1-S6)
Supplement: Additional file 10 — Web Table 10. Component studies in French 2001: Impact of oral prostaglandin E2 for inducing labour on perinatal mortality. Component studies in French 2001 showing impact on stillbirths/perinatal mortality. [file 1471-2393-9-S1-S6-S10.doc]

**Web Table 10. Component studies in French 2001 [1]: Impact of oral prostaglandin E2 for inducing labour on perinatal mortality**

| **Source** | **Location and Type of Study** | **Intervention** | **Stillbirths / Perinatal Outcomes** |
| --- | --- | --- | --- |
| **Oral prostaglandin vs. interavenous oxytocin** | | | |
| 1. Paul 1992 [2] | India (Calcutta). University Hospital.  RCT. N=35 pregnant women (N=15 intervention group, N=20 controls). | Compared the impact of oral prostaglandin E2 0.5 mg hourly increasing to 1 mg hourly if needed (intervention) vs IV oxytocin to a maximum of 20 miliunits per minute (controls). | PMR: RR not estimable.  [0/15 vs. 0/20 in intervention and control groups, respectively]. |
| 2. Westergaard 1983a [3] | Denmark (Odense). University hospital.  RCT. N=193 women with premature rupture of the membranes after the 37^th^ week of gestation (N=109 intervention group, N=84 controls). | Compared the impact of oral prostaglandin E2 0.5 mg increasing to 1.5 mg per hour if needed (intervention) vs. buccal demoxytocin 50 IU every 1/2 hour (controls). | PMR: RR not estimable.  [0/109 vs. 0/84 in intervention and control groups, respectively]. |

**References**

**1. French L: Oral prostaglandin E2 for induction of labour. *Cochrane Database Syst Rev* 2001(2):CD003098.**

**2. Paul S, Bhowmick R: A randomised controlled trial of oral prostaglandin E2 (dinoprostone) and oxytocin infusion in induction of labour. Personal communication. In*.*; 1992.**

**3. Westergaard JG, Lange AP, Pedersen GT, Secher NJ: Use of oral oxytocics for stimulation of labor in cases of premature rupture of the membranes at term. A randomized comparative study of prostaglandin E2 tablets and demoxytocin resoriblets. *Acta Obstet Gynecol Scand* 1983, 62(2):111-116.**
