# Supplementary material for: Reducing stillbirths: interventions during labour
Source: BMC Pregnancy Childbirth. 2009 May 7;9(Suppl 1):S6. doi: 10.1186/1471-2393-9-S1-S6 (PMC2679412; doi:10.1186/1471-2393-9-S1-S6)
Supplement: Additional file 11 — Web Table 11. Component studies in Boulvain et al. 2008 meta-analysis: Impact of intracervical prostaglandin for labour induction on perinatal mortality. Component studies in Boulvain et al. 2008 meta-analysis showing impact on stillbirths/perinatal mortality. [file 1471-2393-9-S1-S6-S11.doc]

**Web Table 11. Component studies in Boulvain et al. 2008 [1] meta-analysis: Impact of intracervical prostaglandin for labour induction on perinatal mortality**

| **Source** | **Location and Type of Study** | **Intervention** | **Stillbirths / Perinatal Outcomes** |
| --- | --- | --- | --- |
| **Intracervical prostaglandin (prostaglandin E2) vs. placebo/no treatment** | | | |
| 1. NICHHD 1994 [2] | USA.  Double-blind RCT. N=265 women (N=174 intervention group, N=91 controls). | Compared the impact on perinatal mortality of IC gel prostaglandin E2 0.5 mg (1 dose) followed 12 hours later with oxytocin (intervention) vs. IC placebo gel (1 dose) followed 12 hours later with oxytocin (controls).  Subjects underwent induction within 24 hours after randomisation. When clinically feasible, amniotomy was performed before the administration of oxytocin. If amniotomy was not feasible, oxytocin infusion was initiated according to uniform protocol. If the participant had not entered the active phase of labour after 24 hours of oxytocin administration, a CS was performed or induction of labour was continued for a longer time. | PMR: RR not estimable.  [0/174 vs. 0/91 in intervention and control groups, respectively]. |
| 1. Noah 1987 [3] | Europe and Africa.  RCT. N=820 women (N=416 intervention group, N=404 controls). | Assessed the effects of intervention with IC gel prostaglandin E2 0.5 mg (1 dose) vs. no treatment (controls).  At the end of 12 hours, all participants not in labour were treated with an intravenous infusion of oxytocin as the 'initial' induction attempt. Oxytocin was started at 1.0 mU/minute and escalated at 30 to 60 minute intervals as required to a maximum dose of 16 mU/minute. Amniotomy was performed after labour had started. If labour had not started within 12 hours, the attempt at labour induction could be judged a failure. | PMR: RR=0.20 (95% CI: 0.01-4.05) **[NS]**.  [0/413 vs. 2/403 in intervention and control groups, respectively]. |
| **Intracervical prostaglandin (prostaglandin E2) vs vaginal prostaglandin (prostaglandin E2).** | | | |
| 1. Seeras 1995 [4] | UK.  RCT. N=68 women (N=37 intervention group, N=31 controls). | Compared the impact of IC gel prostaglandin E2 0.5 mg 6 hourly, max 3 doses (intervention) vs. vaginal gel prostaglandin E2 2 mg 6 hourly, max 3 doses (controls). | PMR: RR not estimable.  [0/37 vs. 0/31 in intervention and control groups, respectively]. |
| 1. Zanini et al 1989 [5] | Italy.  RCT. N=106 women (N=52 intervention group, N=48 controls). | Assessed the effects of IC gel prostaglandin E2 0.5 mg (intervention) vs. vaginal gel prostaglandin E2 3 mg (controls). | Serious neonatal morbidity/PMR: RR not estimable.  [0/52 vs. 0/48 in intervention and control groups, respectively]. |

**References**

1. Boulvain M, Kelly A, Irion O: **Intracervical prostaglandins for induction of labour**. *Cochrane Database Syst Rev* 2008(1):CD006971.

2. **A clinical trial of induction of labor versus expectant management in postterm pregnancy. The National Institute of Child Health and Human Development Network of Maternal-Fetal Medicine Units**. *Am J Obstet Gynecol* 1994, **170**(3):716-723.

3. Noah ML, DeCoster JM, Fraser TJ, Orr JD: **Preinduction cervical softening with endocervical PGE2 gel. A multi-center trial**. *Acta Obstet Gynecol Scand* 1987, **66**(1):3-7.

4. Seeras RC: **Induction of labor utilizing vaginal vs. intracervical prostaglandin E2**. *Int J Gynaecol Obstet* 1995, **48**(2):163-167.

5. Zanini A, Norchi S, Beretta E, Cortinovis I, Fenaroli G, Scian A: **[Cervical ripening and induction of labor in term pregnancy using prostaglandin E2. Controlled clinical study comparing the intracervical and intravaginal routes]**. *Ann Ostet Ginecol Med Perinat* 1989, **110**(5):209-216.
