# Supplementary material for: Reducing stillbirths: interventions during labour
Source: BMC Pregnancy Childbirth. 2009 May 7;9(Suppl 1):S6. doi: 10.1186/1471-2393-9-S1-S6 (PMC2679412; doi:10.1186/1471-2393-9-S1-S6)
Supplement: Additional file 12 — Web Table 12. Component studies in Kelly et al. 2003 meta-analysis: Impact of vaginal prostaglandin (prostaglandin E2 and PGF2α) for term labour induction on perinatal mortality. Component studies in Kelly et al. 2003 meta-analysis showing impact on stillbirths/perinatal mortality. [file 1471-2393-9-S1-S6-S12.doc]

**Web Table 12. Component studies in Kelly et al. 2003 [1]meta-analysis: Impact of vaginal prostaglandin (prostaglandin E2 and PGF2α) for term labour induction on perinatal mortality**

| **Source** | **Location and Type of Study** | **Intervention** | **Stillbirths / Perinatal Outcomes** |
| --- | --- | --- | --- |
| **Prostaglandin E2 (once only) vs. placebo/no treatment** | | | |
| 1. Cardozo 1986 [2] | UK (London). Kings College Hospital.  Quasi-RCT. N=402 women at 40 weeks plus 10 days of pregnancy (N=195 intervention group, N=207 controls). | Assessed the effect on perinatal mortality of intervention where labour was induced by 3 mg prostaglandin E2 pessary followed by amniotomy 3 hours later +/- oxytocin where necessary. Induction occurred between 40 weeks +12 days and 40 weeks+ 14 days. The conservative group (controls) had fetal assessment by ultrasound, daily kick charts and alternate date CTGs. | PMR: RR=1.06 (95% CI: 0.07-16.85) **[NS]**.  [1/195 vs. 1/207 in intervention and control groups, respectively]. |
| 2. Prins 1983 [3] | USA. Oregon Health Sciences University.  RCT. N=30 patients requiring induction labour (N=15 intervention group, N=15 controls). | Compared the impact on perinatal mortality of 2.5 mg prostaglandin E2 vaginal gel (intervention) vs. identical placebo (controls).  Bishop score assigned at instillation and the following morning prior to commencement of oxytocin. | PMR: RR not estimable.  [0/15 vs. 0/14 in intervention and control groups, respectively]. |
| **Prostaglandin E2 (repeated doses) vs. placebo/no treatment** | | | |
| 3. Egarter 1989 [4] | Austria (Vienna).  RCT. N=345 women undergoing induction of labour (N=180 intervention group). | Assessed the effect on perinatal mortality of 3 mg prostaglandin E2 vaginal tablet (intervention) with a repeat at 6 hours. If not given birth at 24 hours and cervix > 3 cm dilated, a repeat course was given. If < 3 cm dilated no further induction was undertaken. In control group, spontaneous labour was awaited until 42 weeks amenorrhea. | PMR: RR=0.31 (95% CI: 0.01-7.45) [NS].  [0/180 vs. 1/165 in intervention and control groups, respectively]. |
| 4. Hannah 1996; Gafni A, et al. 1997 [5, 6] | Canada.  RCT. N=5041 women with prelabour rupture of the membranes at term. | Compared the impact of IV oxytocin, immediate (intervention # 1) vs. vaginal prostaglandin E2 q6h x 2, then IV oxytocin if still not in labour (intervention # 2) vs. expectant management x 96 hrs, IV oxytocin if still not in labour (control # 1) vs. expectant management x 96 hrs, vaginal prostaglandin E2 as above if still not in labour (control # 2). | PMR: RR=0.20 (95% CI: 0.01-4.17) **[NS]**.  [0/1258 vs. 2/1261 in intervention vs. control groups, respectively]. |
| 5. Liggins 1979 [7] | New Zealand. University of Auckland.  RCT. N=84 women requiring induction of labour for major or minor complications of pregnancy (N=26 intervention group # 1, N=26 intervention # 2, N=32 controls). | Compared the impact of 0.2 mg (intervention # 1) vs. 0.4 mg (intervention # 2) prostaglandin E2 vaginal suppositories vs. identical placebo (control) placed at 0900; then self administered repeat suppositories at 2 hourly intervals. Rested overnight and continued until 15 suppositories used or labour ensued.  If not in labour after 48 hours patients underwent induction by amniotomy and oxytocin. | PMR: RR=1.87 (95% CI: 0.08-44.51) **[NS]**.  [1/52 vs. 0/32 in intervention and control groups, respectively]. |
| 6. Shoaib 1994 [8] | Pakistan (Lahore). Allama Iqbal Medical School.  RCT. N=200 primigravid women (N=100 intervention group, N=100 controls). | Compared the impact of active management with 3 mg prostaglandin E2 vaginal tablets (intervention) vs. conservative management.  prostaglandin E2 tablets every 6 hours to a maximum of 3. No details given of conservative management. | PMR: RR not estimable.  [0/100 in both the groups]. |
| **Prostaglandin E2 (sustained release) vs. placebo/no treatment** | | | |
| 7. Prasad 1989 [9] | Singapore. National University Hospital.  RCT. N=69 women requiring induction of labour (N=33 intervention group, N=36 controls). | Compared the impact on perinatal mortality of prostaglandin E2 vaginal film (8.5 mg in 24 hours) (intervention) vs. identical placebo (controls).  Baseline Bishop score and repeat at 12 and 24 hours. | PMR: RR not estimable.  [0/33 vs. 0/36 in intervention and control groups, respectively]. |
| **Prostaglandin E2 low dose vs. prostaglandin E2 high dose** | | | |
| 8. Mac Kenzie 1997 [10] | UK. John Radcliffe Hospital.  RCT. N=955 women requiring induction of labour (N=483 low dose group, N=472 high dose). | Compared the impact on perinatal mortality of 2 mg prostaglandin E2 vaginal gel once only (comparison # 1) vs. repeated dose (comparison # 2). | PMR: RR not estimable.  [0/483 vs. 0/472 in comparison groups # 1 and 2, respectively]. |

**References**

**1. Kelly AJ, Kavanagh J, Thomas J: Vaginal prostaglandin (PGE2 and PGF2a) for induction of labour at term. *Cochrane Database Syst Rev* 2003(4):CD003101.**

**2. Cardozo L, Fysh J, Pearce JM: Prolonged pregnancy: the management debate. *Br Med J (Clin Res Ed)* 1986, 293(6554):1059-1063.**

**3. Prins RP, Bolton RN, Mark C, 3rd, Neilson DR, Watson P: Cervical ripening with intravaginal prostaglandin E2 gel. *Obstet Gynecol* 1983, 61(4):459-462.**

**4. Egarter C, Kofler E, Fitz R, Husslein P: Is induction of labor indicated in prolonged pregnancy? Results of a prospective randomised trial. *Gynecol Obstet Invest* 1989, 27(1):6-9.**

**5. Gafni A, Goeree R, Myhr TL, Hannah ME, Blackhouse G, Willan AR, Weston JA, Wang EE, Hodnett ED, Hewson SA *et al*: Induction of labour versus expectant management for prelabour rupture of the membranes at term: an economic evaluation. TERMPROM Study Group. Term Prelabour Rupture of the Membranes. *CMAJ* 1997, 157(11):1519-1525.**

**6. Hannah ME, Ohlsson A, Farine D, Hewson SA, Hodnett ED, Myhr TL, Wang EE, Weston JA, Willan AR: Induction of labor compared with expectant management for prelabor rupture of the membranes at term. TERMPROM Study Group. *N Engl J Med* 1996, 334(16):1005-1010.**

**7. Liggins GC: Controlled trial of induction of labor by vaginal suppositories containing prostaglandin E2. *Prostaglandins* 1979, 18(1):167-172.**

**8. Shoaib F: Management of premature rupture of memebranes with unfavourbale cervix at term, by prostaglandins. *Pakistan's Journal of Medical Science;* 1994, 10:227-232.**

**9. Prasad RN, Adaikan PG, Arulkumaran S, Ratnam SS: Preinduction cervical priming with PGE2 vaginal film in primigravidae--a randomised, double blind, placebo controlled study. *Prostaglandins Leukot Essent Fatty Acids* 1989, 36(3):185-188.**

**10. MacKenzie IZ, Burns E: Randomised trial of one versus two doses of prostaglandin E2 for induction of labour: 1. Clinical outcome. *Br J Obstet Gynaecol* 1997, 104(9):1062-1067.**
