# Supplementary material for: Reducing stillbirths: interventions during labour
Source: BMC Pregnancy Childbirth. 2009 May 7;9(Suppl 1):S6. doi: 10.1186/1471-2393-9-S1-S6 (PMC2679412; doi:10.1186/1471-2393-9-S1-S6)
Supplement: Additional file 13 — Web Table 13. Component studies in Hutton and Mozurkewich 2001: Impact of extra-amniotic prostaglandin for labour induction on perinatal mortality. Component studies in Hutton and Mozurkewich 2001 meta-analysis showing impact on stillbirths/perinatal mortality. [file 1471-2393-9-S1-S6-S13.doc]

**Web Table 13. Component studies in Hutton and Mozurkewich 2001 [1]: Impact of extra-amniotic prostaglandin for labour induction on perinatal mortality**

| **Source** | **Location and Type of Study** | **Intervention** | **Stillbirths / Perinatal Outcomes** |
| --- | --- | --- | --- |
| **Extra amniotic PGF2 alpha vs. Foley catheter** | | | |
| 1. Mahomed 1988 [2] | Zimbabwe.  RCT. N=77 singleton, live vertex, Bishop's score < 6, admitted for induction. | Compared the impact of 2.5 mg PGF2alpha in 20 ml Tylose gel via Foley catheter (intervention) vs. Foley catheter with traction (controls). | PMR: RR not estimable.  [0/38 vs. 0/39 in intervention and control groups, respectively]. |
| **Extra amniotic PGF2 alpha vs. extra amniotic placebo gel** | | | |
| 1. Quinn 1981 [3] | Australia.  Double blind trial. N=40 nulliparous, modified Bishop's score < 4. | Extra-amniotic: - placebo gel - 10 mg PGF2alpha in Tylose gel - 15 mg oestriol in tylose gel. | PMR: RR=2.06 (95% CI: 0.09-46.11) **[NS]**.  [1/15 vs. 0/10 in PGF2alpha and control groups, respectively]. |

**References**

**1. Hutton E, Mozurkewich E: Extra-amniotic prostaglandin for induction of labour. *Cochrane Database Syst Rev* 2001(2):CD003092.**

**2. Mahomed K: Foley catheter under traction versus extra-amniotic prostaglandin gel in pre-treatment of unripe cervix--a randomised controlled trial. *Cent Afr J Med* 1988, 34(5):98-102.**

**3. Quinn MA, Murphy AJ, Kuhn RJ, Robinson HP, Brown JB: A double blind trial of extra-amniotic oestriol and prostaglandin F2 alpha gels in cervical ripening. *Br J Obstet Gynaecol* 1981, 88(6):644-649.**
