# Supplementary material for: Reducing stillbirths: interventions during labour
Source: BMC Pregnancy Childbirth. 2009 May 7;9(Suppl 1):S6. doi: 10.1186/1471-2393-9-S1-S6 (PMC2679412; doi:10.1186/1471-2393-9-S1-S6)
Supplement: Additional file 14 — Web Table 14. Component studies in Luckas et al. 2000 meta-analysis: Impact of intravenous prostaglandin for induction of labour on perinatal mortality. Component studies in Luckas et al. 2000 meta-analysis showing impact on stillbirths/perinatal mortality. [file 1471-2393-9-S1-S6-S14.doc]

**Web Table 14. Component studies in Luckas et al. 2000 [1] meta-analysis: Impact of intravenous prostaglandin for induction of labour on perinatal mortality**

| **Source** | **Location and Type of Study** | **Intervention** | **Stillbirths / Perinatal Outcomes** |
| --- | --- | --- | --- |
| **Intravenous (IV) prostaglandins vs. IV oxytocin** | | | |
| 1. Baxi 1980 [2] | USA.  RCT. N=50 women between 37 and 43 weeks. | Compared the impact of intravenous prostaglandin F2alpha (maximum 20 ug/min) (intervention) vs. intravenous oxytocin (maximum 16 mU/min) (controls). Infusions doubled every hour until labour was established (maximum of three doublings). If after 10 hours, labour did not ensue, other unspecified methods were used. Amniotomy was performed after 90 minutes or when possible. | PMR: RR=3.00 (95% CI: 0.13-70.30) **[NS]**.  [1/25 vs. 0/25 in intervention and control groups, respectively]. |
| 2. Beazley 1970 [3] | UK (London). Queen Charlotte’s Maternity Hospital.  RCT. N=300 women, 35 to 43 weeks. | Compared the impact of intravenous prostaglandin E2 (maximum 6.7 ug/min) (intervention) vs. intravenous oxytocin (maximum 67 mU/min) (controls). Infusions doubled every hour until labour was established. Successful induction was deemed as a cervical dilatation of 6 cm or delivery was achieved within 12 hours. Amniotomy performed at an unspecified time. | PMR: RR=5.00 (95% CI: 0.24-103.28) [NS].  [2/150 vs. 0/150 in intervention and control groups, respectively]. |
| 3. Calder 1975 [4] | UK (Oxford).  RCT; N=100 primigravidas with bishop score 6 or less at 37-42 weeks. | Assessed the effects of prostaglandin E2 intravenously doubling hourly to a maximum of 4 ug/min vs. intravenous oxytocin doubling hourly to a maximum of 64 mu/min. All participants had amniotomy at the start. Half of each group received the infusion via an automatic infusor correlated to intrauterine pressures. All women had epidurals. Amniotomy was performed when possible (not specified). | PMR: RR not estimable.  [0/50 in both groups]. |
| 4. Moller 1987 [5] | Denmark.  RCT. N=100 women with pre-labour rupture of the membranes at 37-42 weeks gestation. | Compared the impact of intravenous prostaglandin F2 alpha (maximum of 6 ug/min) (intervention) vs. intravenous oxytocin (maximum 30 mU/min) (controls). | PMR: RR not estimable.  [0/50 in both groups]. |
| 5. Naismith 1973 [6] | UK.  RCT. N=40 women with singleton gestations and intact membranes all of whom were post term (N=10 PGF2 alpha, N=10 prostaglandin E2, N=20 controls). | Compared the impact of prostaglandin F2 alpha (intervention # 1) increasing to a maximum of 80 ug/min vs. prostaglandin E2 (intervention # 2) to a maximum of 8 ug/min vs. oxytocin (controls) increasing to a maximum of 340 mU/minute. Amniotomy being performed when labour was established. | PMR: RR not estimable.  [0/20 vs. 0/20 in the intervention and control groups, respectively]. |
| 6. Spellacy 1973 [7] | USA.  RCT. N=222 women between 36 and 43 weeks (N=115 intervention group, N=107 controls). | Compared the impact of prostaglandin F 2 alpha increasing to a maximum of 40 ug/min (intervention) vs. oxytocin increasing to a maximum of 8 mU/min (controls). Amniotomy was performed 90 minutes after labour was judged to have commenced. | PMR: RR=2.79 (95% CI: 0.12-67.83) [NS].  [1/115 vs. 0/107 in intervention and control groups, respectively]. |
| 7. Vakhariya 1972 [8] | USA.  RCT. N=100 multiparous women at 36-43 weeks of gestation (N=50 intervention group, N=50 controls). | Compared the impact of intravenous prostaglandin F2 alpha increasing to a maximum of 40 ug/minute (intervention) vs. oxytocin increasing to a maximum of 16 mU/min (controls). Amniotomy was performed when possible (not specified). | PMR: RR not estimable.  [0/50 in both groups]. |
| 8. Vroman 1972 [9] | Belgium.  RCT. N=50 multigravid women at 38-42 weeks of gestation. | Compared the impact on perinatal mortality of intravenous prostaglandin F2alpha to a maximum of 40 ug/min (intervention) vs. oxytocin to a maximum of 16 mU/minute (controls).  Amniotomy was performed 90 minutes after the onset of labour. | PMR: RR not estimable.  [0/25 in both groups]. |
| 9. Wildemeersch 1976 [10] | The Netherlands.  RCT. N=28 women at 37-42 weeks with no complications (N=14 intervention, N=14 controls). | Compared the impact of prostaglandin F2 alpha increasing to 20 ug/minute (intervention) vs. oxytocin increasing to 8 mU/minute (controls). Amniotomy when possible. | PMR: RR not estimable.  [0/14 in both groups]. |
| **IV prostaglandin E2 and oxytocin vs. IV oxytocin** | | | |
| 10. Naismith 1972 [11] | UK (Glasgow).  RCT. N=20 primigravid women who were post term with singleton cephalically presenting fetuses. | Compared the impact of oxytocin (maximum of 300 mU/min) and intravenous prostaglandin E2 (0.5 ug/min fixed rate) (intervention) vs. oxytocin (maximum of 300 mU/min) (controls). Amniotomy was performed at the start in each case. | Serious neonatal morbidity or PMR: RR not estimable.  [0/10 in both groups]. |
| **IV prostaglandin E2 vs. extra-amniotic prostaglandin E2** | | | |
| 11. Iskander 1978 [12] | England.  RCT. N=40 women with Bishop score of 6 or less. | Compared the impact of intravenous prostaglandin E2 up to a maximum of 1 ug/minute (intervention) vs. extra amniotic infusion (controls) up to a maximum of 150 ug/hour. Amniotomy was performed when possible. | PMR: RR not estimable.  [0/20 in both groups]. |

**References**

**1. Luckas M, Bricker L: Intravenous prostaglandin for induction of labour. *Cochrane Database Syst Rev* 2000(4):CD002864.**

**2. Baxi LV, Petrie RH, Caritis SN: Induction of labor with low-dose prostaglandin F2 alpha and oxytocin. *Am J Obstet Gynecol* 1980, 136(1):28-31.**

**3. Beazley JM, Gillespie A: Double-bli trial of prostaglandin E2 and oxytocin in induction of labour. *Lancet* 1971, 1(7691):152-155.**

**4. Calder AA, Embrey MP: Comparison of intravenous oxytocin and prostaglandin E2 for induction of labour using automatic and non-automatic infusion techniques. *Br J Obstet Gynaecol* 1975, 82(9):728-733.**

**5. Moller M, Thomsen AC, Sorensen J, Forman A: Oxytocin- or low-dose prostaglandin F2 alpha-infusion for stimulation of labor after primary rupture of membranes. A prospective, randomized trial. *Acta Obstet Gynecol Scand* 1987, 66(2):103-106.**

**6. Naismith WC, Barr W, MacVicar J: Comparison of intravenous prostaglandins F 2 and E 2 with intravenous oxytocin in the induction of labour. *J Obstet Gynaecol Br Commonw* 1973, 80(6):531-535.**

**7. Spellacy WN, Gall SA, Shevach AB, Holsinger KK: The induction of labor at term. Comparisons between prostaglandin F2alpha and oxytocin infusion. *Obstetrics & Gynecology;* 1973, 41:14-21.**

**8. Vakhariya VR, Sherman AI: Prostaglandin F 2 for induction of labor. *Am J Obstet Gynecol* 1972, 113(2):212-222.**

**9. Vroman S, Thiery M, Yo Le Sian A, Depiere M, Vanderheyden C, Derom R, al e: A double blind comparative study of prostaglandin F2alpha and oxytocin for the elective induction of labor. *European Journal of Obstetrics & Gynecology and Reproductive Biology;* 1972, 4S:115-123.**

**10. Wildemeersch DA, Schellen AM: Double-blind trial of prostaglandin F2alpha and oxytocin in the induction of labour. *Curr Med Res Opin* 1976, 4(4):263-266.**

**11. Naismith WC, Barr W, MacVicar J: Induction of labour by simultaneous intravenous administration of prostaglandin E 2 and oxytocin. *Br Med J* 1972, 4(5838):461-462.**

**12. Iskander MN: A comparison of the efficacy and safety of extra-amniotic prostaglandin E2 and intravenous prostaglandin E2 for the induction of labour in patients with unripe cervices. *J Int Med Res* 1978, 6(2):144-146.**
