# Supplementary material for: Reducing stillbirths: interventions during labour
Source: BMC Pregnancy Childbirth. 2009 May 7;9(Suppl 1):S6. doi: 10.1186/1471-2393-9-S1-S6 (PMC2679412; doi:10.1186/1471-2393-9-S1-S6)
Supplement: Additional file 15 — Web Table 15. Component studies in Neilson 2000 meta-analysis: Impact of mifepristone for labour induction on perinatal mortality. Component studies in Neilson 2000 meta-analysis showing impact on stillbirths/perinatal mortality. [file 1471-2393-9-S1-S6-S15.doc]

**Web Table 15. Component studies in Neilson 2000 meta-analysis [1]: Impact of mifepristone for labour induction on perinatal mortality**

| **Source** | **Location and Type of Study** | **Intervention** | **Stillbirths / Perinatal Outcomes** |
| --- | --- | --- | --- |
| 1. Lelaidier 1994 [2] | France.  RCT. N=32 women with one previous child delivered by lower segment Caesarean section, and indication for planned delivery and Bishop score < 3. France. | Compared the impact of mifepristone 200mg (intervention) vs. placebo (controls), given on days 1 and 2. Labour induced (by prostaglandins or ARM + oxytocin) day 4 if woman not in labour by then. | PMR: RR not estimable.  [0/16 in both the groups]. |
| 2. Stenlund 1999 [3] | Sweden.  RCT. N=36 women with indication for planned delivery. | Assessed the effect of 400mg mifepristone (intervention) vs. placebo (controls). If labour had not started within 48 hours, labour was induced with intracervical prostaglandins. | PMR: RR not estimable.  [0/24 vs. 0/12 in intervention and control groups, respectively]. |

**References**

**1. Neilson JP: Mifepristone for induction of labour. *Cochrane Database Syst Rev* 2000(4):CD002865.**

**2. Lelaidier C, Baton C, Benifla JL, Fernandez H, Bourget P, Frydman R: Mifepristone for labour induction after previous caesarean section. *Br J Obstet Gynaecol* 1994, 101(6):501-503.**

**3. Stenlund PM, Ekman G, Aedo AR, Bygdeman M: Induction of labor with mifepristone--a randomized, double-blind study versus placebo. *Acta Obstet Gynecol Scand* 1999, 78(9):793-798.**
