# Supplementary material for: Reducing stillbirths: interventions during labour
Source: BMC Pregnancy Childbirth. 2009 May 7;9(Suppl 1):S6. doi: 10.1186/1471-2393-9-S1-S6 (PMC2679412; doi:10.1186/1471-2393-9-S1-S6)
Supplement: Additional file 16 — Web Table 16. Component studies in Hofmeyr and Hannah 2003 meta-analysis: Impact of planned Caesarean section for term breech on perinatal/neonatal mortality. Component studies in Hofmeyr and Hannah 2003 meta-analysis showing impact on stillbirths/perinatal mortality. [file 1471-2393-9-S1-S6-S16.doc]

**Web Table 16. Component studies in Hofmeyr and Hannah 2003 [1] meta-analysis: Impact of planned Caesarean section for term breech on perinatal/neonatal mortality**

| **Source** | **Location and Type of Study** | **Intervention** | **Stillbirth / Perinatal Outcomes** |
| --- | --- | --- | --- |
| **Low national perinatal mortality rate** | | | |
| 1. Collea et al. 1980 [2, 3] | USA (Los Angeles, California).  RCT. N=208 women (N=93 intervention group, N=115 controls). | Compared the impact of elective Caesarean section (intervention) vs. vaginal delivery (controls). | SBR: OR=0.25 (95% CI: 0.03-2.08) **[NS]**.  [1/93 vs. 5/115 in intervention and control groups, respectively].  PMR/NMR (excluding fatal malformations): RR not estimable.  [0/93 vs. 0/114 in intervention and control groups, respectively]. |
| 2. Gimovsky et al. 1983 [4] | USA (Los Angeles. California).  RCT. N=105 women (N=35 intervention group, N=70 controls). | Compared the impact of planned elective Caesarean section (intervention) vs. planned trial of labour (controls). | PMR/NMR (excluding fatal malformations): RR=0.67 (95% CI: 0.03-15.95) **[NS]**.  [0/34 vs. 1/69 in intervention and control groups, respectively]. |
| 3. Hannah et al. 2000 [5] [6] [7] [8] [9] [10] [11] [12] [13] [14] [15] [16] [17] [18] [19] [20] [21] [22] [23] [24] [25] | Multicentred.  RCT. N= 2083 women. | Compared the impact of planned Caesarean section (intervention) vs. planned vaginal birth (controls). | PMR/NMR (overall): OR=0.23 (95% CI: 0.07-0.81); P=0.01.  [3/1039 (0.3%) vs. 13/1039 (1.3%) in intervention and control groups, respectively].  PMR/NMR (excluding fatal malformations) in subjects from countries with a low perinatal mortality rate: RR=0.14 (95% CI: 0.01-2.74) **[NS]**.  [0/514 vs. 3/511 in intervention and control groups, respectively]. |
| **High national perinatal mortality rate** | | | |
| 3. Hannah et al. 2000 [5-25] | Multicentred.  RCT. N=2083 women. | Compared the impact of planned Caesarean section (intervention) vs. planned vaginal birth (controls). | PMR/NMR (overall): OR=0.23 (95% CI: 0.07-0.81); P=0.01.  [3/1039 (0.3%) vs. 13/1039 (1.3%) in intervention and control groups, respectively].  PMR/NMR (excluding fatal malformations) in subjects from countries with a high perinatal mortality rate: RR=0.30 (95% CI: 0.08-1.09) **[NS]**.  [3/525 vs. 10/528 in intervention and control groups, respectively]. |

**References**

**1. Hofmeyr GJ, Hannah ME: Planned caesarean section for term breech delivery. *Cochrane Database Syst Rev* 2003(3):CD000166.**

**2. Collea JV, Chein C, Quilligan EJ: The randomized management of term frank breech presentation: a study of 208 cases. *Am J Obstet Gynecol* 1980, 137(2):235-244.**

**3. Collea JV, Rabin SC, Weghorst GR, Quilligan EJ: The randomized management of term frank breech presentation: vaginal delivery vs. cesarean section. *Am J Obstet Gynecol* 1978, 131(2):186-195.**

**4. Gimovsky ML, Wallace RL, Schifrin BS, Paul RH: Randomized management of the nonfrank breech presentation at term: a preliminary report. *Am J Obstet Gynecol* 1983, 146(1):34-40.**

**5. Hannah M, Amankwah K, Chalmers B, Cheng M, Foster G, Guselle P, al e: Term Breech Trial (TBT): a RCT of planned caesarean section vs planned vaginal birth for breech at term. In: *12th EAGO Conference: 1997 June 25-28; Dublin, Ireland*; 1997 June 25-28.**

**6. Hannah M, Amankwah K, Chalmers B, Cheng M, Foster G, Guselle P, al e: Term Breech Trial (TBT): a RCT of planned caesarean section vs planned vaginal birth for breech at term. In: *Society of Obstetricians and Gynaecologists of Canada: 1997; Halifax, Nova Scotia, Canada*; 1997.**

**7. Hannah M, Hannah W, for the TBT Group: Term Breech Trial (TBT): a RCT of planned caesarean section vs planned vaginal birth for breech at term. In: *XV FIGO World Congress: 1997 August 3-8; Copenhagen, Denmark*; 1997 August 3-8.**

**8. Hannah ME, Hannah WJ, Hodnett ED, Chalmers B, Kung R, Willan A, Amankwah K, Cheng M, Helewa M, Hewson S *et al*: Outcomes at 3 months after planned cesarean vs planned vaginal delivery for breech presentation at term: the international randomized Term Breech Trial. *JAMA* 2002, 287(14):1822-1831.**

**9. Hannah M, Hannah W, Hodnett E, Chalmers B, Kung R, Willan A, al e: Outcomes at three months postpartum for women enrolled in the multicentre international term breech trial of planned caesarean section and planned vaginal birth for breech presentation at term. *American Journal of Obstetrics and Gynecology;* 2001, 185:S114.**

**10. Hannah M, Hannah W, for the Term Breech Trial Group: Term Breech Trial: a randomized controlled trial of planned CS vs planned vaginal birth for breech at term. In: *XVI FIGO: 2000 September 3-8.; Washington DC.*; 2000 September 3-8.**

**11. Hannah ME, Hannah WJ: Term Breech Trial (TBT): a randomised controlled trial (RCT) of planned Caesarean section vs planned vaginal birth for breech at term. In: *Society of Obstetricians and Gynaecologists of Canada: 2000 June 24-29; Montreal, Canada* 2000 June 24-29.**

**12. Hannah ME, Hannah WJ, Hewson SA, Hodnett ED, Saigal S, Willan AR: Planned caesarean section versus planned vaginal birth for breech presentation at term: a randomised multicentre trial. Term Breech Trial Collaborative Group. *Lancet* 2000, 356(9239):1375-1383.**

**13. Hannah M, Whyte H, Hannah W, Term Breech Trial Collaborative Group: Maternal outcome at 2 years postpartum in the Term Breech Trial. *American Journal of Obstetrics and Gynecology;* 2003, 189:S136.**

**14. Su M, McLeod L, Ross S, Willan A, Hannah WJ, Hutton E, Hewson S, Hannah ME: Factors associated with adverse perinatal outcome in the Term Breech Trial. *Am J Obstet Gynecol* 2003, 189(3):740-745.**

**15. Whyte H, Hannah M, Saigal S, Term Breech Trial Collaborative Group: Outcomes of children at 2 years of age in the Term Breech Trial. *American Journal of Obstetrics and Gynecology;* 2003, 189:S57.**

**16. Hannah ME, Hannah W, Amankwah K, Cheng M, Chalmers B, Foster G: Term Breech Trial (TBT): a RCT of planned caesarean section vs planned vaginal birth for breech at term. In: *Society of Obstetricians and Gynaecologists of Canada: 1999 June; Ottawa, Canada* 1999 June.**

**17. Hannah ME, Moutquin JM, Hannah W, the TBT Group: Term Breech Trial (TBT): a randomised controlled trial (RCT) of planned Caesarean section vs planned vaginal birth for breech at term. In: *First Congress on Obstetrics, Gynecology & Infertility: 1999; Prague, Czech Republic*; 1999.**

**18. Hannah ME, Hannah W, Amankwah K, Cheng M, Chalmers B, Foster G, al e: Term Breech Trial (TBT): a RCT of planned caesarean section vs planned vaginal birth for breech at term. In: *Birth Conference: 1998 June 5-7; Boston, MA.*; 1998 June 5-7.**

**19. Hannah ME, Hannah W, Amankwah K, Cheng M, Chalmers B, Foster G, al e: Term Breech Trial (TBT): a RCT of planned caesarean section vs planned vaginal birth for breech at term. In: *Society of Obstetricians and Gynaecologists of Canada: 1998 June 25-28; Victoria, BC, Canada*; 1998 June 25-28.**

**20. Walkinshaw S, Hannah M, Hannah W: Term breech trial (TBT): a randomised controlled trial of planned caesarean section against planned vaginal birth for breech presentation at term. *British Journal of Obstetrics and Gynaecology;* 1998, 105:65.**

**21. Hannah ME, Whyte H, Hannah WJ, Hewson S, Amankwah K, Cheng M, Gafni A, Guselle P, Helewa M, Hodnett ED *et al*: Maternal outcomes at 2 years after planned cesarean section versus planned vaginal birth for breech presentation at term: the international randomized Term Breech Trial. *Am J Obstet Gynecol* 2004, 191(3):917-927.**

**22. Su M, Hannah WJ, Willan A, Ross S, Hannah ME: Planned caesarean section decreases the risk of adverse perinatal outcome due to both labour and delivery complications in the Term Breech Trial. *BJOG* 2004, 111(10):1065-1074.**

**23. Whyte H, Hannah ME, Saigal S, Hannah WJ, Hewson S, Amankwah K, Cheng M, Gafni A, Guselle P, Helewa M *et al*: Outcomes of children at 2 years after planned cesarean birth versus planned vaginal birth for breech presentation at term: the International Randomized Term Breech Trial. *Am J Obstet Gynecol* 2004, 191(3):864-871.**

**24. McCleod L, Su M, Ross S, Hannah WJ, Willan A, Hutton E, al e: Predictors of maternal mortality or serious maternal morbidity in the term breech trial. *American Journal of Obstetrics and Gynecology;* 2002, 187:S100.**

**25. Su M, McCleod L, Ross S, Willan A, Hannah WJ, Hutton E, al e: Factors associated with adverse perinatal outcome in the term breech trial *American Journal of Obstetrics and Gynecology;* 2002, 187:S69.**
