# Supplementary material for: Reducing stillbirths: interventions during labour
Source: BMC Pregnancy Childbirth. 2009 May 7;9(Suppl 1):S6. doi: 10.1186/1471-2393-9-S1-S6 (PMC2679412; doi:10.1186/1471-2393-9-S1-S6)
Supplement: Additional file 17 — Web Table 17. Component studies in Duley et al. 2003 meta-analysis: Impact of magnesium sulphate and other anti-convulsants for pre-eclampsia on stillbirth and perinatal mortality. Component studies in Duley et al. 2003 meta-analysis showing impact on stillbirths/perinatal mortality. [file 1471-2393-9-S1-S6-S17.doc]

**Web Table 17. Component studies in Duley et al. 2003 [1] meta-analysis: Impact of magnesium sulphate and other anti-convulsants for pre-eclampsia on stillbirth and perinatal mortality**

| **Source** | **Location and Type of Study** | **Intervention** | **Stillbirths / Perinatal Outcomes** |
| --- | --- | --- | --- |
| **Magnesium sulphate versus none/placebo** | | | |
| 1. Magpie Trial 2002 [2] | 33 countries. Multicentred (175 centres).  RCT. N=10141 women with uncertainty about whether to use MgSO4, before birth or 24 hours postpartum, DBP >/= 90 mmHg, SBP >/= 140 mmHg x 2 30-30 min apart, >/= 1+ proteinuria. | Compared the impact of intervention with MgSO4: 4 g IV bolus. Then either 1 g/hr iv infusion or 10 g IM with bolus followed by 5 g every 4 hr. Continued for 24 hr. 2 centres in Bangladesh used 5 g IM then 2.5 g every 4 hr. The control group was given placebo: by identical regimen.  Dose halved if oliguria. Clinical monitoring alone for all women. | SBR: RR=0.96 (95% CI: 0.84 – 1.10) **[NS]**.  [373/4538 vs. 384/4486 in intervention and control groups, respectively].  PMR: RR=0.99 (95% CI: 0.88 – 1.11) **[NS]**.  [518/4538 vs. 516/4486 in intervention and control groups, respectively].  NMR: RR=1.16 (95% CI: 0.94 – 1.42) **[NS]**.  [187/4162 vs. 159/4098 in intervention and control groups, respectively].  IMR: RR=1.05 (95% CI: 0.52 – 2.12) **[NS]**.  [16/4162 vs. 15/4098 in intervention and control groups, respectively]. |
| 2.. Moodley 1994 [3] | South Africa.  RCT. N=228 women with severe PE: DBP >/= 110 mmHg for 4-6 hours, proteinuria +, and delivery imminent. | Compared the impact of intervention with MgSO4: 4 g IV over 20 min and 10 g IM (5 g into each buttock), then 5 g 4 hourly for 24 hours. The control group got no anti-convulsant. | SBR: RR=0.94 (95% CI: 0.46 – 1.91) **[NS]**.  [13/117 vs. 14/118 in intervention and control groups, respectively].  PMR: RR=0.81 (95% CI: 0.48 – 1.37) **[NS]**.  [20/117 vs. 25/118 in intervention and control groups, respectively]. |
| 3. Coetzee et al. 1988 [4] | South Africa.  RCT. N=822 women with severe PE: at least 2 of DBP >/= 110 mmHg, significant proteinuria, symptoms of imminent eclampsia. Also, > 16 years, no previous anti-convulsant (except clonazepam). | Compared the impact of intervention with MgSO4: 4 g IV in 200 ml saline over 20 min, then Ig/hr (200 ml over 4 hr) until 24 hr after delivery. The control group was given placebo: 200 ml over 20 min, then 200 ml over 4 hours until 24 hr after delivery.  Treatment stopped if urine output < 30 ml/hr. Serum monitoring not required. | SBR: RR=1.38 (95% CI: 0.87 – 2.20) **[NS]**.  [38/348 vs. 28/354 in intervention and control groups, respectively]. |
| **Magnesium sulphate versus diazepam** | | | |
| 4. Adeeb et al. 1994 [5, 6] | Malaysia.  RCT. N=28 women with PE (DBP > 110mmHg + proteinuria) and 11 women with eclampsia (data not included). | Compared the impact of MgSO4: 'Pritchard's regimen', no other information (intervention) vs. diazepam: not stated (controls). | SBR: RR=not estimable.  [0/10 vs. 0/18 in intervention and control groups, respectively].  PMR: RR=not estimable.  [0/10 vs. 0/18 in intervention and control groups, respectively]. |
| **Magnesium sulphate versus phenytoin** | | | |
| 5. Lucas et al. 1995 [7] | USA (Texas).  RCT. N=2138 women with BP >/= 140/90 mmHg. Excluded if postpartum or delivery imminent, epilepsy, or eclampsia. | Compared the impact of intervention with MgSO4: 10 g (50% solution) IM (5 g in each buttock), then 5 g IM every 4 hours. If severe pre-eclampsia, an additional 4 g IV (20% solution) before the first IM dose. The control group was given phenytoin: 1000 mg IV over 1 hour. 10 hours later, 500 mg orally.  If eclampsia developed, all women received MgSO4. | SBR: RR=0.62 (95% CI: 0.27 – 1.41) **[NS]**.  [9/1064 vs. 15/1101 in intervention and control groups, respectively].  NMR: RR=0.84 (95% CI: 0.41 – 1.74) **[NS]**.  [13/1064 vs. 16/1101 in intervention and control groups, respectively]. |

**References**

**1. Duley L, Gulmezoglu AM, Henderson-Smart DJ: Magnesium sulphate and other anticonvulsants for women with pre-eclampsia. *Cochrane Database Syst Rev* 2003(2):CD000025.**

**2. Altman D, Carroli G, Duley L, Farrell B, Moodley J, Neilson J, Smith D: Do women with pre-eclampsia, and their babies, benefit from magnesium sulphate? The Magpie Trial: a randomised placebo-controlled trial. *Lancet* 2002, 359(9321):1877-1890.**

**3. Moodley J, Moodley J: Prophylactic anticonvulsant therapy in hypertensive crises of pregnancy - the need for a large randomized trial. *Hypertension in Pregnancy;* 1994, 13:245-252.**

**4. Coetzee EJ, Dommisse J, Anthony J: A randomised controlled trial of intravenous magnesium sulphate versus placebo in the management of women with severe pre-eclampsia. *Br J Obstet Gynaecol* 1998, 105(3):300-303.**

**5. Adeeb N, Ho CM: Comparing magnesium sulphate versus diazepam in the management of severe pre-eclampsia and eclampsia. In: *9th International Congress of the International Society for the Study of Hypertension in Pregnancy: March 15-18. 1994.; Sydney, Australia.*; 1994.**

**6. Adeeb N, Hatta AZ, Shariff J: Comparing magnesium sulphate to diazepam in managing severe pre-eclampsia and eclampsia. In: *10th World Congress of the International Society for the Study of Hypertension in Pregnancy: August 4-8. 1996; Seattle, Washington, USA*; 1996.**

**7. Lucas MJ, Leveno KJ, Cunningham FG: A comparison of magnesium sulfate with phenytoin for the prevention of eclampsia. *N Engl J Med* 1995, 333(4):201-205.**
