# Supplementary material for: Reducing stillbirths: interventions during labour
Source: BMC Pregnancy Childbirth. 2009 May 7;9(Suppl 1):S6. doi: 10.1186/1471-2393-9-S1-S6 (PMC2679412; doi:10.1186/1471-2393-9-S1-S6)
Supplement: Additional file 18 — Web Table 18. Component studies in Duley and Henderson-Smart 2003 meta-analysis: Impact of magnesium sulphate versus diazepam for eclampsia on stillbirth and perinatal mortality. Component studies in Duley and Henderson-Smart 2003 meta-analysis showing impact on stillbirths/perinatal mortality. [file 1471-2393-9-S1-S6-S18.doc]

**Web Table 18. Component studies in Duley and Henderson-Smart 2003 meta-analysis [1]: Impact of magnesium sulphate versus diazepam for eclampsia on stillbirth and perinatal mortality**

| **Source** | **Location and Type of Study** | **Intervention** | **Stillbirths / Perinatal Outcomes** |
| --- | --- | --- | --- |
| 1. Collab Trial 1995 [2] | Africa, Asia and South America. Multicentred.  RCT. N=910 women with eclampsia. | Compared the impact of intervention with MgS04: Either (a) 4/5 g iv over 5 min and 10 g im. Then 5 g im every 4 hr, for 24 hr. Or (b) 4/5 iv over 5 min, then infusion of 1 g/hr for 24 hr. For both (a) and (b), if recurrent convulsions 2 g iv. The control group had diazepam: 10 mg iv bolus. Then infusion of 40 mg/500 ml for 24 hr, rate titrated against conscious level. 20 mg/500 ml for a further 24 hr. For recurrent convulsions, 10 mg iv. | SBR: RR=1.00 (95% CI: 0.69 – 1.44) **[NS]**.  [49/331 vs. 47/317 in intervention and control groups, respectively].  PMR: RR=1.11 (95% CI: 0.84 – 1.46) **[NS]**.  [82/331 vs. 71/317 in intervention and control groups, respectively].  NMR: RR=1.29 (95% CI: 0.79 – 2.09) **[NS]**.  [35/331 vs. 26/317 in intervention and control groups, respectively]. |
| 2. Adeeb et al. 1994 [3, 4] | Malaysia.  RCT. N=39 women (N=11 with eclampsia, N=28 with pre-eclampsia) | Compared the impact of intervention with MgS04: "Pritchard's regime", not described further. The control group was given diazepam: Not described. | SBR: RR=2.57 (95% CI: 0.13 – 52.12) **[NS]**.  [1/6 vs. 0/5 in intervention and control groups, respectively].  NMR: RR=0.83 (95% CI: 0.07 – 10.20) **[NS]**.  [1/6 vs. 1/5 in intervention and control groups, respectively]. |
| 3. Crowther 1990 [5] | Zimbabwe.  RCT. N=51 women with antepartum eclampsia, > 28 weeks gestation, and a live fetus at admission. | Compared the impact of intervention with MgS04: 4 g iv over 3-5 min and 10 g im. Then 5 g im every 4 hr, until 24 hr after delivery. For recurrent convulsions, 2 g iv. The control group had diazepam: 10 mg iv bolus. Then infusion of 80 mg/l for 24 hr, rate titrated against conscious level. 40 mg/l for a further 24 hr. For recurrent convulsions, 10 mg iv. | SBR: RR=0.16 (95% CI: 0.01 – 2.93) **[NS]**.  [0/27 vs. 3/30 in intervention and control groups, respectively].  PMR: RR=0.74 (95% CI: 0.13 – 4.10) **[NS]**.  [2/27 vs. 3/30 in intervention and control groups, respectively].  NMR: RR=5.54 (95% CI: 0.28 – 110.42) **[NS]**.  [2/27 vs. 0/30 in intervention and control groups, respectively]. |
| 4. Duley and Mahomed 1998 [6] | Zimbabwe.  RCT. N=69 women with eclampsia. | Compared the impact of MgS04: Either (a) 4/5 g iv over 5 min and 10 g im. Then 5 g im every 4 hr, for 24 hr. Or (b) 4/5 iv over 5 min, then infusion of 1 g/hr for 24 hr. For both (a) and (b), if recurrent convulsions 2 g iv. The control group had diazepam: 10 mg iv bolus. Then infusion of 40 mg/500 ml for 24 hr, rate titrated against conscious level. 20 mg/500 ml for a further 24 hr. For recurrent convulsions, 10 mg iv. | SBR: RR=0.18 (95% CI: 0.02 – 1.41) **[NS]**.  [1/21 vs. 5/19 in intervention and control groups, respectively].  PMR: RR=0.45 (95% CI: 0.13 – 1.56) **[NS]**.  [3/21 vs. 6/19 in intervention and control groups, respectively]. |

**References**

**1. Duley L, Henderson-Smart D: Magnesium sulphate versus diazepam for eclampsia. *Cochrane Database Syst Rev* 2003(4):CD000127.**

**2. Which anticonvulsant for women with eclampsia? Evidence from the Collaborative Eclampsia Trial. *Lancet* 1995, 345(8963):1455-1463.**

**3. Adeeb N, Ho CM: Comparing magnesium sulphate versus diazepam in the management of severe pre-eclampsia and eclampsia. In: *9th International Congress of the International Society for the Study of Hypertension in Pregnancy: March 15-18. 1994.; Sydney, Australia.*; 1994.**

**4. Adeeb N, Hatta AZ, Shariff J: Comparing magnesium sulphate to diazepam in managing severe pre-eclampsia and eclampsia. In: *10th World Congress of the International Society for the Study of Hypertension in Pregnancy: August 4-8. 1996; Seattle, Washington, USA*; 1996.**

**5. Crowther C: Magnesium sulphate versus diazepam in the management of eclampsia: a randomized controlled trial. *Br J Obstet Gynaecol* 1990, 97(2):110-117.**

**6. Duley L, Mahomed K: Magnesium sulphate in eclampsia. *Lancet* 1998, 351(9108):1061-1062.**
