# Supplementary material for: Reducing stillbirths: interventions during labour
Source: BMC Pregnancy Childbirth. 2009 May 7;9(Suppl 1):S6. doi: 10.1186/1471-2393-9-S1-S6 (PMC2679412; doi:10.1186/1471-2393-9-S1-S6)
Supplement: Additional file 19 — Web Table 19. Component studies in Duley and Henderson-Smart 2003 meta-analysis: Impact of magnesium sulphate versus phenytoin for eclampsia on stillbirth and perinatal mortality. Component studies in Duley and Henderson-Smart 2003 meta-analysis showing impact on stillbirths/perinatal mortality. [file 1471-2393-9-S1-S6-S19.doc]

**Web Table 19. Component studies in Duley and Henderson-Smart 2003 meta-analysis [1]: Impact of magnesium sulphate versus phenytoin for eclampsia on stillbirth and perinatal mortality**

| **Source** | **Location and Type of Study** | **Intervention** | **Stillbirths / Perinatal Outcomes** |
| --- | --- | --- | --- |
| 1. Dommisse 1990 [2] | South Africa (Cape Town).  RCT. N=22 women with antenatal eclampsia and no previous anti-convulsant (1 had phenobarbitone and was entered in error). | Compared the impact of intervention with MgS04: 4 g IV over 20-30 min. Then 1-2 g/hr for 24 hr. The control group had phenytoin: 500 or 1000 mg IV at maximum rate of 50 mg/min. Then 500 mg over 4 hr. 12 hr later, 500 mg over 4 hr.  All women had clonazepam at entry | SBR: RR=1.00 (95% CI: 0.17 – 5.89) **[NS]**.  [2/11 vs. 2/11 in intervention and control groups, respectively].  PMR: RR=1.00 (95% CI: 0.17 – 5.89) **[NS]**.  [2/11 vs. 2/11 in intervention and control groups, respectively].  NMR: RR=not estimable.  [0/11 vs. 0/11 in intervention and control groups, respectively]. |
| 2. Collab Trial 1995 [3] | South Africa and India. 4 centres.  RCT. N=777 women with clinical diagnosis of eclampsia. 76% allocated MgSO4 had an anti-convulsant before trial entry, and 80% allocated phenytoin. 19% postpartum. | Compared the impact of intervention with MgS04: Either (a) 4 g IV over 5 min and 10 g IM. Then 5 g IM every 4 hr for 24 hr. Or (b) 4 g IV over 5 min, then infusion of 1 g/hr for 24 hr. For both (a) and (b), if recurrent convulsions 2 g IV. The control group had phenytoin: diazepam 10 mg IV for control of seizures (PRN). 1 g phenytoin IV over 20 min, then 100 mg every 6 hr for 24 hr.  Clinical monitoring alone, no serum monitoring. | SBR: RR=0.82 (95% CI: 0.60 – 1.13) **[NS]**.  [55/314 vs. 70/329 in intervention and control groups, respectively].  PMR: RR=0.85 (95% CI: 0.66 – 1.09) **[NS]**.  [82/314 vs. 101/329 in intervention and control groups, respectively].  NMR: RR=0.95 (95% CI: 0.59 – 1.53) **[NS]**.  [29/314 vs. 32/329 in intervention and control groups, respectively]. |

**References**

**1. Duley L, Henderson-Smart D: Magnesium sulphate versus phenytoin for eclampsia. *Cochrane Database Syst Rev* 2003(4):CD000128.**

**2. Dommisse J: Phenytoin sodium and magnesium sulphate in the management of eclampsia. *Br J Obstet Gynaecol* 1990, 97(2):104-109.**

**3. Which anticonvulsant for women with eclampsia? Evidence from the Collaborative Eclampsia Trial. *Lancet* 1995, 345(8963):1455-1463.**
