# Supplementary material for: Reducing stillbirths: interventions during labour
Source: BMC Pregnancy Childbirth. 2009 May 7;9(Suppl 1):S6. doi: 10.1186/1471-2393-9-S1-S6 (PMC2679412; doi:10.1186/1471-2393-9-S1-S6)
Supplement: Additional file 20 — Web Table 20. Component studies in Duley and Gulmezoglu 2000 meta-analysis: Impact of magnesium sulphate vs. lytic cocktail on stillbirth and neonatal mortality. Component studies in Duley and Gulmezoglu 2000 meta-analysis showing impact on stillbirths/perinatal mortality. [file 1471-2393-9-S1-S6-S20.doc]

**Web Table 20. Component studies in Duley and Gulmezoglu 2000 [1] meta-analysis: Impact of magnesium sulphate vs. lytic cocktail on stillbirth and neonatal mortality**

| **Source** | **Location and Type of Study** | **Intervention** | **Stillbirths / Perinatal Outcomes** |
| --- | --- | --- | --- |
| 1. Bhalla et al. 1994 [2] | India.  RCT. N=91 women with eclampsia. | Compared the impact of intervention with MgSO4: 4 g IV (20% solution) + 8 g IM (50% solution) loading dose, then 4 g 4 hourly until 24 hours after delivery. If recurrent fits, 1.5 g IV. The comparison group had lytic cocktail: pethidine. promethazine and chlorpromazine | SBR: RR=1.04 (95% CI: 0.45 – 2.40) **[NS]**.  [9/39 vs. 8/36 in intervention and comparison groups, respectively].  NMR: RR=0.55 (95% CI: 0.14 – 2.15) **[NS]**.  [3/39 vs. 5/36 in intervention and comparison groups, respectively]. |
| 2. Jacob et al. 1995 [3] | India.  RCT. N=108 women with eclampsia. India. | Compared the impact of intervention with MgSO4: 4 g IV + 10 g IM loading dose, then 5 g 4 hourly up to 24 hours after delivery vs. lytic cocktail (comparison group): 100 mg pethidine + 25 mg chlorpromazine IV and 50 mg chlorpromazine + 25 mg promethazine IM loading dose. 100mg pethidine in 1 litre 20% dextrose over 24 hours, 25 mg promethazine 4 hourly, 50 mg chlorpromazine 8 hourly for 48 hours. | SBR: RR=0.06 (95% CI: 0.00 – 1.03) **[NS]**.  [0/50 vs. 8/52 in intervention and comparison groups, respectively].  NMR: RR=0.28 (95% CI: 0.06 – 1.26) **[NS]**.  [2/51 vs. 8/57 in intervention and control groups, respectively]. |

**References**

**1. Duley L, Gulmezoglu AM: Magnesium sulphate versus lytic cocktail for eclampsia. *Cochrane Database of Systematic Reviews* 2000(3):CD002960.**

**2. Bhalla AK, Dhall GI, Dhall K: A safer and more effective treatment regimen for eclampsia. *Aust N Z J Obstet Gynaecol* 1994, 34(2):144-148.**

**3. Jacob S, Gopalakrishnan K, Lalitha K: Standardised clinical trial of magnesium sulphate regime in comparison with M.K.K. Menon's lytic cocktail regime in the management of eclampsia. In: *Proceedings of the 27th British Congress of Obstetrics and Gynaecology: 4-7 July 1995; Dublin*; 4-7 July 1995.**
