# Supplementary material for: Reducing stillbirths: interventions during labour
Source: BMC Pregnancy Childbirth. 2009 May 7;9(Suppl 1):S6. doi: 10.1186/1471-2393-9-S1-S6 (PMC2679412; doi:10.1186/1471-2393-9-S1-S6)
Supplement: Additional file 21 — Web Table 21. Component studies in Doyle et al. 2007 meta-analysis: Impact of magnesium sulphate as a neuroprotective agent in women at risk of pre-term birth on fetal death. Component studies in Doyle et al. 2007 meta-analysis showing impact on stillbirths/perinatal mortality. [file 1471-2393-9-S1-S6-S21.doc]

**Web Table 21. Component studies in Doyle et al. 2007 [1] meta-analysis: Impact of magnesium sulphate as a neuroprotective agent in women at risk of pre-term birth on fetal death**

| **Source** | **Location and Type of Study** | **Intervention** | **Stillbirths / Perinatal Outcomes** |
| --- | --- | --- | --- |
| **Neuroprotective intent** |  |  |  |
| 1. Crowther 2003 [2] | Australia and New Zealand. 16 tertiary hospitals.  RCT. N=1062 women (1255 fetuses) < 30 weeks' gestation likely to deliver within 24 hours. | Compared the impact of active treatment - infusion of 4 g magnesium sulphate over 20 minutes, then 1 g/hour until delivery or for 24 hours, whichever came first (intervention) vs. placebo group - equal volume of 0.9% saline (controls). | Fetal death (miscarriage + SB): RR=0.81 (95% CI: 0.34 – 1.95) **[NS]**.  [9/629 vs. 11/626 in intervention and control groups, respectively]. |
| 1. Marret 2006 [3] | France. Eighteen tertiary hospitals.  RCT. N=564 women (688 fetuses) in labour < 33 weeks' gestation (N=286 women intervention group, N= 278 controls). | Compared the impact of 4 g magnesium sulphate over 30 minutes (intervention) vs. placebo (isotonic 0.9% saline) (controls). | Fetal death (miscarriage + SB): RR=0.64 (95% CI: 0.11 – 3.78) **[NS]**.  [2/352 vs. 3/336 in intervention and control groups, respectively]. |
| 1. Mittendorf 2002 [4] | USA.  RCT. N=149 women (165 fetuses) in pre-term labour, with or without premature rupture of the membranes (in the tocolytic arm, N=46 intervention group, N=46 other tocolysis. In the women ineligible for tocolysis, N=29 intervention group, N=28 controls). | Compared the impact among women eligible for aggressive tocolysis (cervix <= 4 cm dilation) of magnesium sulphate vs.'other' tocolysis. Among women not eligible for tocolysis (cervix > 4 cm dilation), compared the impact of neuroprotective magnesium sulphate vs. saline control. | Fetal death (miscarriage + SB): RR=2.90 (95% CI: 0.12 – 68.50) **[NS]**.  [1/30 vs. 0/29 in the magnesium and control groups, respectively. |
| **Other intent** | | | |
| 1. Magpie 2006 [5] | 19 countries. 125 centres.  RCT. N=1544 women (1593 fetuses) < 37 weeks' gestation with severe pre-eclampsia. | Compared the impact of active treatment with magnesium sulphate dose 4 g intravenously over 10-15 minutes, followed by either 1 g/hour intravenously for 24 hours, or by 5 g every 4 hours intramuscularly for 24 hours. The controls were given a placebo intravenously. | Fetal death (miscarriage + SB): RR=1.00 (95% CI: 0.78 – 1.27) **[NS]**.  [111/798 vs. 111/795 in intervention and control groups, respectively]. |
| 1. Mittendorf 2002 [4] | USA.  RCT. N=149 women (165 fetuses) in pre-term labour, with or without premature rupture of the membranes. | Compared the impact among women eligible for aggressive tocolysis (cervix <= 4 cm dilation) of magnesium sulphate vs.'other' tocolysis. Among women not eligible for tocolysis (cervix > 4 cm dilation), compared the impact of neuroprotective magnesium sulphate vs. saline control. | Fetal death (miscarriages + SB): RR=not estimable.  [0/55 vs. 0/51 in the magnesium and control groups, respectively]. |

**References**

1. Doyle LW, Crowther CA, Middleton P, Marret S: **Magnesium sulphate for women at risk of preterm birth for neuroprotection of the fetus**. *Cochrane Database Syst Rev* 2007(3):CD004661.

2. Crowther CA, Hiller JE, Doyle LW, Haslam RR: **Effect of magnesium sulfate given for neuroprotection before preterm birth: a randomized controlled trial**. *JAMA* 2003, **290**(20):2669-2676.

3. Marret S, Marpeau L, Zupan-Simunek V, Eurin D, Leveque C, Hellot MF, Benichou J: **Magnesium sulphate given before very-preterm birth to protect infant brain: the randomised controlled PREMAG trial***. *BJOG* 2007, **114**(3):310-318.

4. Mittendorf R, Dambrosia J, Pryde PG, Lee KS, Gianopoulos JG, Besinger RE, Tomich PG: **Association between the use of antenatal magnesium sulfate in preterm labor and adverse health outcomes in infants**. *Am J Obstet Gynecol* 2002, **186**(6):1111-1118.

5. **The Magpie Trial: a randomised trial comparing magnesium sulphate with placebo for pre-eclampsia. Outcome for children at 18 months**. *BJOG* 2007, **114**(3):289-299.
