# Supplementary material for: Reducing stillbirths: interventions during labour
Source: BMC Pregnancy Childbirth. 2009 May 7;9(Suppl 1):S6. doi: 10.1186/1471-2393-9-S1-S6 (PMC2679412; doi:10.1186/1471-2393-9-S1-S6)
Supplement: Additional file 22 — Web Table 22. Component studies in Crowther et al. 2002 meta-analysis: Impact of magnesium sulphate in threatened pre-term labour on fetal deaths. Component studies in Crowther et al. 2002 meta-analysis showing impact on stillbirths/perinatal mortality. [file 1471-2393-9-S1-S6-S22.doc]

**Web Table 22. Component studies in Crowther et al. 2002 [1] meta-analysis: Impact of magnesium sulphate in threatened pre-term labour on fetal deaths**

| **Source** | **Location and Type of Study** | **Intervention** | **Stillbirths / Perinatal Outcomes** |
| --- | --- | --- | --- |
| 1. Armson 1992 [2] | USA (Pennsylvania). Single centre.  Non-placebo-controlled RCT. N=15 women (N=8 intervention group, N=7 controls). | Compared the impact of magnesium sulphate (intervention) vs. ritodrine (controls). Dose of the intervention: MgSO4 - initial treatment 6g IV MgSO4 over 30 minutes. Maintenance at 2g/hour. Increased by 0.5g/hour every 30 minutes until tocolysis achieved, a maximum of 4g/hour attained or unacceptable side effects. The control dose (ritodrine) - initial treatment 50mcg/min. Maintenance: Increased by 50mcg/min at 15 minute intervals until tocolysis attained, a maximum of 350mcg/min, or unacceptable side effects.  If tocolysis was successful the infusion rate was maintained at the lowest effective dose for 12 hours. | Fetal deaths: RR=not estimable.  [0/8 vs. 0/7 in intervention and control groups, respectively]. |
| 2. Beall 1985 [3] | USA (Los Angeles) Single centre.  Non-placebo-controlled RCT. N=176 women (N=46 intervention group, N=45 control # 1, N=40 control # 2). | Compared the impact of magnesium sulphate (intervention) vs. ritodrine (control # 1) vs. terbutaline (control # 2).  Dose: MgSO4 - initial treatment 4g IV MgSO4. Maintenance at 1.5g/hour. Increased by 0.5g/hour every 30 minutes until tocolysis achieved, a maximum of 3.5g/hour attained or unacceptable side effects. Ritodrine - initial treatment 100mcg/min. Maintenance increased by 50% every 10 minutes until contractions ceased, a maximum of 350mcg/min attained or if unacceptable side effects. Terbutaline - initial treatment 20mcg/min. Maintenance increased by 50% every 10 minutes until contractions ceased, a maximum of 70mcg/min or if unacceptable side effects.  If tocolysis was successful, the infusion rate was maintained for 12 hours. One half hour prior to completion of intravenous therapy, patients commenced oral terbutaline and discharged home 48 hours later on 2.5mg every 4 hours. | Fetal deaths: RR=not estimable.  [0/46 vs. 0/85 in intervention and combined controls, respectively]. |
| 3. Cotton 1984 [4] | USA (Los Angeles). Single centre.  RCT. N=56 women (N=16 magnesium sulphate group, N=19 terbutaline, and N=19 dextrose group). | Compared the impact of magnesium sulphate (intervention) vs. terbutaline vs. dextrose. Dose: MgSO4 - initial treatment 4g IV. Maintenance: 2g/hour. Terbutaline - initial treatment: 9.2mcg/min IV. Maintenance: increased 5mcg/min to 25.3mcg/min. Dextrose: 125ml/hour.  Therapy continued for 12 hours after contractions stopped. Stopped if cervix >7cm, amnionitis or side effects | No fetal deaths: RR=not estimable. |
| 4. Cox 1990 [5] | USA (Dallas, Texas). Single centre.  RCT. N=156 women between 1987-1989 (N=76 intervention group, N=80 controls) | Compared the impact of magnesium sulphate (intervention) vs. saline control. Dose: MgSO4 - initial treatment 4g IV. Maintenance: 2g/hour. Increasing to3g/hour if still contracting after >1 hour. Duration: therapy continued for 24 hours. Saline: 80ml/hour for 24 hours. | Fetal deaths: RR=5.70 (95% CI: 0.28 – 116.87) **[NS]**.  [2/78 vs. 0/89 in intervention and control groups, respectively]. |
| 5. Fox 1993 [6] | USA (Jackson, Mississippi). Single centre.  Non-placebo-controlled RCT. N=101 women (N=45 intervention group, N=45 controls). | Compared the impact of magnesium sulphate (intervention) vs. control group. Dose: MgSO4 - initial treatment: 4g IV as a bolus. Maintenance: 2-4g/hour until uterine quiescence obtained. Duration: after uterine quiescence oral magnesium until 37 weeks' gestation. The control group had conservative management with hydration, sedation and observation, and underwent identical evaluation compared to MgSO4 group but labour allowed to progress naturally. | Fetal deaths: RR – not estimable.  [0/45 vs. 0/45 in intervention and control groups, respectively]. |
| 6. Glock 1993 [7] | USA (Orlando, Florida). Single centre.  Non-placebo-controlled RCT. N=100 women between 1991-1992 (N=41 intervention group, N=39 nifedipine). | Compared the impact of magnesium sulphate (intervention) vs. nifedipine (controls). Dose: MgSO4 - initial treatment: 6g IV over 30 minutes. Maintenance: 2-4g/hour as needed to keep the uterus quiescent for 24 hours. Duration: after 24 hours of arrest of contractions the patient was weaned at a rate of 0.5g/hour every 4-6 hours. Patients were started on 5mg oral terbutaline every 6 hours until 34 weeks gestation completed. Nifedipine - initial treatment: 10mg sublingually, repeated every 20 minutes up to a maximum of 40mg in the first hour of treatment. If contractions stopped, given 20mg oral nifedipine 4 hourly for 48 hours. Continued on 10mg nifedipine 8 hourly until 34 completed weeks. | Fetal deaths: RR=not estimable. [0/41 vs. 0/39 in intervention and control groups, respectively]. |
| 7. Morales 1993 [8] | USA (Orlando, Florida). Single centre.  Non-placebo RCT. N=114 women between 1988-1989 (N=58 intervention group, N=56 controls). | Compared the impact of magnesium sulphate (intervention) vs. indomethacin (controls). Dose: MgSO4 - initial treatment 6g IV bolus over 30 minutes. Maintenance: 2g-5g/hour. Indomethacin - initial treatment 100mg rectal suppository. Maintenance: 25mg orally every 4 hours for 48 hours. If regular contractions persisted 1-2 hours after the initial 100mg suppository this was repeated.After cessation of contractions for 12 hours all women received oral terbutaline 5mg every 6 hours for prophylaxis against recurrent pre-term labour. | Fetal deaths: RR=not estimable.  [0/59 vs. 0/58 in intervention and control groups, respectively]. |

1. Crowther CA, Hiller JE, Doyle LW: **Magnesium sulphate for preventing preterm birth in threatened preterm labour**. *Cochrane Database Syst Rev* 2002(4):CD001060.

2. Armson BA, Samuels P, Miller F, Verbalis J, Main EK: **Evaluation of maternal fluid dynamics during tocolytic therapy with ritodrine hydrochloride and magnesium sulfate**. *Am J Obstet Gynecol* 1992, **167**(3):758-765.

3. Beall MH, Edgar BW, Paul RH, Smith-Wallace T: **A comparison of ritodrine, terbutaline, and magnesium sulfate for the suppression of preterm labor**. *Am J Obstet Gynecol* 1985, **153**(8):854-859.

4. Cotton DB, Strassner HT, Hill LM, Schifrin BS, Paul RH: **Comparison of magnesium sulfate, terbutaline and a placebo for inhibition of preterm labor. A randomized study**. *J Reprod Med* 1984, **29**(2):92-97.

5. Cox SM, Sherman ML, Leveno KJ: **Randomized investigation of magnesium sulfate for prevention of preterm birth**. *Am J Obstet Gynecol* 1990, **163**(3):767-772.

6. Fox MD, Allbert JR, McCaul JF, Martin RW, McLaughlin BN, Morrison JC: **Neonatal morbidity between 34 and 37 weeks' gestation**. *J Perinatol* 1993, **13**(5):349-353.

7. Glock JL, Morales WJ: **Efficacy and safety of nifedipine versus magnesium sulfate in the management of preterm labor: a randomized study**. *Am J Obstet Gynecol* 1993, **169**(4):960-964.

8. Morales WJ, Madhav H: **Efficacy and safety of indomethacin compared with magnesium sulfate in the management of preterm labor: a randomized study**. *Am J Obstet Gynecol* 1993, **169**(1):97-102.
