# Supplementary material for: Reducing stillbirths: interventions during labour
Source: BMC Pregnancy Childbirth. 2009 May 7;9(Suppl 1):S6. doi: 10.1186/1471-2393-9-S1-S6 (PMC2679412; doi:10.1186/1471-2393-9-S1-S6)
Supplement: Additional file 23 — Web Table 23. Component studies in Crowther and Moore 1998: Impact of magnesium maintenance therapy for preventing pre-term birth on perinatal mortality. Component studies in Crowther and Moore 1998 showing impact on stillbirths/perinatal mortality. [file 1471-2393-9-S1-S6-S23.doc]

**Web Table 23. Component studies in Crowther and Moore 1998 [1]: Impact of magnesium maintenance therapy for preventing pre-term birth on perinatal mortality**

| **Source** | **Location and Type of Study** | **Intervention** | **Stillbirths / Perinatal Outcomes** |
| --- | --- | --- | --- |
| 1. Ricci et al. 1991 [2] | USA (Miami). Tertiary care (hospital setting).  RCT. N=75 women of low socio-economic status, at 24-34 weeks of gestation of a singleton pregnancy, who had been 12 hours without contractions following threatened pre-term labour, which was treated with intravenous magnesium sulphate (N=25 in each group). | Compared the impact of 535 mg SLOW MAG (enteric-coated magnesium chloride) every 4 hours (intervention) vs. 10 mg oral ritodrine every 2 hours for 24 hours, then 20 mg every 4 hours vs. no treatment (controls).  Duration of treatment unclear | SBR: RR=not estimable.  [0/25 in the SLOW MAG vs. ritodrine groups, respectively].  Death before discharge among live-born infants: RR=5.00 (95% CI: 0.25 – 99.16) **[NS]**.  [2/25 vs. 0/25 in magnesium vs. ritodrine groups, respectively]. |

**References**

1. Crowther CA, Moore V: **Magnesium maintenance therapy for preventing preterm birth after threatened preterm labour**. *Cochrane Database of Systematic Reviews* 1998(1):CD000940.

2. Ricci JM, Hariharan S, Helfgott A, Reed K, O'Sullivan MJ: **Oral tocolysis with magnesium chloride: a randomized controlled prospective clinical trial**. *Am J Obstet Gynecol* 1991, **165**(3):603-610.
