# Supplementary material for: Reducing stillbirths: interventions during labour
Source: BMC Pregnancy Childbirth. 2009 May 7;9(Suppl 1):S6. doi: 10.1186/1471-2393-9-S1-S6 (PMC2679412; doi:10.1186/1471-2393-9-S1-S6)
Supplement: Additional file 24 — Web Table 24. Component studies in Say et al. 2003 meta-analysis: Impact of maternal oxygen therapy on perinatal mortality. Component studies in Say et al. 2003 meta-analysis showing impact on stillbirths/perinatal mortality. [file 1471-2393-9-S1-S6-S24.doc]

**Web Table 24. Component studies in Say et al. 2003 [1] meta-analysis: Impact of maternal oxygen therapy on perinatal mortality**

| **Source** | **Location and Type of Study** | **Intervention** | **Stillbirths / Perinatal Outcomes** |
| --- | --- | --- | --- |
| 1. Battaglia et al. 1992 [2] | Italy.  RCT. N=36 women at 26-34 weeks' gestation. | Compared the impact on perinatal mortality of traditional management + 55% humidified oxygen via a face mask 24 hours a day (intervention) vs. traditional management only (bed rest, anti-hypertensives where necessary). | PMR: RR=0.43 (95% CI: 0.19 – 0.95).  [5/17 vs. 13/19 in intervention and control groups, respectively]. |
| 2. Johanson et al. 1995 [3, 4] | South Africa.  Non-blinded RCT. N=26 women with singleton pregnancy between 24-30 weeks' gestation and with absent end-diastolic flow in the umbilical artery. | Compared the impact on perinatal mortality of bed rest and continuous 40% oxygen by a face mask until delivery or fetal demise (intervention) vs. bed rest only which was the standard management (controls). | PMR: RR=0.38 (95% CI: 0.13 – 1.11) **[NS]**.  [3/13 vs. 8/13 in intervention and control groups, respectively]. |
| 3. Lindow et al. 2002 [5-7] | UK and South Africa.  Double-blind RCT. N=32 women with impaired fetal growth diagnosed by doppler studies between 24-30 weeks of gestation. | Compared the impact on perinatal mortality of continuous 40% humidified oxygen by a face mask at 8L/min until delivery (intervention) vs. continuous humidified air by a face mask at 8L/min giving a 40% of gas (controls). | PMR: RR=0.70 (95% CI: 0.36 – 1.37) **[NS]**.  [7/16 vs. 10/16 in intervention and control groups, respectively]. |

**References**

1. Say L, Gulmezoglu AM, Hofmeyr GJ: **Maternal oxygen administration for suspected impaired fetal growth**. *Cochrane Database Syst Rev* 2003(1):CD000137.

2. Battaglia C, Artini PG, D'Ambrogio G, Galli PA, Segre A, Genazzani AR: **Maternal hyperoxygenation in the treatment of intrauterine growth retardation**. *Am J Obstet Gynecol* 1992, **167**(2):430-435.

3. Johanson R, Lindow SW, van der Elst C, Jaquire Z, van der Westhuizen S, Tucker A: **A prospective randomised comparison of the effect of continuous O2 therapy and bedrest on fetuses with absent end-diastolic flow on umbilical artery Doppler waveform analysis**. *Br J Obstet Gynaecol* 1995, **102**(8):662-665.

4. Johanson R, Lindow S, van der Elst C, van der Westhuizen S, Tucker A, Jaquire Z: **RCT of maternal oxygen therapy for in-utero asphyxia.** In: *Proceedings of 2nd International Scientific Meeting of the Royal College of Obstetricians and Gynaecologists: 1993 Sept 7-10.; Hong Kong*; 1993 Sept 7-10.

5. Lindow S, Mantell G, Anthony J, Coetzee E: **A double-blind randomised controlled trial of continuous oxygen therapy for compromised fetuses**. *Journal of Obstetrics and Gynaecology;* 2002, **22**:S35.

6. Lindow SW, Mantel GD, Anthony J, Coetzee EJ: **A double-blind randomised controlled trial of continuous oxygen therapy for compromised fetuses**. *BJOG* 2002, **109**(5):509-513.

7. Lindow S, Mantel G, Anthony J, Coetzee E, Pharoah P: **A double blind randomised controlled trial of continuous oxygen therapy for compromised fetuses**. *Journal of Perinatal Medicine;* 2001, **29**(Suppl 1):123.
