# Supplementary material for: Reducing stillbirths: interventions during labour
Source: BMC Pregnancy Childbirth. 2009 May 7;9(Suppl 1):S6. doi: 10.1186/1471-2393-9-S1-S6 (PMC2679412; doi:10.1186/1471-2393-9-S1-S6)
Supplement: Additional file 25 — Web Table 25. Component studies in Hofmeyr 1998 meta-analysis: Impact of amnioinfusion for potential or suspected umbilical cord compression on perinatal mortality. Component studies in Hofmeyr 1998 meta-analysis showing impact on stillbirths/perinatal mortality. [file 1471-2393-9-S1-S6-S25.doc]

**Web Table 25. Component studies in Hofmeyr 1998 [1] meta-analysis: Impact of amnioinfusion for potential or suspected umbilical cord compression on perinatal mortality**

| **Source** | **Location and Type of Study** | **Intervention** | **Stillbirths / Perinatal Outcomes** |
| --- | --- | --- | --- |
| 1. Chauhan et al. 1992 [2] | USA.  RCT. N=38 women (N=21 intervention group, N=17 controls). | Compared the impact of amnioinfusion with 250 ml normal saline at room temperature, followed if necessary by repeat infusions of 100 ml until an amniotic fluid index of 5 cm or more achieved (intervention) vs. no amnioinfusion (controls). | PMR: RR=not estimable.  [0/21 vs. 0/17 in intervention and control groups, respectively]. |
| 2. MacGregor et al. 1991 [3] | USA.  RCT. N=35 women (N=19 intervention group, N=16 controls). | Compared the impact of amnioinfusion with Ringer's solution warmed to 37 degrees centigrade at 10 ml per minute for 1 hour and continued if variable decelerations persisted until decelerations ceased or 800 ml had been infused, followed by infusion at 3 ml per minute till delivery (intervention) vs. no amnioinfusion (controls). | PMR: RR=not estimable.  [0/19 vs. 0/16 in intervention and control groups, respectively]. |
| 3. Miyazaki et al. 1985 [4] | USA.  RCT. N=96 women (N=49 intervention group, N=47 controls). | Compared the impact of amnioinfusion with normal saline until decelerations resolved plus 250 ml (maximum 800 ml) and repeated if necessary (intervention) vs. no amnioinfusion (controls). | PMR: RR=not estimable.  [0/49 vs. 0/47 in intervention and control groups, respectively]. |
| 4. Nageotte et al. 1985 [5] | USA.  RCT. N=61 women (N=29 intervention group, N=32 controls). | Compared the impact of intrapartum amnioinfusion with normal saline warmed to 37 degrees centigrade at 10 ml per minute for 1 hour (repeated if large fluid loss occurred subsequently), followed by infusion at 3 ml per minute (intervention) vs. no amnioinfusion (controls).  All participants had intrauterine pressure catheter inserted as early in labour as possible. | PMR: RR=0.55 (95% CI: 0.05 – 5.77) **[NS]**.  [1/29 vs. 2/32 in intervention and control groups, respectively]. |
| 5. Nageotte et al. 1991 [6, 7] | USA.  RCT. N=76 (N=50 intervention (combined) group, N=26 controls). | Compared the impact between warmed saline (93-96 degrees Fahrenheit) group and room temperature saline (68-72 degrees Fahrenheit) group (intervention combined) vs. no amnioinfusion (controls).  Amnioinfusion rate was 10 ml per minute for 1 hour, then 3 ml per minute until delivery, briefly stopped every 30 minutes to evaluate resting tone. | PMR: RR=1.59 (95% CI: 0.07 – 37.68) **[NS]**.  [1/50 vs. 0/26 in intervention and control groups, respectively]. |
| 6. Owen et al. 1990 [8] | USA.  RCT. N=100 women (N=43 intervention group, N=57 controls). | Compared the impact of amnioinfusion with saline warmed to 37 degrees centigrade at 10 ml per minute for 1 hour, then 3 ml per minute (intervention) vs. no amnioinfusion (controls).  All women had intrauterine pressure transducers. Amnioinfusion was performed through a second intrauterine catheter. | PMR: RR=not estimable.  [0/43 vs. 0/57 in intervention and control groups, respectively]. |
| 7. Strong et al. 1990 [9] | USA.  RCT. N=60 (N=30 intervention group, N=30 controls). | Compared the impact of amnioinfusion of 250 ml saline warmed to 37 degrees centigrade at 10 to 20 ml per minute, repeated until an amniotic fluid index of 8 cm was reached and if the amniotic fluid index fell below 8 cm on subsequent hourly ultrasound examinations (intervention) vs. no amnioinfusion (controls). | PMR: RR=not estimable.  [0/30 in both the groups]. |
| 8. Wu 1989 [10] | China  RCT. N=118 women (N=60 intervention group, N=58 controls). | Compared the impact of amnioinfusion with normal saline warmed to 37 degrees centigrade, 500 ml at 15-20 ml per minute followed by slow or intermittent infusion (intervention) vs. no amnioinfusion (controls). | PMR: RR=0.19 (95% CI: 0.01 – 3.94) **[NS]**.  [0/60 vs. 2/58 in intervention and control groups, respectively]. |

**References**

**1. Hofmeyr G: Amnioinfusion for potential or suspected umbilical cord compression in labour. *Cochrane Database of Systematic Reviews;* 1998(1):CD000013.**

**2. Chauhan SP, Rutherford SE, Hess LW, Morrison JC: Prophylactic intrapartum amnioinfusion for patients with oligohydramnios. A prospective randomized study. *J Reprod Med* 1992, 37(9):817-820.**

**3. MacGregor SN, Banzhaf WC, Silver RK, Depp R: A prospective, randomized evaluation of intrapartum amnioinfusion. Fetal acid-base status and cesarean delivery. *J Reprod Med* 1991, 36(1):69-73.**

**4. Miyazaki FS, Nevarez F: Saline amnioinfusion for relief of repetitive variable decelerations: a prospective randomized study. *Am J Obstet Gynecol* 1985, 153(3):301-306.**

**5. Nageotte MP, Freeman RK, Garite TJ, Dorchester W: Prophylactic intrapartum amnioinfusion in patients with preterm premature rupture of membranes. *Am J Obstet Gynecol* 1985, 153(5):557-562.**

**6. Nageotte MP, Bertucci L, Towers CV, Lagrew DC, Mondanlau H: Prophylactic amnioinfusion in pregnancies complicated by oligohydramnios or thick meconium: a prospective study. In: *Proceedings of 9th Annual Meeting of the Society of Perinatal Obstetricians: 1989 February 1-4.; New Orleans, Louisiana.*; 1989 February 1-4.**

**7. Nageotte MP, Bertucci L, Towers CV, Lagrew DL, Modanlou H: Prophylactic amnioinfusion in pregnancies complicated by oligohydramnios: a prospective study. *Obstet Gynecol* 1991, 77(5):677-680.**

**8. Owen J, Henson BV, Hauth JC: A prospective randomized study of saline solution amnioinfusion. *Am J Obstet Gynecol* 1990, 162(5):1146-1149.**

**9. Strong TH, Jr., Hetzler G, Sarno AP, Paul RH: Prophylactic intrapartum amnioinfusion: a randomized clinical trial. *Am J Obstet Gynecol* 1990, 162(6):1370-1374; discussion 1374-1375.**

**10. Wu BT: [Intrapartum amnio-infusion in patients with oligohydramnios]. *Zhonghua Fu Chan Ke Za Zhi* 1989, 24(1):2-4, 57.**
