# Supplementary material for: Reducing stillbirths: interventions during labour
Source: BMC Pregnancy Childbirth. 2009 May 7;9(Suppl 1):S6. doi: 10.1186/1471-2393-9-S1-S6 (PMC2679412; doi:10.1186/1471-2393-9-S1-S6)
Supplement: Additional file 26 — Web Table 26. Component studies in Hofmeyr 2002 meta-analysis: Impact of amnioinfusion for meconium-stained liquor on perinatal mortality. Component studies in Hofmeyr 2002 meta-analysis showing impact on stillbirths/perinatal mortality. [file 1471-2393-9-S1-S6-S26.doc]

**Web Table 26. Component studies in Hofmeyr 2002 [1] meta-analysis: Impact of amnioinfusion for meconium-stained liquor on perinatal mortality**

| **Source** | **Location and Type of Study** | **Intervention** | **Stillbirths / Perinatal Outcomes** |
| --- | --- | --- | --- |
| **Standard peripartum surveillance** | | | |
| 1. Adam et al. 1989. [2] | USA  RCT. N=35 women (N=17 intervention group, N=18 controls). | Compared the impact of single amnioinfusion with 1000ml (intervention) vs. no amnioinfusion (controls). | PMR: RR=not estimable.  [0/17 vs. 0/18 in intervention and control groups, respectively]. |
| 2. Cialone et al. 1994 [3, 4] | USA.  RCT. N=113 women (N=54 intervention group, N=59 controls). | Compared the impact of amnioinfusion of room temperature normal saline 600ml over 1 hour followed by 150ml per hour (intervention) vs. no amnioinfusion (controls). | PMR: RR=not estimable.  [0/47 vs. 0/58 in intervention and control groups, respectively]. |
| 3. Hofmeyr et al. 1998 [5-7] | South Africa. Four academic hospitals.  RCT. N=352 women (N=176 intervention group, N=176 controls). | Compared the impact of amnioinfusion via an Intran or Nelaton intrauterine catheter: 800ml normal saline at 15ml per minute, then maintenance of 3ml per minute (intervention) vs. no amnioinfusion (controls).  Electronic fetal heart rate monitoring in most cases. One woman in the control group received amnioinfusion. Analysis was by intention to treat. | PMR: RR=not estimable.  [0/164 vs. 0/163 in intervention and control groups, respectively]. |
| 4. Macri et al. 1992 [8, 9] | USA.  RCT. N=170 women (N=85 intervention group, N=85 controls). | Assessed the effects of amnioinfusion with 500ml warmed saline over 20-30 minutes followed by 250-500ml as required to maintain a 4-quadrant amniotic fluid index above 10cm (intervention) vs. no amnioinfusion (controls). | PMR: RR=not estimable.  [0/85 in both the groups]. |
| 5. Moodley et al. 1998 [10] | South Africa (Durban).  RCT. N=60 women analyzed between January to April 1993. | Compared the impact of amnioinfusion with normal saline 10-15ml per minute by gravity via a central venous manometer set and a size 8 nasogastric infant feeding tube (1 litre over 4 hours) (intervention) vs. standard care (controls); continuous fetal heart rate monitoring. | PMR: RR=not estimable.  [0/30 in both the groups]. |
| 6. Sadovsky et al. 1989 [11, 12] | USA.  RCT. N=40 women (N=19 intervention group, N=21 controls). | Compared the impact of amnioinfusion with saline 600ml over 1 hour then 180ml per hour (intervention) vs. no amnioinfusion (controls). | PMR: RR=not estimable.  [0/19 vs. 0/21 in intervention and control groups, respectively]. |
| 7. Wenstrom et al. 1989 [13] | USA.  RCT. N=85 women (N=41 intervention group, N=44 controls). | Compared the impact of amnioinfusion of 1000ml over 20-40 minutes, repeated 6-hourly (intervention) vs. no amnioinfusion (controls). | PMR: RR=not estimable.  [0/41 vs. 0/44 in intervention and control groups, respectively]. |
| **Limited peripartum surveillance** | | | |
| 8. Mahomed et al. 1998 [14] | Zimbabwe.  RCT. N=661 women (N=325 intervention group, N=336 controls). | Compared the impact of intervention of transcervical amnioinfusion using size 8 nasogastric tube. Normal saline 500ml infused over 30 minutes, then 500ml at 2ml per minute. The control group received no amnioinfusion.  Allocation not blinded. Level of intrapartum surveillance limited by number of midwives in a busy labour ward. Fetal heart rate auscultated every 30 minutes using Pinard stethoscope or hand-held doptone fetal heart rate detector. Suctioning of the airways at delivery by attending midwives. | PMR: RR=0.34 (95% CI: 0.11 – 1.06) **[NS]**.  [4/324 vs. 12/335 in intervention and control groups, respectively]. |

**References**

1. Hofmeyr GJ: **Amnioinfusion for meconium-stained liquor in labour**. *Cochrane Database Syst Rev* 2002(1):CD000014.

2. Adam K, Cano L, Moise KJ: **The effect of intrapartum amnioinfusion on the outcome of the fetus with heavy meconium stained amniotic fluid.** In: *Proceedings of 9th Annual Meeting of the Society of Perinatal Obstetricians: 1989; New Orleans, Louisiana, U.S.A.*; 1989.

3. Cialone PR, Abramowicz JS, Ryan RM, Sinkin RA, Sherer DM: **Markedly significant decrease in neonatal morbidity associated with amnioinfusion for labor complicated by particulate meconium**. *Am J Obstet Gynecol;* 1993, **168**:319.

4. Cialone PR, Sherer DM, Ryan RM, Sinkin RA, Abramowicz JS: **Amnioinfusion during labor complicated by particulate meconium-stained amniotic fluid decreases neonatal morbidity**. *Am J Obstet Gynecol* 1994, **170**(3):842-849.

5. Gulmezoglu AM, Nikodem V, Hofmeyr GJ: **Amniotic fluid index changes after amnioinfusion**. In: *Proceedings of the 14th Conference on priorities in Perinatal care in South Africa 1995; South Africa*; 1995: 179-181.

6. Hofmeyr GJ, Gulmezoglu AM, Nikodem VC: **Amnioinfusion for meconium-staining of the amniotic fluid: A randomized trial**. In: *27th British Congress of Obstetrics & Gynaecology: 1995*; 1995.

7. Hofmeyr GJ, Gulmezoglu AM, Buchmann E, Howarth GR, Shaw A, Nikodem VC, Cronje H, de Jager M, Mahomed K: **The Collaborative Randomised Amnioinfusion for Meconium Project (CRAMP): 1. South Africa**. *Br J Obstet Gynaecol* 1998, **105**(3):304-308.

8. Macri CJ, Schrimmer DB, Leung A, Greenspoon JS, Paul RH: **Prophylactic amnioinfusion improves outcome of pregnancy complicated by thick meconium and oligohydramnios**. *Am J Obstet Gynecol* 1992, **167**(1):117-121.

9. Macri CJ, Schrimmer DB, Leung A, Greenspoon JS, Paul RH: **Amnioinfusion improves outcome in labor complicated by meconium and oligohydramnios**. *Am J Obstet Gynecol;* 1991, **164**:252.

10. Moodley J, Matchaba P, Payne AJ: **Intrapartum amnioinfusion for meconium-stained liquor in developing countries**. *Trop Doct* 1998, **28**(1):31-34.

11. Sadovsky Y, Amon E, Bade ME, Petrie RH: **Prophylactic amnioinfusion during labor complicated by meconium: a preliminary report**. *Am J Obstet Gynecol* 1989, **161**(3):613-617.

12. Sadovsky Y, Amon E, Bade ME, Petrie RH: **Prophylactic amnioinfusion during labor complicated by meconium: a preliminary report.** In: *Soc Perinatal Obstetricians Ninth Annual Meeting: 1-4 February 1999.; New Orleans.*; 1-4 February 1999.

13. Wenstrom KD, Parsons MT: **The prevention of meconium aspiration in labor using amnioinfusion**. *Obstet Gynecol* 1989, **73**(4):647-651.

14. Mahomed K, Mulambo T, Woelk G, Hofmeyr GJ, Gulmezoglu AM: **The Collaborative Randomised Amnioinfusion for Meconium Project (CRAMP): 2. Zimbabwe**. *Br J Obstet Gynaecol* 1998, **105**(3):309-313.
